# A smart energy service for the commercial rented sector: decision making points and business model considering the learnings from the pilot sites of the SmartSPIN project in Ireland, Spain and Greece

**DOI:** 10.12688/openreseurope.18851.1

**Published:** 2025-02-04

**Authors:** Luciano De Tommasi, Ruchi Agrawal, Padraig Lyons

**Affiliations:** 1 International Energy Research Center, Tyndall National Institute, University College Cork, Cork, County Cork, T12R5CP, Ireland

**Keywords:** smart energy service, energy efficiency, commercial rented buildings, CANVAS business model

## Abstract

**Background:**

This paper presents an evaluation of the smart energy service for the commercial rented sector developed during the European Commission’s Horizon 2020 funded research project SmartSPIN - Smart energy services to solve the SPlit INcentive problem in the commercial rented sector, highlighting the key decision-making points in relation to energy performance contracting in a rented commercial property.

**Methods:**

The perspectives of building owner, energy service company (energy service provider) and renters, are examined using a quantitative model that enables to evaluate the Net-Present-Value of the energy efficiency project for each party, considering the contract duration, energy cost savings, energy prices, investment costs and operation and maintenance costs, thereby highlighting the variables that determine the decision-making leading to the contractual agreements for the smart energy service. The business model for the proposed smart energy service is designed using the Business Model CANVAS template, whereas its value proposition is designed using the Value Proposition CANVAS template.

**Results:**

The proposed smart energy service has been implemented and evaluated by considering the experiences of the stakeholders and the feedback that the pilot participants of the SmartSPIN project in Ireland, Greece and Spain provided during the demonstration activities. This process enabled to identify improvements which eventually led to the business model validation highlighting the value creation for the ESCO businesses. Moreover, further leveraging the experience gained from activities at the SmartSPIN pilot sites and engagement with the key stakeholders, this paper also elaborates on how to create value for the customers.

**Conclusions:**

This paper shows how a smart energy service for improving the energy efficiency and flexibility in commercial rented buildings can be successfully designed and deployed, through a quantitative and qualitative analysis of its underlying business model, thereby overcoming the split incentive issue.

## 1. Introduction

### 1.1 Overview of smart energy services

Energy services have been defined as those functions performed using energy which are means to obtain or facilitate desired end services or states ( Fell, 2017). In fact, energy is desired not for its own sake, but for the services and utility that it can deliver, such as space and water heating and cooling, lighting, cooking, refrigeration, etc. The contribution of energy use to human well-being can be studied using the conceptual framework of Energy Service Cascade, which comprises structures, functions, services, benefits, and values ( Kalt *et al.*, 2019). *Structures* include the whole energy conversion chain from primary energy conversion to secondary, final and useful energy as well as building envelopes for space heating or road and rail infrastructures for freight and personal transport. *Functions* are the physical actions performed by an energy conversion chain. *Services* are the functions, or the aggregated functions demanded by humans and delivered to them. *Benefits* are all the positive outcomes that services deliver to humans. *Values* may be economic or non-economic. The economic value of an energy service is the willingness of humans to pay for it. Non-economic values include the habits and societal preferences that influence the demand for the service as well as the benefits that humans perceive from it.

Smart energy services are those ones that are based on smart technologies and/or innovative business models. Examples of smart energy services are energy supply and billing, monitoring and guidance, control and automation, energy trading, demand response, energy community services, smart home and smart metering set-up and support services, decentralized energy resources set-up and support services, integrated energy management ( Paukstadt, 2019). Integrated Energy Management Services need ICT platforms enabling access to the smart meter data and services such as energy monitoring, energy consumption prediction, resources management, energy optimization, billing, energy brokerage ( Karnouskos *et al.*, 2013). Big data platforms can effectively support the creation, development, maintenance and exploitation of smart energy services and data analysis techniques based on optimisation, forecasting, classification and clustering that support the design of innovative business models for stakeholders, including energy managers and consultants, energy service companies, city authorities and local administrations, utilities and energy retailers, construction companies, building components manufacturers, investors and project financiers, citizens ( Marinakis *et al.*, 2020).

Smart energy services can be classified considering their positioning within the value chain: generation and storage (i.e., asset management services such as real-time condition monitoring, predictive maintenance, remote control of assets, performance optimisation), trading and power system balancing (e.g., demand response and flexibility services, energy communities and peer-to-peer energy trading), retail (i.e., energy supply and billing), consumption (i.e., energy monitoring and management) ( Paukstadt & Becker, 2021). Provision of sustainable energy services may support the development of smart energy grids by means of a management model which ensures a balance between production, distribution, supply of renewable energy and rational consumption of renewable energy across different segments of consumers ( Borysiak & Brych, 2021). However, despite the progress of technology and the potential benefits that can be achieved, some consumers may be hesitant to adopt smart energy services or even actively resist to them, because of mistrust towards service providers, lack of understanding of costs and benefits, fear of becoming dependent or simply because of disinterest and disenchantment ( Kahma & Matschoss, 2017). Moreover, similar barriers (lack of interest, awareness, knowledge and human and financial capacity) may prevent market actors and stakeholders to engage successfully and to design and operationalise smart energy services based on advanced business models such as Energy Performance Contracting (EPC) ( Manfren *et al.*, 2021). Information hubs, use of ICT technologies, marketing and information campaigns for consumer engagement, can help to reduce barriers on information ensuring that flows of information on dynamic tariffs, prices and services are clear for the market players and end consumers ( Shivakumar *et al.*, 2018).

### 1.2 Evolution and trends in smart energy services across Europe: from research to deployment

A smart energy service is delivered through smart devices and a connectivity technology that enables customers to control equipment and appliances ( Gonçalves *et al.*, 2020). Moreover, information and communication technologies may be used to improve energy efficiency in smart buildings, fostering a behavioural change of the occupants determining a change of occupants’ daily energy consumption patterns. In fact, sensor data analysis and energy/behavioural analytics enable development of targeted recommendations, bespoke actions and decisions that determine a behavioural change of occupants and energy efficiency increase ( Fotopoulou *et al.*, 2017).

Smart energy services may also address the integration of plug-in electric vehicles (PEV) charging within the smart electricity grid through Vehicle-to-Grid (V2G) and Grid-to-Vehicle (G2V) services which promote the transportation services while reducing the carbon dioxide (CO _2_) emission from the transportation sector ( Anthony Jnr, 2021). V2G are demand response services that PEV sell to the electricity grid, which enable to discharge PEV’s batteries based on different signals that depend on energy production or consumption in the neighbourhood. G2V are flexible charging services which allow PEV owners to choose optimal charging schedules based on electricity prices or grid conditions.

A recent trend observed in smart energy services is the co-creation of advanced energy management solutions with the participation of customers in the development and assembly of their energy management system. On the other hand, electric vehicles’ owners cocreate value for smart energy/mobility services by sharing their experiences and recommendations with other interested persons. Some other customers are willing to adopt smart energy services only if they do not need to spend time and effort in using them ( Gonçalves *et al.*, 2020).

Moreover, new energy trading services are emerging on the markets. Examples of this are medium and long-term power trading, spot power trading, power auxiliary services, and carbon emissions rights trading, among others. Such services require key enabling technologies such as blockchain technology-based transaction mechanism, risk management, and platform design ( Xie *et al.*, 2021).

A blockchain is a distributed ledger system that can store data on a decentralized network in a transparent manner, which can be used for tracking the transaction of assets (such as a unit of energy traded) that are involved in peer-to-peer trading of electricity within an interconnected grid ( IEEE, 2024).

Innovation in smart energy services should focus on addressing both technical and business challenges. Innovation on the technology side should be accompanied by a revision of the traditional business models of the energy sector that need to be adapted to account for the emerging smart grid context, of which smart buildings are be part of ( Alvarez *et al.*, 2015).

Like traditional ESCO services, the smart energy service considered in this paper includes a variety of activities, such as energy analysis and audit, equipment supply, energy management, project design and implementation, monitoring and evaluation of energy savings, maintenance and provision of training for energy efficient system operation ( Bertoldi *et al.*, 2006). However, the proposed smart energy service builds on an innovative business model, which enables to make it suitable for deployment in commercial rented properties. Many commercial buildings in Europe show a large potential for implementing active retrofitting measures with short payback time using Energy Performance Contracting ( Garrido-Marijuan *et al.*, 2024).

### 1.3 Structure of the paper

This paper is structured as follows. Section 1 aims to provide a general introduction to the theme of smart energy services. Section 1.1 is a compact overview of the different smart energy services, which discusses different features found in smart energy services referring to the published literature. Section 1.2 expands the overview of 1.1 describing more recent developments and trends occurring in the field. After the introduction, the authors introduce the research context and the methodology. Section 2.1 presents an analysis of the barriers to the deployment of smart energy services in commercial rented buildings (i.e. the split incentive issue and the long contract duration) and the proposed solution, which is a tri-partite energy performance contracting business model. The factors influencing the decision-making process in relation to Energy Performance contracting for commercial rented buildings are discussed in Section 2.2. This section introduces the calculations of the Net-Present-Value for the building owner, tenants and Energy Service Company (ESCO) and the objectives that each party would pursue in the contractual agreement, if sufficient information would be available. Section 2.3 and Section 2.4 introduce respectively the Business model and Value proposition CANVAS templates, which have been used for the design of the proposed business model. Section 3 includes the results of the performed analysis building on the previous Section 2. Section 3.1 discusses the factors that determine the decision-making for the efficiency provider, building owner and renters (divided into 3.1.1, 3.1.2, 3.1.3). Section 3.2 discusses the factors that determine the contract duration. Section 3.3 discusses the practical implementation of the business model for the proposed smart energy service for the commercial rented sector, using the Business Model Canvas and the Value Proposition Canvas templates (respectively Subsection 3.3.1 and Subsection 3.3.2). Section 4 concludes the paper.

## 2. Research context and methodology

### 2.1 Analysis of barriers to the deployment of smart energy services in commercial rented buildings and a possible solution

Once that the factors determining energy efficiency in a commercial building (such as weather, occupancy, building systems design, building management system, energy management) are well understood ( Ismail & Inambao, 2019), several opportunities for energy efficiency improvement projects and measures can be easily identified ( Garrido-Marijuan *et al.*, 2024). However, their possible implementation using performance-based service models such as energy performance contracts (EPC) has been hindered by the complexity of such type of contract, and more specifically by barriers like the long contract duration of EPCs and the split incentive issue. In many cases, the contract duration of an EPCs is between 8 and 15 years, which may often be longer than the tenancy contract. A split incentive issue occurs when, e.g., in a rented building, the occupiers pay the energy bills, and the building owner is deterred to invest in energy efficiency measures because the benefit of reduced energy bills would accrue only to the buildings’ occupiers. The SmartSPIN project has developed an innovative business model based on the Energy Efficiency-as-a-Service (EEaaS) concept, which can effectively remove the barriers that hinder the adoption of the performance-based models in the commercial rented sector and validated this approach in four pilot buildings in Spain, Greece, and Ireland.

The SmartSPIN business model combines an energy efficiency service delivered by an energy efficiency provider (energy service company) through the installation and maintenance of energy efficiency measures (such as optimization of heating & cooling schedules, upgrades to lighting systems, upgrade or replacement of heating, cooling, ventilation and air conditioning (HVAC) equipment, building envelope improvement) with a demand side flexibility service, which enables to shift flexible consumption to the hours of the day where the electricity is cheaper ( Ring *et al.*, October 2022). A new flexible tariff model has been developed ( Ring *et al.*, 2023). This model incentivises the shifting of the energy consumption from peak hours to non-peak hours of the day through a convenient tariff, which is applied if the energy consumers meet a certain level of energy flexibility. If the contractual level of flexibility is not met by the energy consumer, then a penalty is applied by the electricity supplier, resulting in a higher and less convenient cost for the consumed electricity. The SmartSPIN service delivers both energy and non-energy benefits to the stakeholders. The business model aims to split the value of the energy savings between all stakeholders (building owner, tenants and energy efficiency service provider) in a fair and transparent manner. This business model is implemented through two contractual templates, one for the energy efficiency service and another one for the flexible tariff ( Ring *et al.*, 2023).

A key role in delivering the energy efficiency service is played by the energy efficiency service provider. The energy efficiency provider (which is in most cases an energy service company) signs an energy performance contract with the building owner and the building occupiers and is in charge of installing and maintaining energy efficiency measures, measuring and verifying the energy savings achieved monthly (with respect to a previously agreed baseline) and bills the tenants (building occupiers) with a fee that represents the cost of implementing the bespoke energy efficiency service. The payment performed by the tenants to the energy efficiency provider enables to share part of the energy cost savings with the building owner, thereby incentivizing the investment in energy efficiency measures and solving the split incentive issue. In absence of the energy efficiency provider, the building owner might recover part of the costs of the retrofit through a service charge (or part of it) payable by the renters ( Dixon, 2014).

The energy service company (ESCO) delivers the energy efficiency service with the guaranteed savings formula, which means that when the measured savings are lower than the contractual agreed savings, they will compensate their clients with the value of the energy savings shortfall, this way bearing the performance risk of the project ( De Tommasi *et al.*, 2024; Guo & Zhang, 2020; Shang *et al.*, 2017). The cost of the energy efficiency service for the tenants and the level of guaranteed savings for the building owner is determined by the parties’ bargaining.

Figure 1 depicts SmartSPIN business model involving a tri-partite agreement between building owner, tenants and ESCO, where the building owner is financing the implementation of proposed energy efficiency measures through their own sinking fund, while the ESCO is delivering guaranteed savings to their clients and is billing the tenants for the costs of the service. The revenue stream determined by the tenants’ payments is used to reward the building owner, that otherwise would not be incentivised in investing in energy efficiency measures, because the benefits of such investment, i.e., a lower energy bill, would be reaped by the tenants only (we assume, for simplicity, that the building owner does not occupy any unit in the building). Detailed recommendations for overcoming the split incentive issue were identified in ( Castellazzi *et al.*, 2017; Ring *et al.*, August 2022). Figure 1 also highlights the possible rebound effect, which consists in an increased energy consumption by the tenants if they are not rewarded with part of the energy savings obtained. This paper aims to raise awareness of building owners and ESCOs in relation to such rebound effect, as if not considered during the decision-making process that leads to a performance contract signature, they could realise too late that energy savings do not meet the expectations, because a change in the energy consumption patterns of building renters has occurred ( Berkhout *et al.*, 2000; Sorrell *et al.*, 2009). It is worth noting that the contract types which have the potential to resolve the rebound effect directly have more limited applicability with respect to EPC. The first type of performance-based contract which can directly eliminate the rebound effect references post-project technology implementation rather than the client’s energy consumption ( Ding *et al.*, 2017). This contract offers lower guarantees of performances to ESCOs’ clients with respect to EPC and it is therefore less attractive for potential clients. The second type of contract that eliminates the rebound effect is the Chauffage, where ESCO takes the complete responsibility of the energy services delivered to its client, e.g., space heating and lighting, including the full responsibility of purchasing fuel and electricity as needed ( Bhattacharjee *et al.*, 2010). With the Chauffage contract the ESCO will also control the client’s comfort level, whereas energy consumption is determined by the comfort level agreed in the contract ( Ding *et al.*, 2017). The main weakness of the Chauffage is that the customer is less incentivised to save energy with respect to other contractual models including a shared savings agreement for some parts of the energy savings ( Wargert, 2011), like the one described in Section 2.2 of this paper. Moreover, Chauffage contracts are typically very long (20–30 years) and therefore not suitable for the commercial rented sector ( Bhattacharjee *et al.*, 2010).

**Figure 1.**
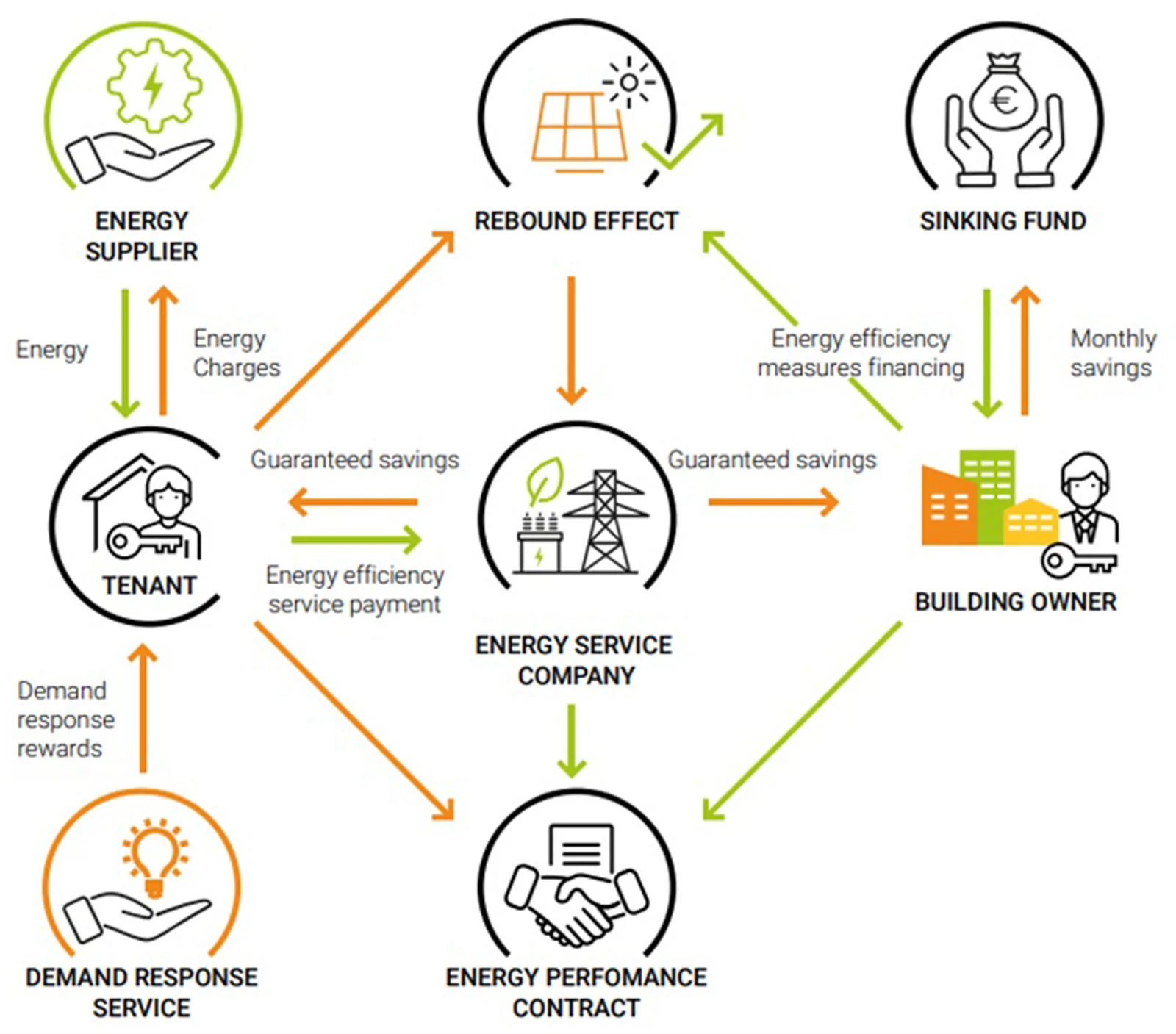
SmartSPIN Business Model with Rebound Effect.

In another variation of the business model depicted in Figure 1, the building owner funds the energy efficiency services by a taking loan from bank or any third party and the full responsibility for the loan repayment. Moreover, the energy efficiency service providers/ESCOs could fund the proposed upgrades taking the responsibility of a loan ( Figure 2). Another difference between the business models illustrated in Figure 1 and Figure 2, is that when the ESCO does not finance the EPC project, it will normally guarantee a certain level of energy savings to its client (guaranteed savings model, Figure 1), whereas when the ESCO finances the EPC project, it will share the energy savings with its client, taking a fixed portion of them over a fixed period (shared savings model, Figure 2).

**Figure 2.**
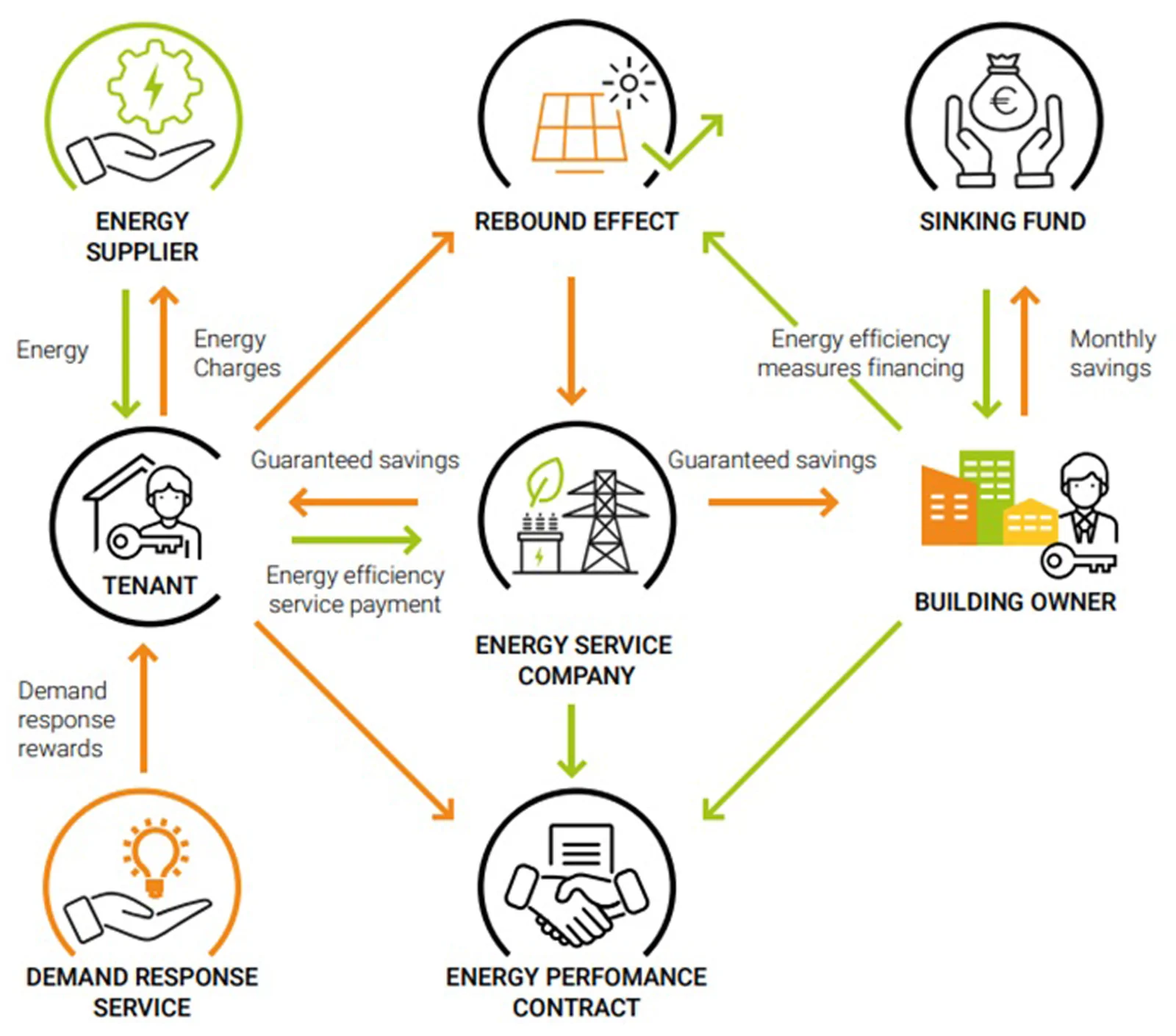
SmartSPIN Business Model (Energy Service Company liaising with Bank or Lender).

The implementation of the energy efficiency measures can be conveniently scheduled in two different stages (called implementation period/guarantee period) as depicted in Figure 3 (see also Agrawal *et al.*, 2022). In the first implementation or guarantee period, the energy efficiency service provider/ESCO engages with tenants and implements no-cost/low-cost measures. These measures include all the basic actions that can improve energy efficiency in commercial buildings in operation, which normally do not require permission from the building owner ( Escrivá-Escrivá, 2011). The first implementation period aims to demonstrate how the service works and to build the trust of the clients in the ESCO, while a decision to install medium/high-cost measures is taken by the building owner. During the second implementation/guarantee period, medium and/or high-cost measures (which require permission and a capital investment) are installed. The second implementation period aims to obtain high energy savings and reduce greenhouse gas ( Kneifel, 2010; Liang *et al.*, 2022), while reducing the performance risk for the building owner.

**Figure 3.**
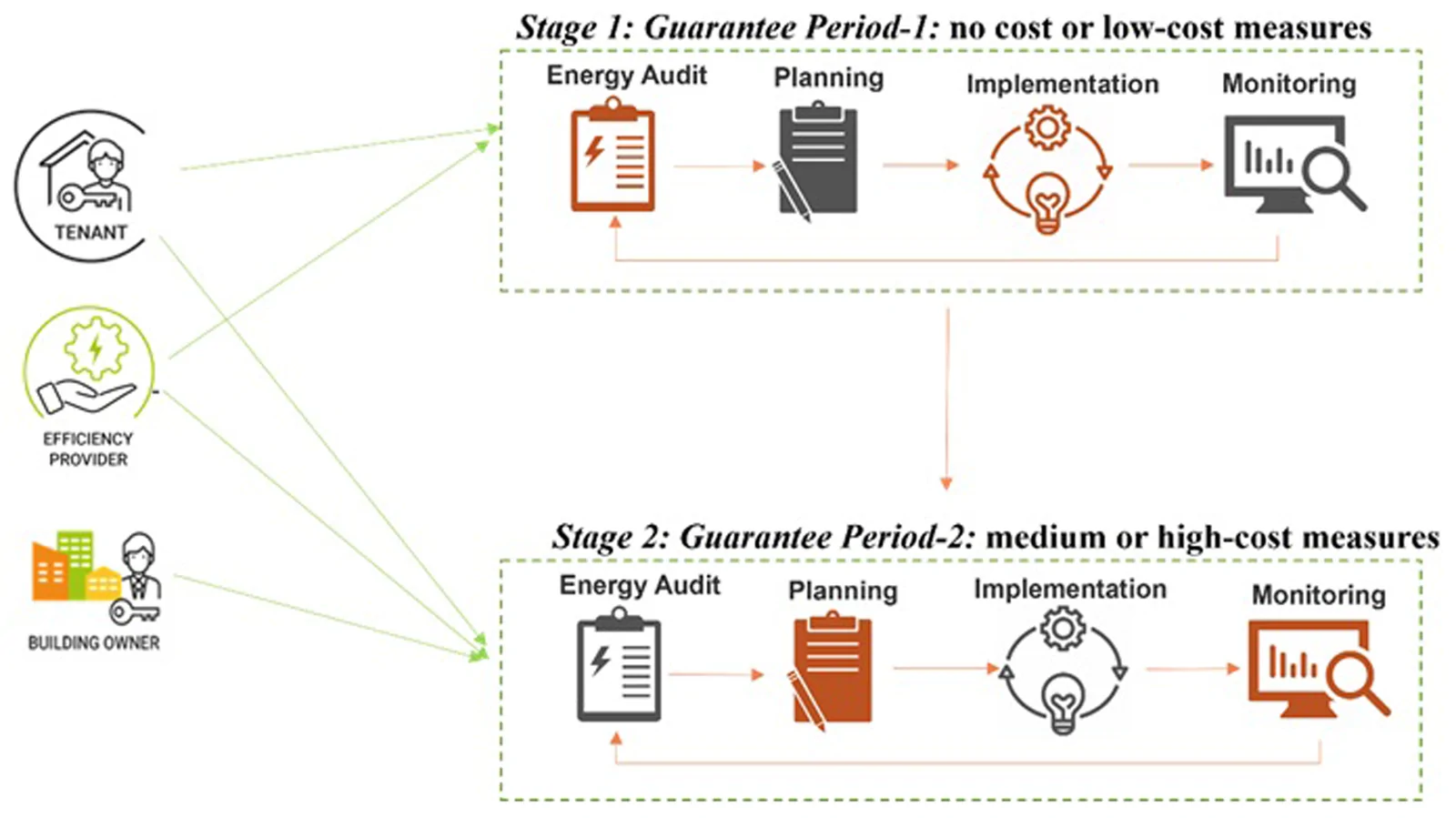
Multi-stage implementation of SmartSPIN Smart Energy Service.

### 2.2 Factors influencing the decision-making process in relation to Energy Performance contracting for commercial rented buildings

The most important factors that determine the decision-making process in relation to Energy Performance Contracting for commercial rented sector are: 1. the investments required for the energy efficiency measures and the party that bears it (typically, either the building owner or the ESCO); 2. the envisaged (monthly) energy savings; 3. the cost of the service for the end users (i.e., the service fee paid by the tenants); 4. The division of the energy savings achieved between building owner, tenants and energy efficiency provider; 5. The duration of the energy performance contract; 6. Other aspects such as maintenance costs, energy prices and degradation of equipment performance due to aging.

A simulation-based decision model for designing contract period duration in energy performance contracting was proposed in ( Deng *et al.*, 2014). The simulation model accounts for Energy efficiency investment (capital and O&M) as well as energy cost savings (which depend on the energy savings amounts and on the energy prices) and assumes that the variations of energy savings and energy prices over time can be modelled with a Geometric Brownian Motion (GBM) process ( Feng *et al.*, 2022). We also do such assumption in this paper. One limitation of the study ( Deng *et al.*, 2014) that we overcome in this paper is that the rented scenario is not considered; hence, only the variations of the Net-Present-Value for the building owner and the ESCO are considered, without accounting for the possibility of including renters and their perspective on the EPC.

Lu, Zhang and Chen proposed an optimization model that determined the contract duration such that the *NPV _O_* (Net Present Value) of the investment in energy efficiency for the building owner is maximised under the constraint that the *NPV _E_* for the ESCO is greater than zero ( Lu *et al.*, 2017). They assumed that the ESCO is the party performing the investment and guaranteeing a certain level of energy savings to the building owner and tenants; the energy savings are shared between them according to the proportion which maximise the landlord’s reward considering the rebound effect for the tenants.

We argue that if the ESCO is performing the whole initial capital investment in energy efficiency measures the contract duration will be normally long, and that may hinder the actual deployment of the energy performance contract due to the risk that tenants (who pay the service fee to the energy efficiency provider, thereby enabling the EPC to run) would leave the property before end of the EPC. To mitigate such risk, the investment could be shared between the building owner and the ESCO in a proportion that is acceptable for the building owner and that allows the ESCO to achieve their targeted profit with a shorter contractual duration. Considering the revenues for the ESCO, renters and building owner respectively given in Equation (1), Equation (2) and Equation (3), the NPVs for ESCO, renters and owner are provided in ( 4), ( 5) and ( 6). *I _OM_* ( *t*) in ( 4) is the cost of the operation and maintenance services. The main difference between the model proposed in this paper and the one in ( Lu *et al.*, 2017) is the fact that the investment in energy efficiency measures performed at *t* = 0 is shared between the ESCO and the building owner according to a factor *γ*, such that the building owner invests *γI _C_* whereas the ESCO invests (1 – *γ*) *I _C_* (these investments are negative cash flows at *t* = 0 for the calculation of NPVs). When *γ* = 1 the building owner is bearing the full investment in energy efficiency measures, whereas when *γ* = 0 the ESCO bears the full investment, which is the case considered in ( Lu *et al.*, 2017). The duration of the contract *n* that appears in ( 4) corresponding to a small *γ*, is greater than the duration corresponding to a large *γ*, because the positive cash flows must be accumulated to compensate a higher negative cash flow at *t* = 0.

$$R_{E}=\{\begin{array}{c} \;0,\;t=0\\ R(t)-G-\max[0,\beta (R(t)-G)],\;t=1,\;...\;n\\ \;0,\;t=n+1,\;...\;N \end{array}\;(1)$$

$$R_{R}=\{\begin{array}{c} \;0,\;t=0\\ (1-\theta )\;(G+\max[0,\beta (R(t)-G)]),\;t=1,\;...\;n\\ \;(1-\theta )R(t),\;t=n+1,\;...\;N \end{array}\;(2)$$

$$R_{O}=\{\begin{array}{c} \;0,\;t=0\\ \theta \;(G+\max[0,\beta (R(t)-G)]),\;t=1\;,\;...\;n\\ \;\theta R(t),\;t=n+1,\;...\;N \end{array}\;(3)$$

$$NPV_{E}=(\gamma -1)I_{C}+\sum_{t=1}^{n}\frac{R(t)-G-max[0,\beta (R(t)-G)]-I_{OM}(t)}{{\left(1+r_{E}\right)}^{t}}\;(4)$$

$$NPV_{O}=-\gamma I_{C}+\sum_{t=1}^{n}\frac{\theta \;(G+max[0,\beta (R(t)-G)])}{{\left(1+r_{O}\right)}^{t}}+\sum_{t=n+1}^{N}\frac{\theta R(t)-I_{OM}(t)}{{\left(1+r_{O}\right)}^{t}}\;(5)$$

$$NPV_{R}=\sum_{t=1}^{n}\frac{\left(1-\theta )\;(G+\text{max}[0,\beta (R(t)-G)]\right)}{{\left(1+r_{R}\right)}^{t}}+\sum_{t=n+1}^{N}\frac{\left(1-\theta )R(t\right)}{{\left(1+r_{R}\right)}^{t}}\;(6)$$

The most appropriate contract duration *n* is the one which maximises *NPV _O_*. *NPV _O_* is maximised when both the share of the initial investment in energy efficiency measures *γ* for the building owner and the contract duration *n* are minimised. However, reducing *γ* has the effect of increasing the amount of the investment which must be performed by the ESCO and therefore increases the contract duration. The selection of the best contract duration and investment share between the landlord and ESCO can be formulated as in Equation (7) subject to the constraints ( 8) and ( 9). Compared to the approach in ( Lu *et al.*, 2017) the optimisation approach introduces an additional variable, the share of the investment *γ* that is performed by the building owner. The constraint ( 8) states that the investment performed by the building owner is lower or equal to the maximum feasible investment for the building owner *I _max,C_*. The constraints ( 9) states that the NPV for the ESCO must be greater or equal to the minimum acceptable NPV for the ESCO.

$$\;n_{opt},\gamma_{opt}=arg\;\max_{n,\gamma }NPV_{O}\;(7)$$

subject to:

$$\;\gamma I_{C}\leq I_{max,C}\;(8)$$

$$\;NPV_{E}\geq NPV_{E,min}\;(9)$$

It is remarked that the formulation in ( Lu *et al.*, 2017), which assumes *γ* = 0, may result in a too long contract duration, which may make the EPC infeasible in practice due to the shorter duration of the rental contracts of tenants and the very high risk of not finding timely new renters who could pay the service fee to the ESCO to enable them to continue running the EPC. The optimisation process will increase *γ* and try to reduce *n* while satisfying both constraints ( 8, 9).

### 2.3 Business model CANVAS

The Business Model described in this paper is developed using the CANVAS template ( Table 1), which is a comprehensive visual chart proposed in ( Osterwalder & Pigneur, 2010), that covers all the aspects of the Business Model. Such template is a strategic management and entrepreneurial tool which allows to describe, design, validate a Business Model, highlighting its value proposition, infrastructure, customers and financial aspects. The Business Model can be adopted by organisations of any size, ranging from large enterprises to small-sized start-ups. It shows how an organisation can successfully run a business related to delivering energy savings to commercial companies. The CANVAS template consists of nine building blocks, namely Key Partners, Key Activities, Key Resources, Cost Structure, Value Proposition, Customer Relationships, Channels, Revenue Streams, Customer Segments. The four building blocks on the left-hand side of the CANVAS provide information about the efficiency-side of the business model (Key Partners, Key Activities, Key Resources, Cost Structure) and offer insights on how to scale up the business efficiently. The five building blocks on the right-hand side represent the value (Value Proposition, Customer Relationships, Channels, Revenue Streams, Customer Segments), which is delivered to the right customers who are willing to pay for the service. The **key partners** represent mainly strategic alliances with non-competitors or even joint ventures to develop the proposed business. The **key activities** describe the most important actions that a company must do to make the business model working. The key activities are required to create and offer a value proposition (e.g., a reduced energy consumption, while maintaining the same level of satisfaction for the energy consumer), to reach the market of smart energy services, creating and maintaining effective customer relationships, and earning revenues. The **key resources** are the most important assets required to make the business model work. The key resources allow the ESCO to create and offer a value proposition, reach out to the market, maintain relationships with customer segments and earn revenues. The **cost structure** section describes all the costs incurred to operate a business model, to create and deliver value, maintain customer relationships, and generating revenues. The **value proposition** defines the bundle of products and services that create value for one or more customer segments. The **customer relationship** section describes the types of relationships that the energy efficiency service provider/ESCO establishes with specific customer segments, including the customer acquisition, customer retention, boosting sales and upselling. The **channels** section describes how the company that implement the business model communicates with and reaches its customer segments to deliver its value proposition. The **revenue streams** represent the cash that the ESCO generates for each customer segment. The **customer segments** define the different types of commercial buildings and companies that an energy efficiency service provider/ESCO aims to reach and serve. Distinct segments are characterized by common needs, common behaviour or other attributes. The profitability of the different market segments may be substantially different as it depends on the willingness of the customers to pay for the proposed product or service.

**Table 1.** Business model for the implementation of the smart energy service in commercial rented buildings.

**Key Partnerships** Materials and components suppliers and installers (smart devices, renewable energy sources, energy storage components and infrastructure, e-mobility infrastructure, smart monitoring & management platform). Building automation and technology installers. Building services engineering companies. Project financiers. Energy Performance Contracting Facilitators. Building services engineers and designers. Energy efficiency consultants.	**Key Activities** Recommend energy efficiency measures. Elaborate an implementation plan for energy efficiency measures. Define the energy efficiency service price (service fee paid by clients) and plan of payments. Implement agreed energy efficiency measures. Collect energy consumption data (system monitoring) and optimise the installed measures. Implement the energy performance contract. Measure and verify energy savings and determine recurring payments of clients. Perform system maintenance regularly. Train clients on how to use of their equipment and appliances to reduce energy consumption.	**Value Proposition** Increased value and greener image of the property after installation of energy efficiency measures and BMS upgrades. Renovated property and non-energy benefits such as increased indoor comfort (e.g. thermal comfort and air quality). Monthly service fee paid by the renters to the ESCO for the energy efficiency service. Monthly payment by ESCO to the building owner to share the value of the energy savings achieved by renters. The service fee paid by the building owner to the ESCO (optional, applicable only if the building owner occupies part of the building). The responsibility taken by the ESCO for the installation, commissioning, monitoring and regular maintenance of equipment, devices and systems and the provision of guaranteed/shared energy savings to their clients.		**Customer Relationship** Agreement/SmartSPIN Contract between ESCO and clients about energy efficiency measures to be installed and the subject responsible for project financing (ESCO or building owner). Negotiation about the recurring service fee payment performed by each client to the ESCO. Invoice for the energy efficiency service sent by ESCO to clients. Receipt for the recurring payment received by the building owner from the ESCO.	**Customer segments** Building owners and renters of different typologies of rented commercial properties, such as: - Industrial buildings (e.g. manufacturing facilities, warehouses, etc.) - Retail (e.g. clothing stores, commercial banks, restaurants, etc.) - Offices - Healthcare facilities - Hotels and hospitality buildings - Special or mixed purpose buildings (e.g., airports, shopping centres, recreational centres etc.)
	**Key Resources** Capital from project financiers and government grants for the installation of energy efficiency measures. Contractual templates to implement the smart energy service in commercial rented properties. Energy efficient equipment and measures. Technologies for smart controls. Building Management Systems. Meters, sensors, data loggers. Gamification app, building performance diagnostic app, measurement and verification app.			**Channels** Website and blog. Printed posters displayed at strategic locations. Printed or digital flyers or brochures. Social media channels. Printed or emailed newsletters. Press releases. TV or radio advertisements. Outreach at trade shows and sectoral events. Outreach via building owners and managers associations. Business referral.	
**Cost Structure** Purchase, installation and financing cost of energy efficient equipment. Operation and Maintenance costs of installed equipment. Fees of consultants, contractors and ESCO personal. Costs associated with the measurement and verification of energy savings. Recurrent monetary reward to the building owner. Marketing and advertisement costs.			**Revenue Streams** Main revenue stream associated with the share of energy cost savings obtained by the clients, i.e., renters and building owner (the latter only if they occupy part of the building). Revenue stream for the operation and maintenance service. Additional revenue stream for equipment rent, in case the ESCO owns the equipment and rents it to the client for a fixed monthly fee. All the revenue streams are collected through the service fee.		

### 2.4 Value Proposition CANVAS

The business model CANVAS described in the previous Section 2.3 provides the necessary information to the businesses (energy efficiency providers, ESCOs or similar) that want to implement the smart energy service in rented commercial buildings. To design and analyse the value proposition of the proposed product or service, the Value Proposition CANVAS template is used ( Osterwalder *et al.*, 2014). This task consists in determining how the benefits, features and experience with the considered smart energy service create value for the customers addressing their wants, needs and fears. The CANVAS template also includes specific information on the product/service, the profile of its ideal customer and the existing substitutes. The value proposition defines the bundle of products and services that create value for one or more customer segments. The value proposition is an aggregation of benefits that ESCOs offer to customers. The Value Proposition CANVAS is divided into two sections: Product/Service and Customer. The former section is divided into benefits, features, experience, product and ideal customer sections. The latter section is divided into wants, needs, fears and substitutes. The benefits section includes the benefits delivered by the proposed service to the customer, that make customer’s operations easier by increasing satisfaction or decreasing discomfort. The **benefits** of the proposed smart energy service are the core of its value proposition. The features are the part of the value proposition consisting in the functional attributes that factually describe how the proposed smart energy service works. The features are the “reasons to believe” for the customer. The **features** are the part of the value proposition consisting in the functional attributes that factually describe how the proposed service works. The features are the “reasons to believe” for the customer. The service **experience** is the way that receiving the service makes the customer feel. The customer’s experience results from the service’s features and benefits. The service experience differs from features and benefits because it relates more to the emotional reasons why customers want to purchase the service and the value it delivers to them for their own businesses. The service experience can be used to identify how the proposed service should be positioned in the market and how the brand should communicate the value proposition to the potential customers. The ‘product’ section states what product or service is described in the Value Proposition CANVAS. The ‘ideal customer' section states who the ideal customer for the proposed product or service is. **Wants** are the customer’s thoughts about how they would like to improve the activities in their business that are relevant with the considered product or service. They are the emotional drivers of decision-making that can motivate customer’s actions. The customer’s **needs** are the actions that the customer needs to get done. Customers might not be aware of all their needs. Needs that are not known yet by the customer yet are called “latent needs”. The customer’s needs can be determined following a rational reasoning. **Fears** are the hidden source of wants and needs for the customer and can be seen as a driver for purchasing the proposed service, if it addresses the wants and needs of the customer. Customers’ fears may also be related to the perceived efforts of switching service or service provider. To win such fear of change, the proposed service must deliver very significant improvements to overcome the reluctance of the customers to change their current processes. A **substitute** is a product or service that serves the same purpose as the product/service described in the Value Proposition CANVAS template.

## 3. Results

### 3.1 Factors that determine the decision-making for the efficiency provider, building owner and renters

***3.1.1 Factors that determine the decision-making for the energy efficiency provider***

The factors that influence the decision of an energy efficiency provider (ESCO) to enter an energy performance contract are: 1. Whether the building owner performs in part or totally the investment required to install energy efficiency measures; 2. The behaviours of the renters which may determine a rebound effect, i.e. an increment in their energy consumption following the installation of energy efficiency measures ( Berkhout *et al.*, 2000; Sorrell *et al.*, 2009); 3. The agreements between building owner and renters with respect to sharing the energy savings and the agreement between renters and energy efficiency provider in relation to the service fee to be paid for the energy efficiency service.

We will thoroughly analyse the case where the ESCO performs the investment, i.e., *γ* = 0 in ( 4) and ( 5), as that is the case where the decision making may become more complicated for the ESCO and at the same time it is the most attractive for the potential clients.

The actual savings *Q*( *t*) achieved by the energy efficiency project can be expressed as follows:

Q(t)=f(t)⋅Re(θ)⋅K(t)(10)

where *f*( *t*) is the equipment degradation function (which decreases from 1 to 0 during the project lifetime), *K*( *t*) represents the energy savings potential, *Re*( *θ*) is the rebound effect multiplier, *θ* is the share of the energy savings kept by the building owner. The rebound effect multiplier has the following form:

$$\;Re(\theta )=\alpha_{2}+\alpha_{1}e^{-\frac{k_{2}\theta +k_{1}}{\rho }}\;(11)$$

where $\alpha_{1}=\frac{\varphi }{1-e^{k_{1}/\rho }}$ and *α*_2_ = 1 – *α*_1_.The rebound effect is maximum when the building owner does not share any savings with the renters ( *θ* = 1). The ESCO’s decision of delivering the service and the duration of the performance contract agreed with its client depend on the actual energy savings *Q*( *t*) throughout the duration of the project, which in turn depend on the rebound effect *Re*( *θ*). With the model ( 11), the behaviours of the renters are modelled through the two parameters *φ* and *ρ*, which are respectively the maximum rebound effect (corresponding to *θ* = 1) and the risk tolerance, which accounts for the individual risk attitudes of different renters. The rebound effect multiplier *Re*( *θ*) is plotted out considering different values of *φ* and *ρ* in Figure 4 (assuming *k*_1_ = –100 and *k*_2_ = 200).

**Figure 4.**
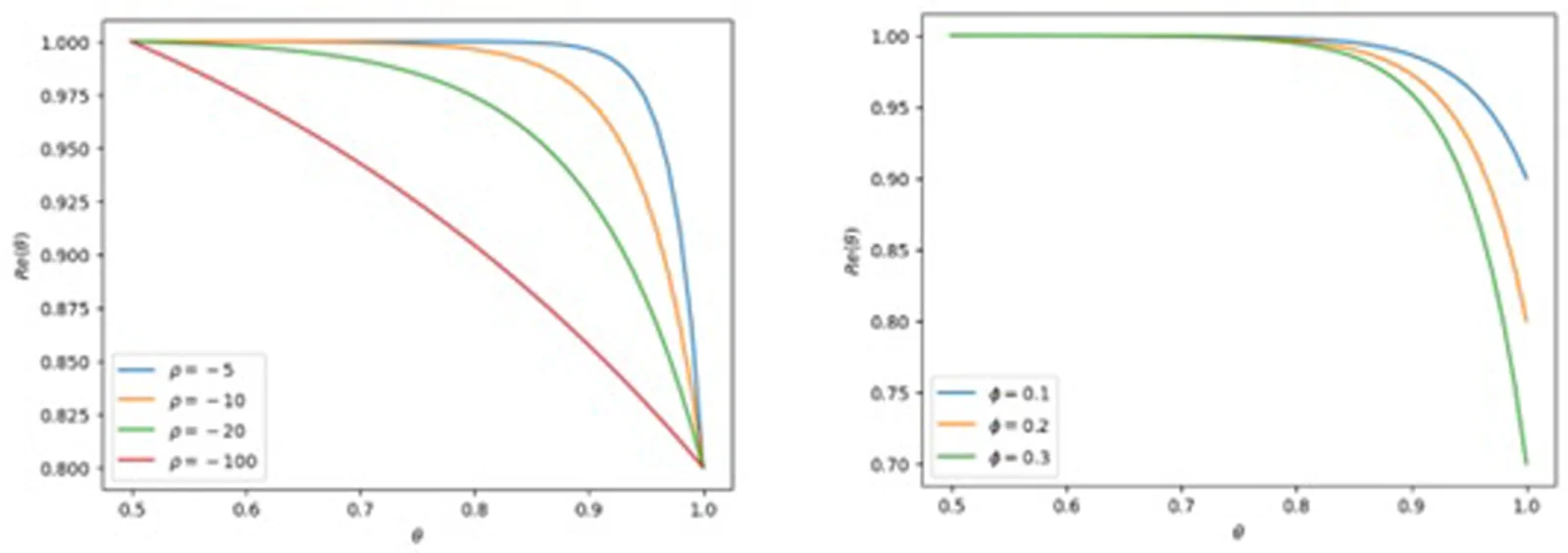
Variations of the rebound effect multiplier with *φ* and *ρ* parameters (respectively left-hand side figure and right-hand side figure).

It can be observed that when the percentage of the energy savings kept by the building owner *θ* increases, the rebound effect multiplier *Re*( *θ*) decreases lowering the *Q*( *t*); this effect may influence the decision of the ESCO to deliver the service as well as the minimum contract duration acceptable for them, which would fulfil the condition ( 9). An increment in *θ* determines a decrement in *NPV _E_* (due to the lower energy savings) for a given contract duration. Posing *NPV _E,min_* = 0 in ( 9), we may determine the maximum percentage of energy savings *θ _max_* which the building owner can keep while ensuring that the ESCO may deliver the service (i.e., *NPV _E_* does not become negative).

It is worth noting that an estimation $\tilde{R}(t)$ of the actual energy savings *R*( *t*) can be obtained as follows:

$$\;\tilde{R}(t)=Q(t)\cdot P_{E}(t)\;(12)$$

where *P _E_* ( *t*) is the future energy price, which is modelled through the Geometric Brownian Motion (GBM) stochastic model.

$$\;P_{E}(t)=P_{E0}e^{\left(\alpha_{E}-\frac{\sigma_{E}^{2}}{2}\right)t+\sigma_{E}\varepsilon_{P}\sqrt{t}}\;(13)$$

The initial energy price is *P*_*E*0_ = 0.24, the annual price drift is α *_E_* = 0.0523, whereas the annual price volatility effect σ *_E_* = 0.0856 is and the random error is *ε _P_* = 0.01. The energy savings potential *K*( *t*) is also modelled using a GBM process.

$$\;K(t)=I_{C}K_{0}e^{-\frac{\sigma_{K}^{2}}{2}t+\sigma_{K}\varepsilon_{K}\sqrt{t}}\;(14)$$

where the potential energy savings per unit of initial investment of retrofit is *K*_0_ = 0.43, the volatility coefficient is *σ _K_* = 0.01, the random error is *ε _K_* = 0.01.

In the following we consider the following parameters: *I _C_* = 3.84 M€; *G* = 250 k€/year; *β* = 0.2; n = 14, 15, 16, 17, 18, 19, 20, 21; N = 25; *θ* ranging from 0.5 to 1. The operation and maintenance costs can be modelled as a Geometric Brownian Motion (GBM) stochastic process ( QuantPy, 2024). We also follow that assumption ( Lu *et al.*, 2017) in ( 15).

$$\;I_{OM}(t)=\left(\frac{1}{\delta }\right)I_{C}e^{\left(-\frac{\sigma_{H}^{2}t}{2}+\sigma_{H}\varepsilon_{H}\sqrt{t}\right)}\;(15)$$

Where δ = 1.025, *σ _H_* = 0.25 and *ε _H_* =0.01; *γ* = 0 (the initial investment is performed by the ESCO). Moreover, *r _O_* = *r _R_* = 0.031; *r _E_* = 0.06.

The accuracy of the expressions ( 13), ( 14) and ( 15) has been validated comparing them against the outputs of an actual Geometric Brownian Motion stochastic process, using parameters matching those ones used in the expressions. It was found that they can predict very well the average output of a GBM simulation code ( QuantPy, 2024). These expressions are therefore suitable for the analysis proposed in this paper.

Results in Figure 5 show that especially for shorter contract durations, the ESCO should pay attention to the maximum rebound effect and the risk tolerance of renters, as when they are high, high values of the percentage of the energy savings kept by the building owner might determine economic losses for the ESCO. If these parameters cannot be estimated with satisfactory accuracy, the ESCO should propose a contract with longer duration to mitigate the risk of economic losses due to lower energy savings caused by renters’ rebound effect. The sensitivity of the maximum percentage of energy savings kept by the building owner which is acceptable for the ESCO *θ _max_* with respect to the variations of the risk tolerance *ρ* is even higher than the sensitivity with respect to the variations of the maximum rebound effect *φ* because *ρ* appears in the denominator of the exponent of the exponential function in ( 11), whereas *φ* is a multiplying factor of two terms of the sum in ( 11).

**Figure 5.**
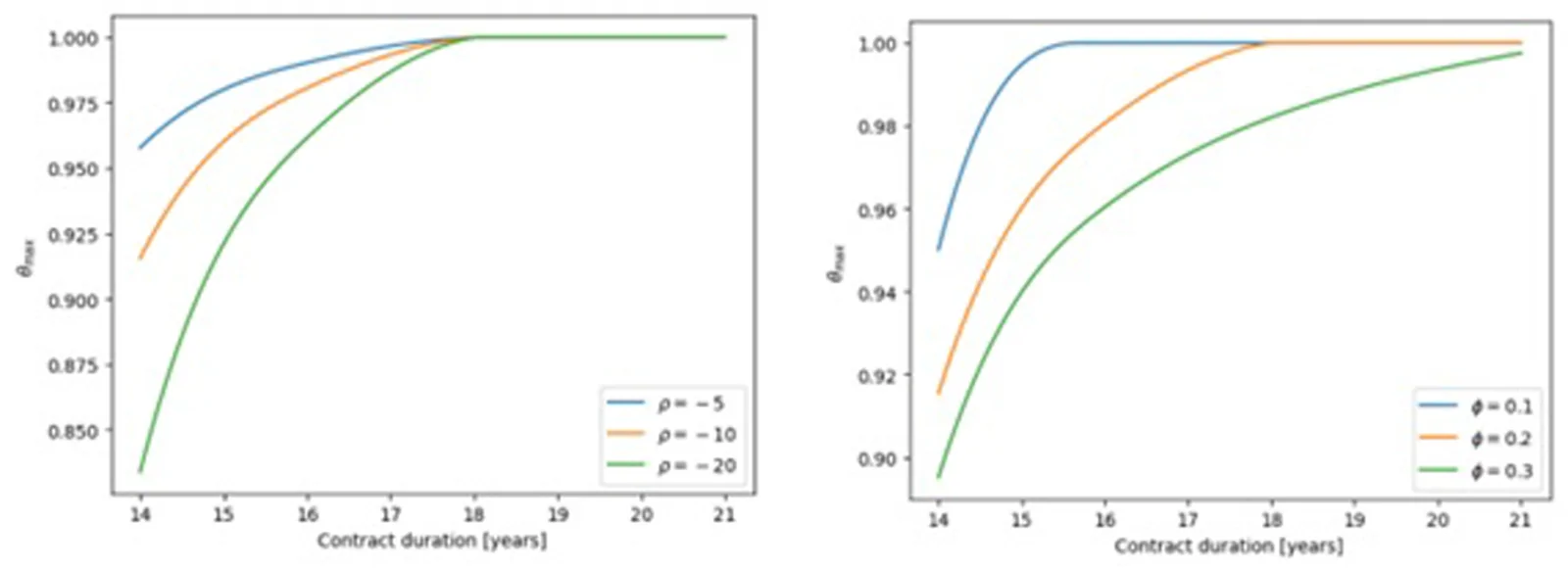
Maximum percentage of the energy savings kept by the building owner acceptable for the ESCO for different values of contract duration, *ρ* and *φ* (respectively left-hand side figure and right-hand side figure).

***3.1.2 Factors that determine the decision-making for the building owner***

We consider in this section the case where the building owner does not occupy any significant spaces in the building. In fact, if the building owner occupies the premises, wants to modernize the building and accrues some energy savings once that the energy efficiency measures are installed, the split incentive is a less significant issue and the payment of a service fee from the tenants may not necessarily be determinant with respect to the decision of investing in energy efficiency measures. Building owners tend to bargain for a shorter contract duration (as opposed to ESCOs that bargain for a longer duration) as they are aware that the energy efficiency measures installed can still produce additional savings beyond the length of the contract, which will not have to be shared with the ESCO after the end of the EPC ( Li *et al.*, 2014). Indeed, the objective of building owners is to maximise their NPV while contracting with an ESCO. It must be pointed out that that under an EPC, the ESCO takes the full responsibility for the maintenance of the installed equipment and provides training to its client to operate the systems at their best performances. During the contract term, faulty equipment is replaced at the ESCO’s own expense. Such a comprehensive service is appreciated by the building owners. Guo and Zhang proposed an approach to optimise the guaranteed savings value G that appears in equations ( 1– 6), which consists in finding the value *G _opt_* value that minimises absolute value of the difference between building owner’s investment and ESCO’s investment. The contract duration can be adjusted comparing the NPVs of the building owner and ESCO as it was done in ( Deng *et al.*, 2014); in addition, the perspective of the renters must be considered when the considered property is rented. This perspective will be analysed in the next Section 2.2.3. Another factor that needs to be taken into consideration when determining the EPC agreements is the share of energy savings with the renters. Under the assumption that the rebound effect in energy consumption of the renters is not negligible (which was put forward in the previous Section 2.2.1), the building owners need to take it into consideration to determine the optimal share of savings with the renters, i.e., (1 – *θ*) in Equation (2) and Equation (6). The optimal share of *θ _opt_* depends on the maximum rebound effect *φ* and the risk tolerance *ρ* (see also De Tommasi & Agrawal, 2023). It can be observed that when the rebound effect increases from *φ* = 0 to *φ* > 0 the NPV curves for the building owner (which are straight segments for *φ* = 0) start curving ( Figure 6). Increasing *φ* will lead to a decrease of *θ _opt_* and for *φ* > *φ*′ > 0 the maximum NPV will be obtained with a *θ _opt_* < 1 ( Figure 7).

**Figure 6.**
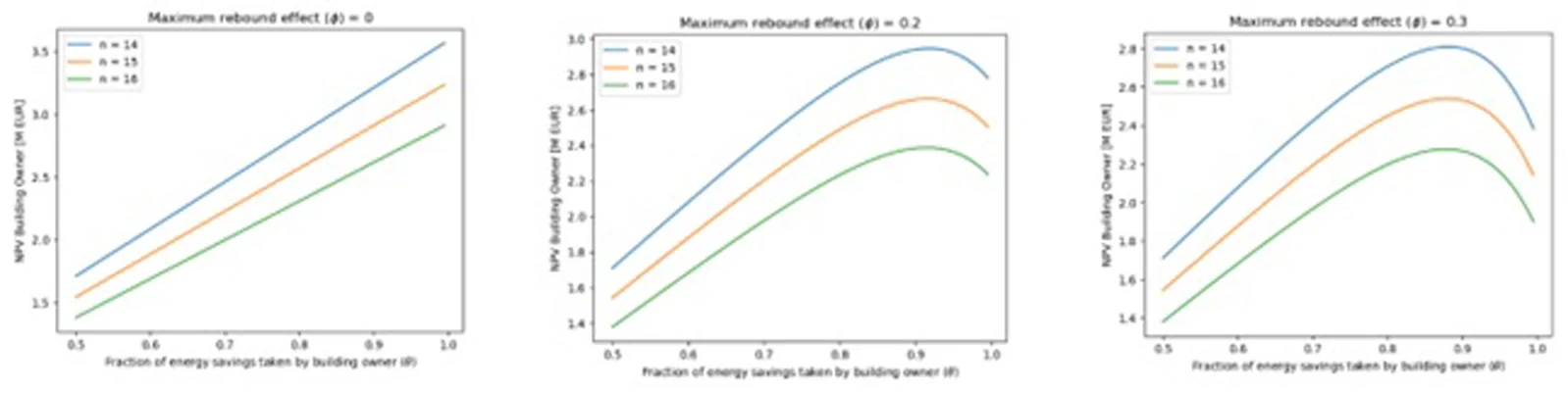
Net Present Value for building owner as a function of *θ* for different contract durations (n = 14, 15, and 16 years). Left hand side panel: case *φ* = 0. Centre panel: *φ* = 0.2. Right hand side panel: *φ* = 0.3.

**Figure 7.**
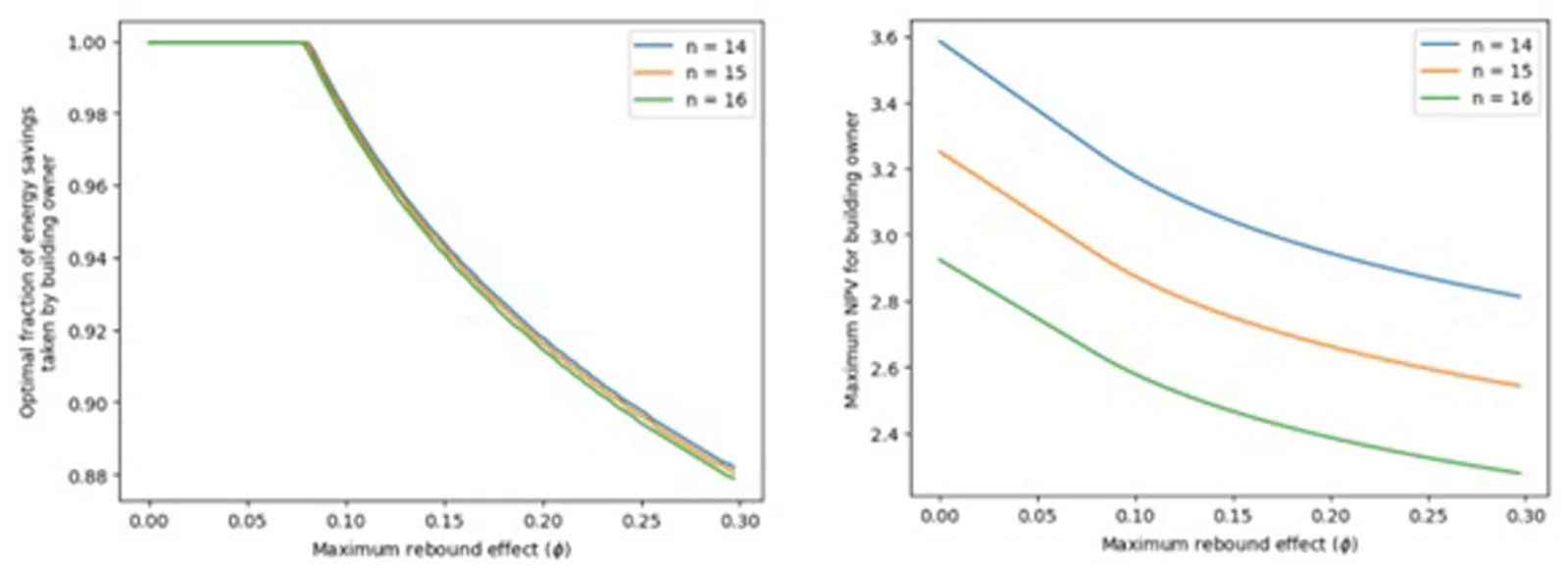
Left hand side panel: Optimal fraction of energy savings taken by building owner as a function of the maximum rebound effect *φ* for different contract durations (n = 14, 15, and 16 years). Right hand side panel: Maximum NPV for building owner as a function of the maximum rebound effect *φ* for different contract durations (n = 14, 15, and 16 years).

***3.1.3 Factors that determine the decision-making for the renters***

According to the business model illustrated in Figure 1 and Figure 2, the smart energy service delivers guaranteed energy savings to the renters; however, since the renters do not bear the investment costs of the energy retrofitting project, they are required to pay a service fee to the ESCO. The ESCO uses such payments to share the value of the energy savings with the building owner. Although there might be non-energy benefits delivered to the renters such as a renovated property or improved thermal comfort, a requirement in many rented commercial properties is that the service fee paid to the ESCO is not greater than the energy savings achieved. For simplicity, in this analysis we aggregate all the renters’ energy cost savings and service fee payments. The monthly energy costs before the retrofit (baseline) are calculated as follows:

$$\;E_{C,bas}=E_{el,bas}\cdot P_{el}+E_{ng,bas}\cdot P_{ng}\;(16)$$

where *E _el,bas_*, *E _ng,bas_*, *P _el_*, and *P _ng_* are respectively the baseline energy consumption of electricity, the baseline energy consumption of natural gas, the price of electricity per kWh, and the price of natural gas per kWh. The monthly energy costs after the retrofit are:

$$\;E_{C,ret}=E_{el,ret}\cdot P_{el}+E_{ng,ret}\cdot P_{ng}\;(17)$$

The energy cost savings are:

$$\;\Delta E_{C}=E_{C,bas}-E_{C,ret}\;(18)$$

The renters approve the EPC if:

$$\;\Delta E_{C}-S\geq 0\;(19)$$

where *S* is the total value of the monthly service fees paid by the renters to the ESCO. The difference ∆ *E _C_* – *S* represents the monthly energy cost savings shared with the renters:

$$\;12\cdot (\Delta E_{C}-S)=R_{R}\;(20)$$

where *R _R_* is the annual revenue stream for the renters ( 2). It is worth noting that if the service fee is set equal to the monthly energy bills savings ∆ *E _C_* = *S*, then there are no actual energy savings shared with the renters as they would pay the same amount saved on the monthly bills to the ESCO for the smart energy service (which would share that value with the building owner), that corresponds to setting *θ* = 1 in ( 2),( 3),( 5),( 6).

### 3.2 Factors that determine the contract duration

The long contract duration of Energy Performance Contracts is a barrier in most Member States EPCs to their penetration in the commercial rented sector because an EPC duration of 8–15 years (which is quite common) is in many cases longer than the tenancy contracts. The contract duration is determined by the initial investment performed by the ESCO, the energy savings generated by the energy efficiency measures *R*( *t*), by the guaranteed savings *G* provided by the ESCO to the customer, by the fraction of savings *β* shared with the customer above the threshold *G*, the share of the savings *θ* between building owner and renters. A higher investment for the ESCO will determine generally a longer contract duration to recover the investment and make a profit. On the other hand, higher energy savings *R*( *t*) will facilitate a contract of shorter duration, whereas higher guaranteed/shared savings ( *G* and *β*) will reduce the value of the savings taken by the ESCO and possibly determine a longer contract duration. The study reported in ( Collins *et al.*, 2018) found that in UK building owners and their investors are keen to invest in energy efficiency technologies in their new or retrofitted buildings despite the upfront costs, because of the long-term financial benefits as well as those associated with corporate social responsibility. The same study concludes that the main barriers for building owners and tenants with respect to the implementation of energy efficiency technologies lie in the bureaucratic and technical domains. Many published papers assume that either the ESCO or the building owner would take full responsibility of the initial investment in energy efficiency measures *I _C_*. In ( Li *et al.*, 2014), the investment share between ESCO and its client was considered as one of the EPC contract terms. However, the model proposed in ( Li *et al.*, 2014) did not consider a rented building. In this paper, we introduced the sharing factor *γ* in *NPV _E_* and *NPV _O_* (such that the ESCO invests *α* = 1 – *γ* whereas the building owner invests *γ*) considering the EPC model which applies to commercial rented buildings. *NPV _E_* can be increased reducing *α*, which allows to fulfil the condition *NPV _E_* ≥ *NPV _E,min_* with a shorter contract duration ( Figure 8). In other words, to mitigate the risk of tenants leaving before the end of the EPC, a building owner will have to take the responsibility of part of the initial capital investment and negotiate the appropriate contract duration with the ESCO.

**Figure 8.**
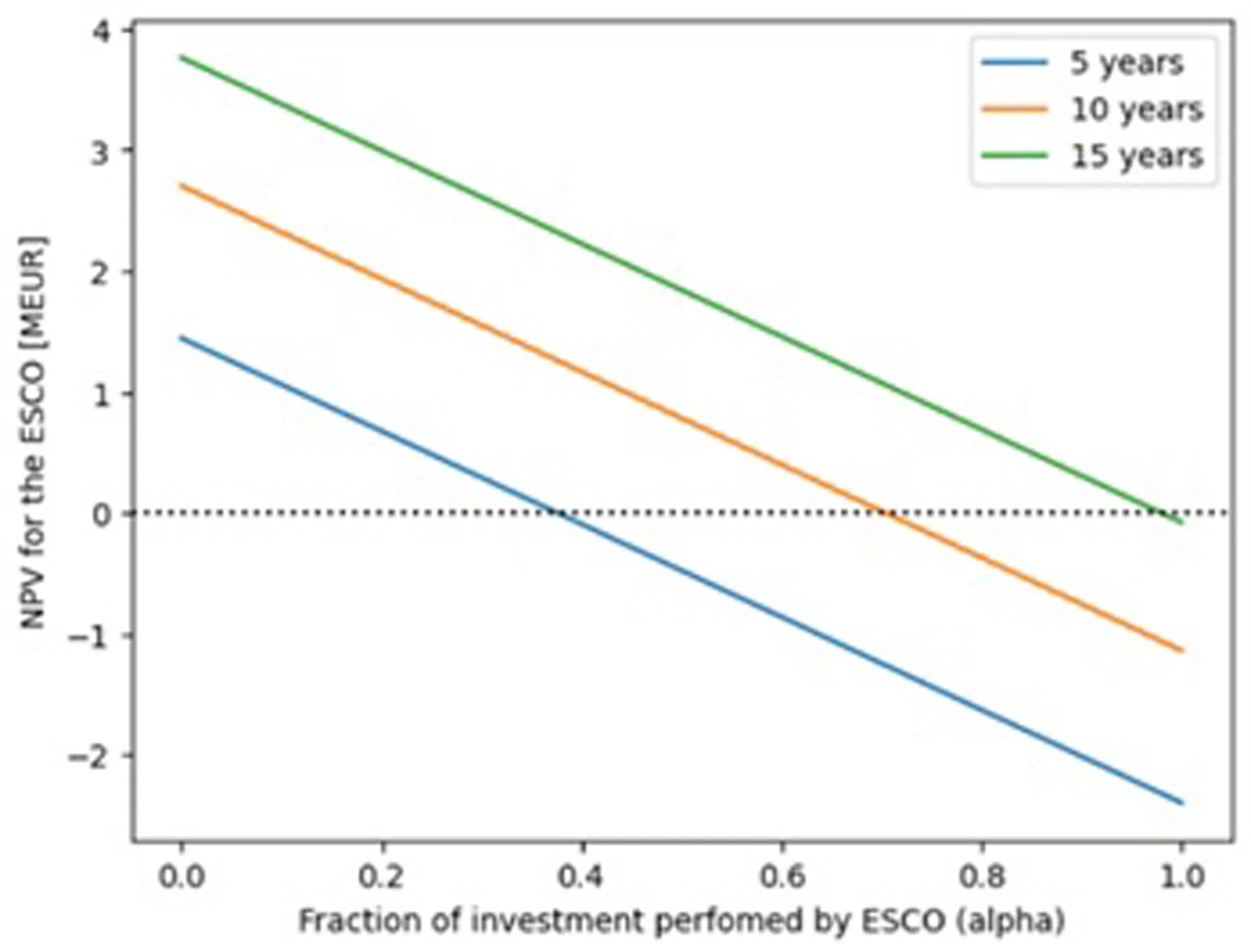
NPV for the ESCO as a function of the fraction of the investment performed by the ESCO and contract duration (5 year, 10 years and 15 years).

### 3.3 Practical implementation of a business model for a smart energy service for the commercial rented sector

***3.3.1 Business model***

This section discusses the development of the business model for the smart energy service for the commercial rented sector proposed in this paper, applying the business model CANVAS previously introduced in Section 2.3. Result of this analysis are presented in Table 1. The business model development discussed in this paper builds on the previous work in ( De Tommasi *et al.*, 2024; Ring *et al.*, October 2022; Skordoulias *et al.*, 2024).

*Key activities*

The key activities include the recommendation of energy efficiency measures, the elaboration of an implementation plan for energy efficiency measures, the determination of the energy efficiency service price (service fee paid by clients to the ESCO) and the plan of payments. They also include the actual implementation of agreed energy efficiency measures, the system monitoring performed collecting energy consumption data and optimising the functioning of installed measures. The key activities also include the implementation of the energy performance contract, the measurement and verification of energy savings ( Papadelis, 2023), and the determination of the recurring payments of clients. Furthermore, other key activities involve performing regularly the system maintenance and the delivery of training sessions to clients about how to best operate their equipment and appliances to achieve a reduced energy consumption.

*Customer relationship*

In the proposed business model, the customer relationship is based on specific contractual agreements between the ESCO and its clients concerning the energy efficiency measures that will be installed and the party who will take the responsibility for project financing (either ESCO or building owner). The contract allows to establish a tripartite agreement between ESCO, landlord and tenants ( Ring *et al.*, 2023). It also specifies the duration of the service and the service fee that the clients will pay to the ESCO. The renters are the party that normally pays the service fee to the ESCO, whereas the ESCO shares part of the value reaped from the renters with the building owner. In some cases, the building owner may also pay a service fee to the ESCO, e.g. if he occupies the building and directly reaps part of the energy savings. The customer relationship also includes the negotiation about the recurring service fee payment performed by each client to the ESCO, the invoice for the energy efficiency service sent by the ESCO to its clients and the receipt for the recurring payment received by the building owner from the ESCO.

*Customer Segment*

Examples of customer segments in the commercial sector, include building owners and renters of different typologies of rented commercial properties, such as shopping centres, industrial buildings, industrial buildings (e.g. manufacturing facilities, warehouses, etc.), retail (e.g., clothing stores, commercial banks, restaurants, etc.), offices, healthcare facilities, hotels and hospitality buildings, special or mixed purpose buildings (e.g., airports, shopping centres, recreational centres etc.). An effective segmentation of the customers in the commercial rented sector can be performed considering the number of buildings and size of a commercial property, the number of tenants that are renting, the surface occupied by the building owner compared to the total surface occupied by the renters. The other target customers could be facility manager and companies and energy users in the rented premised for running an industry or business. An ESCO adopting the business model in Table 1 might make a conscious decision about which segments to serve and which ones to ignore. It is likely that small ESCOs that have a simple structure, and few employees will focus on smaller clients, such as small or medium size office buildings. In this case the customer relationship will be built mainly with the building owner, with the renters be given a minor weight in the contractual negotiations and agreements, which might be limited to give the consent for the works that affect their premises and agree to pay the service fee. For other customer segments, the split incentive issue may be even more significant, the customer relationship would involve building owner, building renters and ESCO in a more balanced manner. The service offer is partially differentiated for the different customer segments; however, apart from different contractual clauses affecting the renters and agreements which ultimately affect the way how the parties divide the energy savings achieved between them, the service is substantially similar across different customers’ segments, in the sense that delivers an energy performance contract in a rented commercial building, with variable benefits for the occupiers, the building owner and the ESCO.

*Key Partners*

The key partners are materials and components suppliers and installers of smart devices, renewable energy sources, energy storage components and infrastructure, e-mobility infrastructure, smart monitoring & management platforms, building automation and technology installers, building services engineering companies, project financiers, Energy Performance Contracting facilitators, building services engineers and designers, energy efficiency consultants.

*Key Resources*

The key idea behind the proposed business model is to create value for both building owners and renters. The key resources needed for delivering the energy efficiency service include the capital from project financiers and government grants for the installation of energy efficiency measures, the contractual templates to implement the smart energy service in commercial rented properties, the energy efficient equipment and measures, technologies for smart controls, Building Management Systems, meters, sensors and data loggers, a visualisation dashboard for energy consumption monitoring ( Diez *et al.*, 2024), a gamification app ( Iria *et al.*, 2020), building performance diagnostic app ( Eguiarte *et al.*, 2023), measurement and verification app ( Gallagher *et al.*, 2019). The visualisation dashboard and gamification app developed by the Smarkia in the H2020 SmartSPIN project are illustrated respectively in Figure 9 and Figure 10. The key resources can be owned or leased by the ESCO or building owner or made available by the key partners.

**Figure 9.**
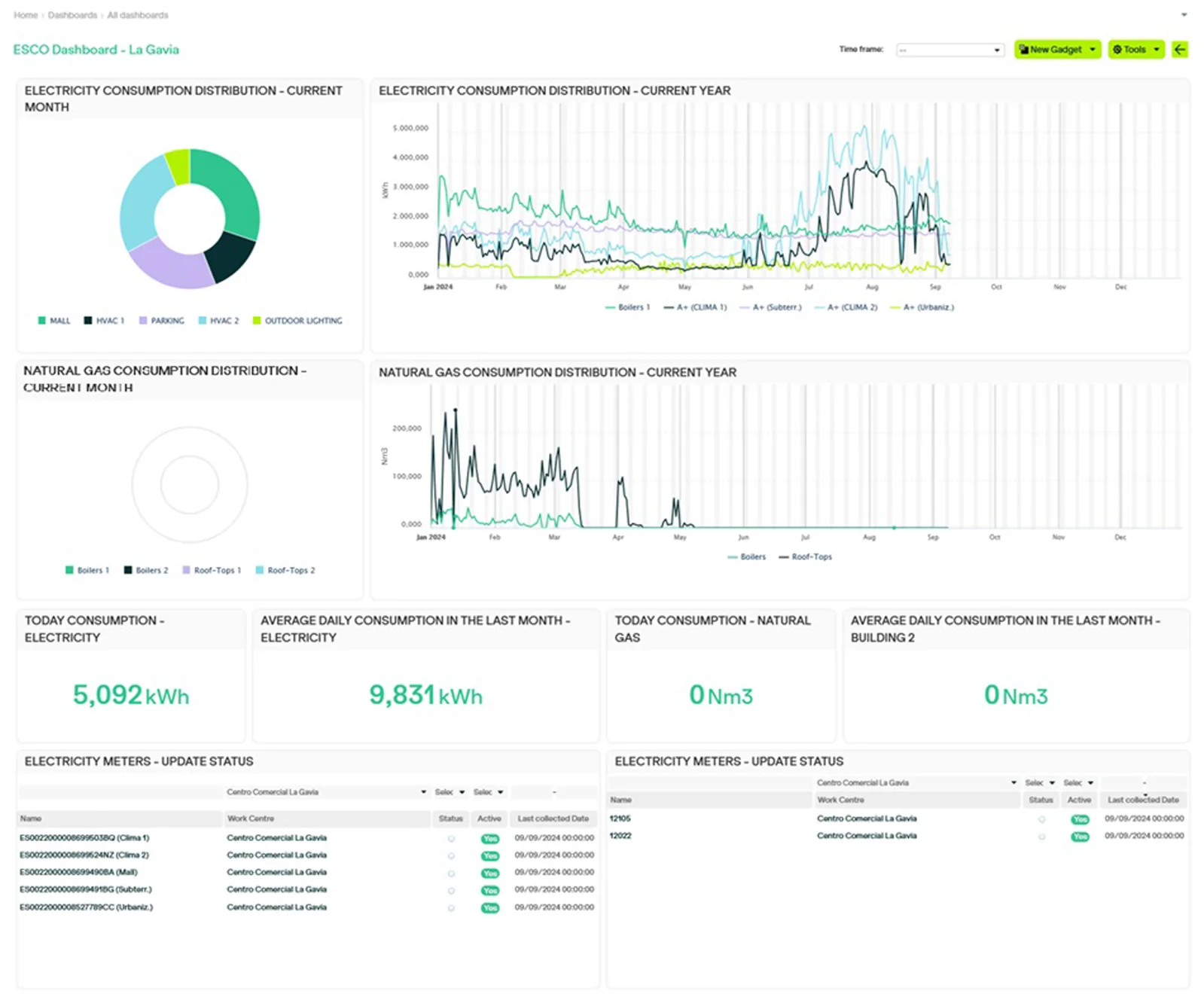
Visualisation dashboard developed by Smarkia in the H2020 SmartSPIN project.

**Figure 10.**
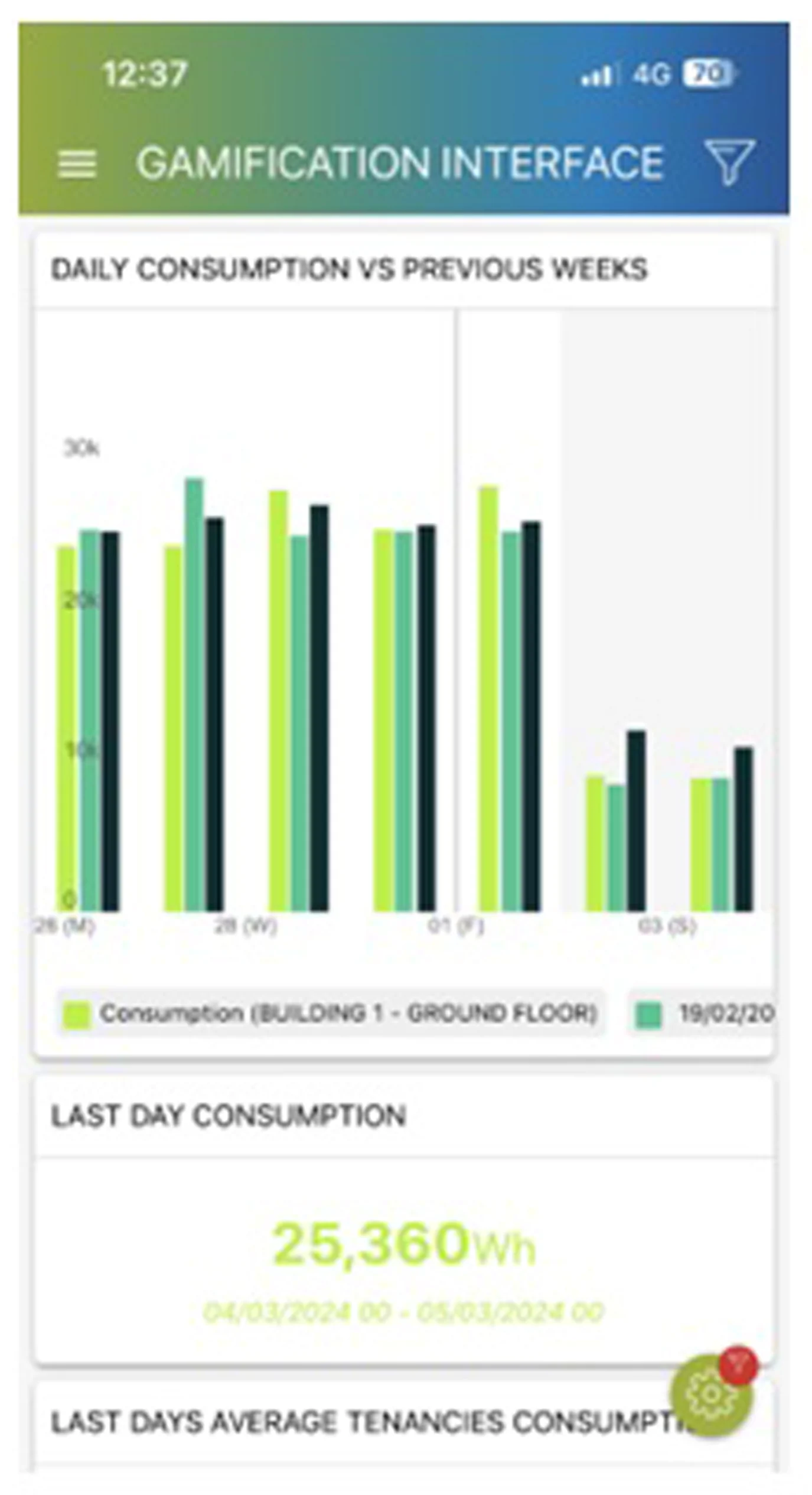
Gamification app developed by Smarkia in the H2020 SmartSPIN project.

*Value Proposition*

The proposed smart energy service delivers an Increased value and greener image of the property after installation of energy efficiency measures and BMS upgrades, a Renovated property and non-energy benefits such as increased indoor comfort (e.g. thermal comfort and air quality). The monthly service fee paid by the renters (and in some cases by the building owner as well) for the energy efficiency service deliver value to the ESCO that implements the service. The value proposition is an aggregation of benefits that ESCOs offer to customers. These benefits include energy savings/reduced energy bill, but also better thermal comfort, and in some cases improved indoor air quality, reduction of water waste, and other environmental benefits. The value of the service for the customers is determined by the responsibility taken by the ESCO for the installation, commissioning, monitoring and regular maintenance of equipment, devices and systems and the provision of guaranteed/shared energy savings to their clients. Products that typically create value across all the segments are heat pumps, Photo-voltaic panels, LED lighting, Building management systems, smart building controls, amongst the various measures. Sub-services relevant with the energy efficiency service are equipment replacement or installation, maintenance, measurement and verification of the energy savings. The value proposition of the business model in Table 1 is innovative and represents a new and disruptive offer, because the energy efficiency service is deployed through energy performance contracting, but with features that make it suitable for commercial rented buildings.

*Channels*

The communication, distribution and sales channels are the ESCO’s interface with the customers. The functions of the channels are to raise awareness about the offered products and services, to help customers to evaluate ESCO’s value proposition, to allow customers to purchase or lease specific products and services, to deliver a value proposition to customers and to provide a post-purchase customer support. The required communication and marketing channels are website and blog, printed posters displayed at strategic locations, printed or digital flyers or brochures, social media channels, printed or emailed newsletters, press releases, TV or radio advertisements, outreach at trade shows and sectoral events, outreach via building owners and managers associations, business referral.

*Cost Structure*

The costs can be calculated by the ESCO after defining key resources, key activities and key partnerships. The business model in Table 1 is cost-driven and it is built on a low-cost structure; a key to be successful should be to use only the cheapest resources that can deliver a certain amount of energy savings. The key activities which are expensive are likely to hinder the commercial viability of the service. More specifically, the costs to be considered are the purchase, installation and financing cost of energy efficient equipment, the operation and maintenance costs of installed equipment, the fees of consultants and contractors, the costs of ESCO personnel, the costs associated with the measurement and verification of energy savings.

*Revenue Streams*

The customers are willing to pay for an improved energy efficiency and some of them to reduce their environmental impact. Some customers are willing to pay for improving their indoor comfort (e.g. thermal comfort). The pricing mechanism for the service should be based on a transparent and trustable estimation of the achievable energy savings as well as on whether the ESCO guarantees a certain amount of savings to their customer or simply shares the savings according to fixed percentages. The main revenue stream for the ESCO delivering the service comes from the share of energy cost savings obtained by the clients, i.e., renters and building owner (the latter only if they occupy part of the building) and the revenue stream for the operation and maintenance service. An additional revenue stream may come from equipment rent, if the ESCO owns the equipment and rents it to the client for a fixed monthly fee. All the revenue streams are collected through a service fee.

***3.3.2 Value Proposition***

This section discusses the development of the value proposition for the smart energy service for the commercial rented sector proposed in this paper, applying the value proposition CANVAS previously introduced in Section 2.4. Result of this analysis are presented in Table 2.

**Table 2.** Value proposition of the proposed Business Model and its matching with customer-side requirements.

PRODUCT			CUSTOMER	
**Benefits** • Delivers energy efficiency gains and energy cost reductions to clients that occupy rented commercial properties. • Minimizes the performance risks for the client by providing an adequate level of guaranteed energy savings. • Provides a comprehensive service encompassing selection of energy efficiency measures, their installation, optimised system operation and maintenance, measurement and verification of energy savings. **Features** The Smart Energy Service will: 1. Design a building retrofit and building systems upgrades in collaboration with the client. 2. Implement, monitor and optimise selected energy efficiency measures. 3. Measure and verify the energy savings periodically (e.g. monthly or quarterly). 4. Provide a compensation to the clients if the contractual guaranteed energy savings are not achieved. 5. Reward the building owner proportionally to the savings achieved by the renters.	**Experience** ESCO’s clients gain certainty about the energy savings that will be achieved because the ESCO bears the performance risk of the project. Building owners are incentivised in taking the responsibility for an investment in energy efficiency because they are rewarded by the ESCO depending on the amount of savings achieved by the renters. The ESCO acts as a one-stop shop providing a comprehensive service, that does not require building owners and renters to liaise with multiple contractors. The ESCO takes the responsibility not only to install equipment and energy efficiency measures, but also to maintain them and operate them in an optimised manner.	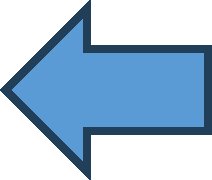	**Wants** Building owners want: 1) Renovate their buildings to increase their value on the rental market. 2) A revenue stream from an energy efficiency project. 3) Assistance with project financing, or identification of available grants that can support the purchase/installation of suitable energy efficiency measures. 4) Select and install affordable energy efficiency measures with a short payback time. 5) An energy performance contract (EPC) of not too long duration to maximise the rewards from the installed energy efficiency measures and mitigate the risk that some renters would leave, making the EPC no longer sustainable. Renters want: 1) Rent a renovated and comfortable property. 2) Increase their energy efficiency. **Needs** • A detailed *energy audit* to accurately evaluate the expected energy savings and the ability of the selected energy efficiency measures in achieving them. • To minimize the *technology risks* due to improper system design, improper equipment selection, unexpected equipment deterioration. • *Training* to operate the newly installed equipment according to the prescribed operation schedule and optimal control strategy and procedures. • A flexible *EPC contractual template* including clauses enabling 1) a system monitoring and optimisation period; 2) the building owner to obtain a reward proportional to the energy savings achieved by the renters.	**Fears** The energy efficiency measures are not affordable, or their payback time is too long. The expected energy savings will not be achieved for a variety of reasons such as their inaccurate estimation, low quality of system operation and maintenance, system performance degradation due to equipment wear and tear and operation parameter variations. Renters do not contribute to the energy efficiency investment, whereas the energy savings that they would achieve cannot be easily shared with the building owner, who takes the responsibility for the investment (split incentive issue).
**Product** Smart Energy Service for Commercial Rented Properties.	**Ideal customer** Building owners and renters of commercial rented units.		**Substitutes** The main substitute is the traditional method, also known as Design–bid–build or Design– tender, in which the building owner contracts with separate Companies and Consultants for the design and construction of an energy efficiency project.	

The proposed smart energy service delivers an Increased value and greener image of the property after installation of energy efficiency measures and BMS upgrades, a Renovated property and non-energy benefits such as increased indoor comfort (e.g. thermal comfort and air quality). The monthly service fee paid by the renters (and in some cases by the building owner as well) for the energy efficiency service deliver value to the ESCO that implements the service. These benefits include energy savings/reduced energy bill, but also better thermal comfort, and in some cases improved indoor air quality, reduction of water waste, and other environmental benefits. The value of the service for the customers is determined by the responsibility taken by the ESCO for the installation, commissioning, monitoring and regular maintenance of equipment, devices and systems and the provision of guaranteed/shared energy savings to their clients. Products that typically create value across all the segments are heat pumps, Photo-voltaic panels, LED lighting, Building management systems, smart building controls, amongst the various measures. Sub-services relevant with the energy efficiency service are equipment replacement or installation, maintenance, measurement and verification of the energy savings. The channels section describes how the ESCO communicates with and reaches its customer segments to deliver its value proposition.

**3.3.2.1 Product or service**

*Benefits*

The service delivers value consisting in energy efficiency gains and energy cost reductions to clients that occupy rented commercial properties. It minimizes the performance risks for them by providing a minimum level of guaranteed energy savings. Another benefit is the “one-stop shop approach” where a single contractor (the ESCO/energy efficiency provider) provides an integrated set of energy services to the customer, such as selection of energy efficiency measures, their installation, optimised system operation and maintenance, measurement and verification of energy savings.

*Features*

The features of the proposed smart energy service include the design of a building retrofit and building systems upgrades considering the client’s requirement, the implementation, monitoring and optimisation of the selected energy efficiency measures, the periodic measurement and verification of the energy savings achieved once that the measures/equipment are installed (e.g. monthly or quarterly). Other features are the provision of a compensation to the clients if the guaranteed energy savings agreed in the energy performance contract are not achieved, and the reward given to the building owner, that is proportional to the savings achieved by the renters. The latter is the key innovative feature of the proposed service, that enables to overcome the split incentive issue in the commercial rented sector ( De Tommasi *et al.*, 2024).

*Experience*

The customers of the proposed smart energy service will experience positive feelings due to the certainty about the energy savings that will be achieved, because the ESCO bears the performance risk of the project ( Lee *et al.*, 2015). Building owners will feel more motivated and confident in taking the responsibility for an investment in energy efficiency, because they know that will be rewarded by the ESCO proportionally to the amount of savings achieved by the renters. Building owners and renters will not need to spend much of their time liaising with multiple contractors, because the ESCO acts as a one-stop shop providing a comprehensive service. Indeed, the ESCO takes the responsibility for installing equipment and energy efficiency measures, as well as to maintain them and enable the clients to operate them in an optimised manner, such that the planned energy savings can be achieve.

**3.3.2.2 Customer**

*Wants*

The building owners want to renovate their buildings to increase their value on the rental market. They want a revenue stream from an energy efficiency project, because there would not be any incentive for them to invest in an energy efficiency project without such reward. They want assistance with project financing, including the identification of available grants that could support the purchase/installation of suitable energy efficiency measures. Building owners need the service of specialised companies to select and install affordable energy efficiency measures with a short payback time. They want an energy performance contract (EPC) of adequate and not too long duration to maximise the rewards from the installed energy efficiency measures and mitigate the risk that some renters would leave, making the EPC no longer sustainable. On the other hand, renters want a renovated and comfortable property, and increase their energy efficiency, if that’s an achievable objective.

*Needs*

In the context of energy efficiency in commercial rented buildings, customers need detailed energy audits to accurately identify energy efficiency measures that can achieve substantial energy savings and to estimate the amount of such savings. They need a service that can minimize the technology risks due to improper system design, improper equipment selection, unexpected equipment deterioration ( Lee *et al.*, 2015). They also need appropriate training to operate the newly installed equipment according to the optimal operation schedule and optimal control strategy and procedures identified by the ESCO. Furthermore, customers need a flexible EPC contractual template including clauses which enable to implement a system monitoring and optimisation period, and that provide the building owner with a reward proportional to the energy savings achieved by the renters ( De Tommasi *et al.*, 2024).

*Fears*

In the context of smart energy services suitable for the commercial rented sector, a possible fear of the customers is that the energy efficiency measures are not affordable, or their payback time is too long. Another common fear is that the expected energy savings will not be achieved for a variety of reasons such as their inaccurate estimation, low quality of system operation and maintenance, system performance degradation due to equipment wear and tear and operation parameter variations. Moreover, yet another fear arises from the fact that the commercial property is rented, that is the renters not contributing to the energy efficiency investment (because they do not own the premises they are renting), whereas the energy savings that they would achieve cannot be easily shared with the building owner, who takes the responsibility for the investment. The latter barrier is the split incentive issue previously discussed in Section 2.1 ( Castellazzi *et al.*, 2017; Ring *et al.*, August 2022).

## 4. Conclusions

This paper has used a quantitative model that enables the evaluation of the Net-Present-Value of an energy efficiency project for building owner, energy service company (service provider) and renters, to highlight how the energy cost savings, energy prices, investment costs, operation and maintenance costs and the rebound effect of the energy consumption of the renters, may influence the decision-making in relation to the feasibility of an energy performance contract in a rented commercial property, as well as the contract duration. The analysis showed that the higher the capital investment made by the ESCO, the longer the contract duration proposed by the ESCO. The decision making in relation to contractual agreement can be greatly facilitated if transparent calculations are shown to the potential customers, as shown in this paper. Furthermore, it has been highlighted that a suitable arrangement to overcome the barrier of the long duration of the Energy Performance Contracts (EPC) in the commercial rented sector is to foster the sharing of the initial capital investment in energy efficiency measures and equipment between ESCO and customer (building owner), such that the EPC contract duration can be reduced because of the customer’s direct contribution to the investment, while the ESCO would still contribute to it (when the client cannot fully bear the investment).

Moreover, the review presented in this paper shows that, when renters are not given sufficient economic incentive to save energy, they might use even more energy after the energy retrofit, reducing the total energy savings and the value of their part taken by the ESCO. A sensitivity analysis was performed with respect to the two parameters used to model the rebound effect (i.e., the risk tolerance and the maximum rebound effect) which showed the importance for an ESCO to estimate as accurately as possible such effect. Indeed, in presence of a high rebound effect, the energy savings will be lower than expected and the ESCO will need a longer contract duration to reduce the risk of economic losses and maximize the chances to achieve the desired profit.

The underlying business model for a smart energy service suitable for the commercial rented sector has been fully characterised and analysed using the business model CANVAS table. The following aspects of relevance for the businesses that want to implement the service: key partners, key activities, key resources, cost structure, value proposition, customer relationship, channels, customer segment, revenue streams, were highlighted. Furthermore, the paper has investigated how the considered smart energy service can create value for the customers, designing its value proposition using the Value Proposition CANVAS templates.

## Reporting guidelines

This paper provides all the details of the materials and methods used so that the work can be repeated by others. These details include: 1. equations used for the quantitative analysis of the business model and the related decision making undertaken by the stakeholders; 2. the CANVAS Business Model & Value Proposition templates that have been used for the design of the proposed business model; 3. A comprehensive list of references.

## Ethics and consent

Ethical approval and consent were not required.

## Data Availability

No data associated with this article.
