# A smart energy service for the commercial rented sector: decision making points and business model considering the learnings from the pilot sites of the SmartSPIN project in Ireland, Spain and Greece

**DOI:** 10.12688/openreseurope.18851.2

**Published:** 2025-12-16

**Authors:** Luciano De Tommasi, Ruchi Agrawal, Padraig Lyons

**Affiliations:** 1 International Energy Research Center, Tyndall National Institute, University College Cork, Cork, County Cork, T12R5CP, Ireland

**Keywords:** smart energy service, energy efficiency, commercial rented buildings, CANVAS business model

## Abstract

**Background:**

This paper presents an evaluation of a smart energy service for the commercial rented sector, highlighting the key decision-making points in relation to energy performance contracting. The work was developed during the European Commission’s Horizon 2020 funded research project SmartSPIN - Smart energy services to solve the SPlit INcentive problem in the commercial rented sector.

**Methods:**

The perspectives of building owner, energy service company (energy service provider) and renters, are examined using a quantitative model that enables to evaluate the Net-Present-Value of the energy efficiency project for each party, considering the contract duration, energy cost savings, energy prices, investment costs and operation and maintenance costs. The business model for the proposed smart energy service is designed using the Business Model CANVAS, whereas its value proposition is designed using the Value Proposition CANVAS.

**Results, research gap and originality of the proposed approach:**

The results of the paper contribute to the existing body of literature in two main ways. First, a quantitative model highlighting the key variables that determine the contractual arrangements and profitability of a performance contract for the parties is thoroughly analysed. Second, a business model for the proposed smart energy service is developed by applying the Business Model Canvas and the Value Proposition Canvas frameworks. This addresses a notable gap in the literature, as no prior studies have established a similar business model in the context of commercial rental properties using the proposed methodology.

**Conclusions:**

This paper shows how a smart energy service for improving the energy efficiency and flexibility in commercial rented buildings can be successfully designed and deployed, enabling to overcome the split incentive issue, which is the situation where landlords bear the cost of energy efficiency improvements, but tenants receive the financial benefits of reduced energy bills, discouraging investment.

## 1. Introduction

### 1.1 Problem statement and paper’s contribution

The commercial rented sector faces a split incentive problem, where landlords fund energy efficiency improvements while tenants benefit from reduced energy costs, limiting investment. Designing an effective smart energy service requires a business model that aligns the interests of all stakeholders. This study develops such a model using the Business Model Canvas and Value Proposition Canvas, with their design underpinned by a quantitative analysis of the net present value (NPV) for building owners, energy service companies (ESCOs), and tenants under different contractual arrangements, including contract duration, annual energy cost savings guarantees, the sharing of energy savings, and service fees paid to the ESCO. Insights from interactions and feedback at SmartSPIN pilot sites further refine the model, highlighting how it creates value for clients while offering clear benefits to ESCOs seeking to implement it.

### 1.2 Research background

Energy services are functions performed using energy to deliver desired outcomes, such as space and water heating and cooling, lighting, cooking, or refrigeration. ( Fell, 2017). Their contribution to human well-being can be studied using via the Energy Service Cascade, which links structures, functions, services, benefits, and values ( Kalt *et al*., 2019). *Structures* include energy conversion chain and infrastructures, functions are the physical actions of energy systems, services are the functions demanded by humans, benefits are the positive outcomes, values may be economic or non-economic. The economic value of an energy service is the willingness of humans to pay for it. Non-economic values include the habits and societal preferences that influence the demand for the service as well as the benefits that humans perceive from it.

Smart energy services are those ones that are based on smart technologies and/or innovative business models. Examples of smart energy services are energy supply and billing, monitoring and guidance, control and automation, energy trading, demand response, energy community services, smart home and smart metering set-up and support services, decentralized energy resources set-up and support services, integrated energy management ( Paukstadt, 2019). Integrated Energy Management Services rely on ICT and big data platforms for access to smart meter data, energy monitoring, prediction, optimisation, billing and brokerage supporting design and development of innovative business models for stakeholders such as ESCOs, energy managers, utilities, authorities and citizens ( Karnouskos *et al.*, 2013; Marinakis *et al*., 2020).

Smart energy services can be classified considering their positioning within the value chain: generation and storage (i.e., asset management services such as real-time condition monitoring, predictive maintenance, remote control of assets, performance optimisation), trading and power system balancing (e.g., demand response and flexibility services, energy communities and peer-to-peer energy trading), retail (i.e., energy supply and billing), consumption (i.e., energy monitoring and management) ( Paukstadt & Becker, 2021). Sustainable energy services also support smart grid development by balancing production, distribution, and consumption of renewable energy ( Borysiak & Brych, 2021). Despite technological progress, adoption barriers remain, including mistrust, lack of knowledge and limited resources, which can hinder market actors from designing and implementing smart energy services via advanced business models such as Energy Performance Contracting (EPC) ( Kahma & Matschoss, 2017; Manfren *et al.*, 2021). Information hubs, use of ICT technologies, marketing and information campaigns for consumer engagement, can help to reduce barriers on information ensuring that flows of information on dynamic tariffs, prices and services are clear for the market players and end consumers ( Shivakumar *et al.*, 2018).

A smart energy service is delivered through smart devices and a connectivity technology that enables customers to control equipment and appliances ( Gonçalves *et al.*, 2020). Moreover, information and communication technologies may be used to improve energy efficiency in smart buildings, fostering a behavioural change of the occupants determining a change of occupants’ daily energy consumption patterns. In fact, sensor data analysis and energy/behavioural analytics enable development of targeted recommendations, bespoke actions and decisions that determine a behavioural change of occupants and energy efficiency increase ( Fotopoulou *et al.*, 2017).

Smart energy services may integrate plug-in electric vehicles (PEV) charging through Vehicle-to-Grid (V2G) and Grid-to-Vehicle (G2V), reducing the carbon dioxide (CO _2_) emission and optimising grid interaction ( Anthony Jnr, 2021).

Co-creation with customers enhances energy management solutions and value, while new energy trading services, including power and carbon trading, rely on enabling technologies such as blockchain and risk management platforms ( Gonçalves *et al.*, 2020; Xie *et al.*, 2021).

Innovation in smart energy services must address both technical and business challenges, revising traditional business models to fit the emerging smart grid context ( Alvarez *et al.*, 2015).

Like traditional ESCO services, the smart energy service considered in this paper includes a variety of activities, such as energy analysis and audit, equipment supply, energy management, project design and implementation, monitoring and evaluation of energy savings, maintenance and provision of training for energy efficient system operation ( Bertoldi *et al.*, 2006). However, the proposed smart energy service builds on an innovative business model, which enables to make it suitable for deployment in commercial rented properties. Many commercial buildings in Europe show high potential for retrofitting with short payback time with Energy Performance Contracting ( Garrido-Marijuan *et al.*, 2024).

### 1.3 Objective, contributions and structure of the paper

The objective of this paper is to analyse the factors influencing the decision-making process which determine the contractual arrangements in an Energy Performance Contract for commercial rented buildings and to establish a business model for a performance-based Smart Energy Service (SES) to be implemented by an Energy Service Company (ESCO) that wishes to make the proposed SES commercially available.

The contributions of the paper consist in: 1. Using a financial model based on the calculation of the Net-Present-Value for the building owner, tenants and Energy Service Company (ESCO) to analyse the perspective of each Party when negotiating the terms of an EPC; 2. Using the Business Model Canvas and the Value Proposition Canvas templates to work out all the practical implementation aspects for the proposed business model.

The remaining part of this paper is structured as follows. Section 2 introduces the research context. Section 2.1 discusses data collection. Section 2.2 presents an analysis of the barriers to the deployment of smart energy services in commercial rented buildings (i.e. the split incentive issue and the long contract duration) and the proposed solution, which is a tri-partite energy performance contracting business model. Section 3 focuses on the Methodology to implement the proposed smart energy service. Section 3.1 describes the high-level methodology and the steps to be followed for implementing the proposed smart energy service in a commercial rented property. Section 3.2 introduces the mathematical model used to analyse the factors influencing the decision-making process in relation to Energy Performance contracting for commercial rented buildings. Section 3.2.1 presents the Net Present Value Calculations and decision-making processes for ESCO, building owner and renters. Section 3.2.2 presents the modelling of energy savings; Section 3.2.3 the modelling of energy cost savings, Section 3.2.4 the modelling of operation and maintenance costs. Section 3.3 and Section 3.4 introduce respectively the Business model CANVAS, and the value proposition CANVAS. Section 4 reports the main results of the paper. Section 4.1 introduces the case study developed. A discussion of the factors influencing the decision-making process in relation to Energy Performance contracting for commercial rented buildings are discussed in Section 4.2 ( Section 4.2.1, Section 4.2.2 and Section 4.2.3 develop the analysis for the efficiency provider, building owner and renters respectively). Section 4.3 discusses the factors that determine the contract duration. Section 4.4 discusses the practical implementation of the business model for the proposed smart energy service for the commercial rented sector, using the Business Model Canvas and the Value Proposition Canvas templates (respectively Subsection 4.4.1 and Subsection 4.4.2). Section 5 summarises the main learnings from the pilot-sites of the SmartSPIN project in Ireland, Spain and Greece. Section 6 concludes the paper.

## 2. Research context

### 2.1 Data collection

This paper builds on the analysis conducted by the authors in ( De Tommasi *et al.*, 2024), which compared eight business models for Energy Performance Contracting applicable to the European commercial rented sector. The research team engaged stakeholders of the commercial rented sector through ten interviews and a focus group to determine, respectively, the high-level requirements for a smart energy service suitable for commercial rented properties and the main features of the business model required to deliver the smart energy service (in addition to provide comments about the progress of the SmartSPIN project). The interviews were held between September 2021 and August 2022. The stakeholders involved are listed in ( Agrawal & De Tommasi, 2022). Two focus group meetings were held on 7 ^th^ September 2022 and 29 ^th^ August 2023. The stakeholders who participated in the interviews were invited to participate in the focus group.

### 2.2 Analysis of barriers to the deployment of smart energy services in commercial rented buildings and a possible solution

Once that the factors determining energy efficiency in a commercial building (such as weather, occupancy, building systems design, building management system, energy management) are well understood ( Ismail & Inambao, 2019), several opportunities for energy efficiency improvement projects and measures can be easily identified ( Garrido-Marijuan *et al.*, 2024). However, their possible implementation using performance-based service models such as energy performance contracts (EPC) has been hindered by the complexity of such type of contract, and more specifically by barriers like the long contract duration of EPCs and the split incentive issue. In many cases, the contract duration of an EPCs is between 8 and 15 years, which may often be longer than the tenancy contract. A split incentive issue occurs when, e.g., in a rented building, the occupiers pay the energy bills, and the building owner is deterred to invest in energy efficiency measures because the benefit of reduced energy bills would accrue only to the buildings’ occupiers. The SmartSPIN project has developed an innovative business model based on the Energy Efficiency-as-a-Service (EEaaS) concept, which can effectively remove the barriers that hinder the adoption of the performance-based models in the commercial rented sector and validated this approach in four pilot buildings in Spain, Greece, and Ireland.

The SmartSPIN business model combines an energy efficiency service delivered by an energy efficiency provider (energy service company) through the installation and maintenance of energy efficiency measures (such as optimization of heating & cooling schedules, upgrades to lighting systems, upgrade or replacement of heating, cooling, ventilation and air conditioning (HVAC) equipment, building envelope improvement) with a demand side flexibility service, which enables to shift flexible consumption to the hours of the day where the electricity is cheaper ( Ring *et al.*, October 2022). A new flexible tariff model has been developed ( Ring *et al.*, 2023). This model incentivises the shifting of the energy consumption from peak hours to non-peak hours of the day through a convenient tariff, which is applied if the energy consumers meet a certain level of energy flexibility. If the contractual level of flexibility is not met by the energy consumer, then a penalty is applied by the electricity supplier, resulting in a higher and less convenient cost for the consumed electricity. The SmartSPIN service delivers both energy and non-energy benefits to the stakeholders. The business model aims to split the value of the energy savings between all stakeholders (building owner, tenants and energy efficiency service provider) in a fair and transparent manner. This business model is implemented through two contractual templates, one for the energy efficiency service and another one for the flexible tariff ( Ring *et al.*, 2023).

A key role in delivering the energy efficiency service is played by the energy efficiency service provider. The energy efficiency provider (which is in most cases an energy service company) signs an energy performance contract with the building owner and the building occupiers and is in charge of installing and maintaining energy efficiency measures, measuring and verifying the energy savings achieved monthly (with respect to a previously agreed baseline) and bills the tenants (building occupiers) with a fee that represents the cost of implementing the bespoke energy efficiency service. The payment performed by the tenants to the energy efficiency provider enables to share part of the energy cost savings with the building owner, thereby incentivizing the investment in energy efficiency measures and solving the split incentive issue. In absence of the energy efficiency provider, the building owner might recover part of the costs of the retrofit through a service charge (or part of it) payable by the renters ( Dixon, 2014).

The energy service company (ESCO) delivers the energy efficiency service with the guaranteed savings formula, which means that when the measured savings are lower than the contractual agreed savings, they will compensate their clients with the value of the energy savings shortfall, this way bearing the performance risk of the project ( De Tommasi *et al.*, 2024; Guo & Zhang, 2020; Shang *et al.*, 2017). The cost of the energy efficiency service for the tenants and the level of guaranteed savings for the building owner is determined by the parties’ bargaining.

Figure 1 depicts SmartSPIN business model involving a tri-partite agreement between building owner, tenants and ESCO, where the building owner is financing the implementation of proposed energy efficiency measures through their own sinking fund, while the ESCO is delivering guaranteed savings to their clients and is billing the tenants for the costs of the service. The revenue stream determined by the tenants’ payments is used to reward the building owner, that otherwise would not be incentivised in investing in energy efficiency measures, because the benefits of such investment, i.e., a lower energy bill, would be reaped by the tenants only (we assume, for simplicity, that the building owner does not occupy any unit in the building). Detailed recommendations for overcoming the split incentive issue were identified in ( Castellazzi *et al.*, 2017; Ring *et al.*, August 2022). Figure 1 also highlights the possible rebound effect, which consists in an increased energy consumption by the tenants if they are not rewarded with part of the energy savings obtained. This paper aims to raise awareness of building owners and ESCOs in relation to such rebound effect, as if not considered during the decision-making process that leads to a performance contract signature, they could realise too late that energy savings do not meet the expectations, because a change in the energy consumption patterns of building renters has occurred ( Berkhout *et al.*, 2000; Sorrell *et al.*, 2009). It is worth noting that the contract types which have the potential to resolve the rebound effect directly have more limited applicability with respect to EPC. The first type of performance-based contract which can directly eliminate the rebound effect references post-project technology implementation rather than the client’s energy consumption ( Ding *et al.*, 2017). This contract offers lower guarantees of performances to ESCOs’ clients with respect to EPC and it is therefore less attractive for potential clients. The second type of contract that eliminates the rebound effect is the Chauffage, where ESCO takes the complete responsibility of the energy services delivered to its client, e.g., space heating and lighting, including the full responsibility of purchasing fuel and electricity as needed ( Bhattacharjee *et al.*, 2010). With the Chauffage contract the ESCO will also control the client’s comfort level, whereas energy consumption is determined by the comfort level agreed in the contract ( Ding *et al.*, 2017). The main weakness of the Chauffage is that the customer is less incentivised to save energy with respect to other contractual models including a shared savings agreement for some parts of the energy savings ( Wargert, 2011), like the one described in Section 2.2 of this paper. Moreover, Chauffage contracts are typically very long (20–30 years) and therefore not suitable for the commercial rented sector ( Bhattacharjee *et al.*, 2010).

**Figure 1.**
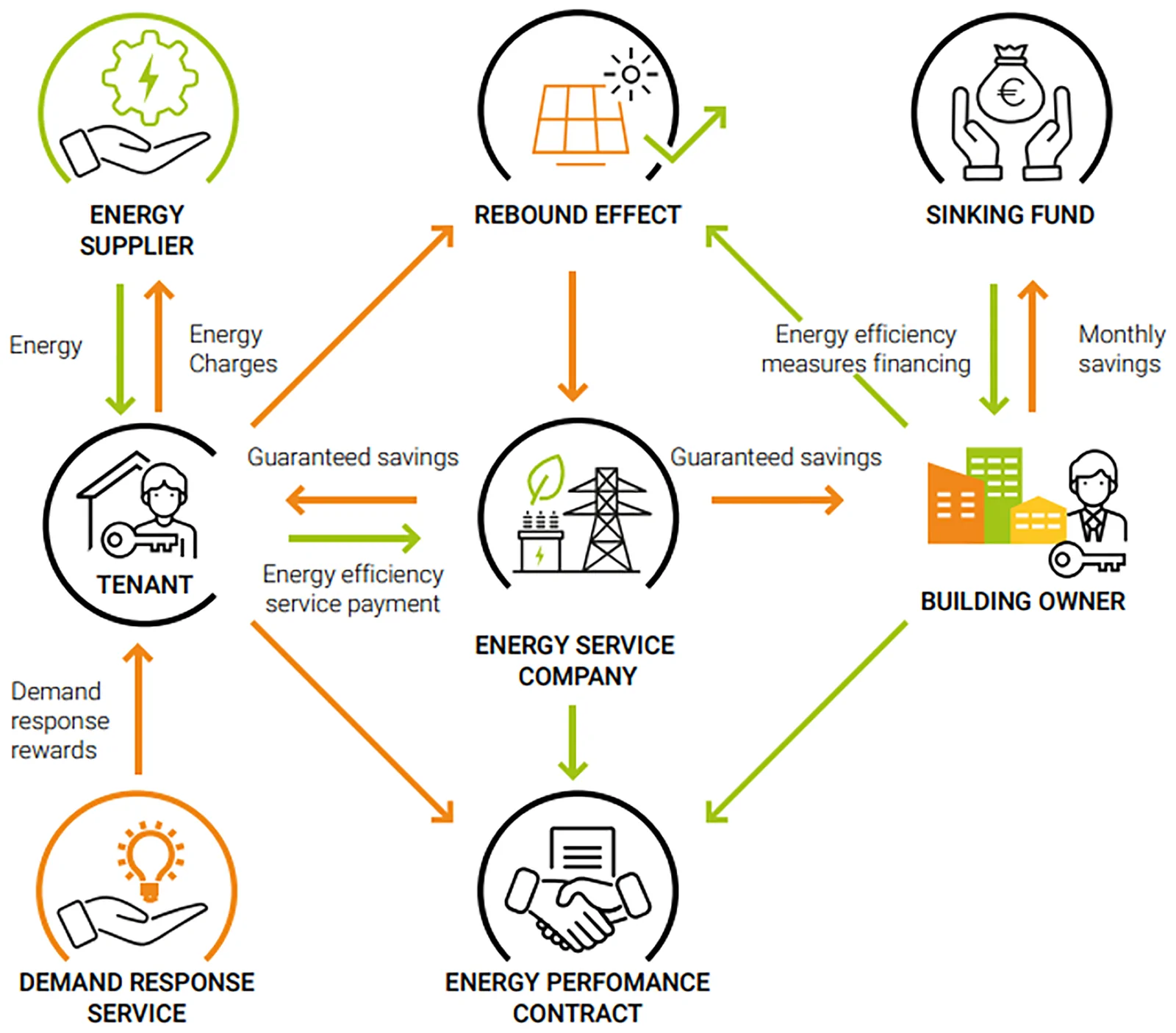
SmartSPIN Business Model – Energy efficiency measures financed by building owner through their own sinking fund.

In another variation of the business model depicted in Figure 1, the building owner funds the energy efficiency services by a taking loan from bank or any third party and the full responsibility for the loan repayment. Moreover, the energy efficiency service providers/ESCOs could fund the proposed upgrades taking the responsibility of a loan ( Figure 2). Another difference between the business models illustrated in Figure 1 and Figure 2, is that when the ESCO does not finance the EPC project, it will normally guarantee a certain level of energy savings to its client (guaranteed savings model, Figure 1), whereas when the ESCO finances the EPC project, it will share the energy savings with its client, taking a fixed portion of them over a fixed period (shared savings model, Figure 2).

**Figure 2.**
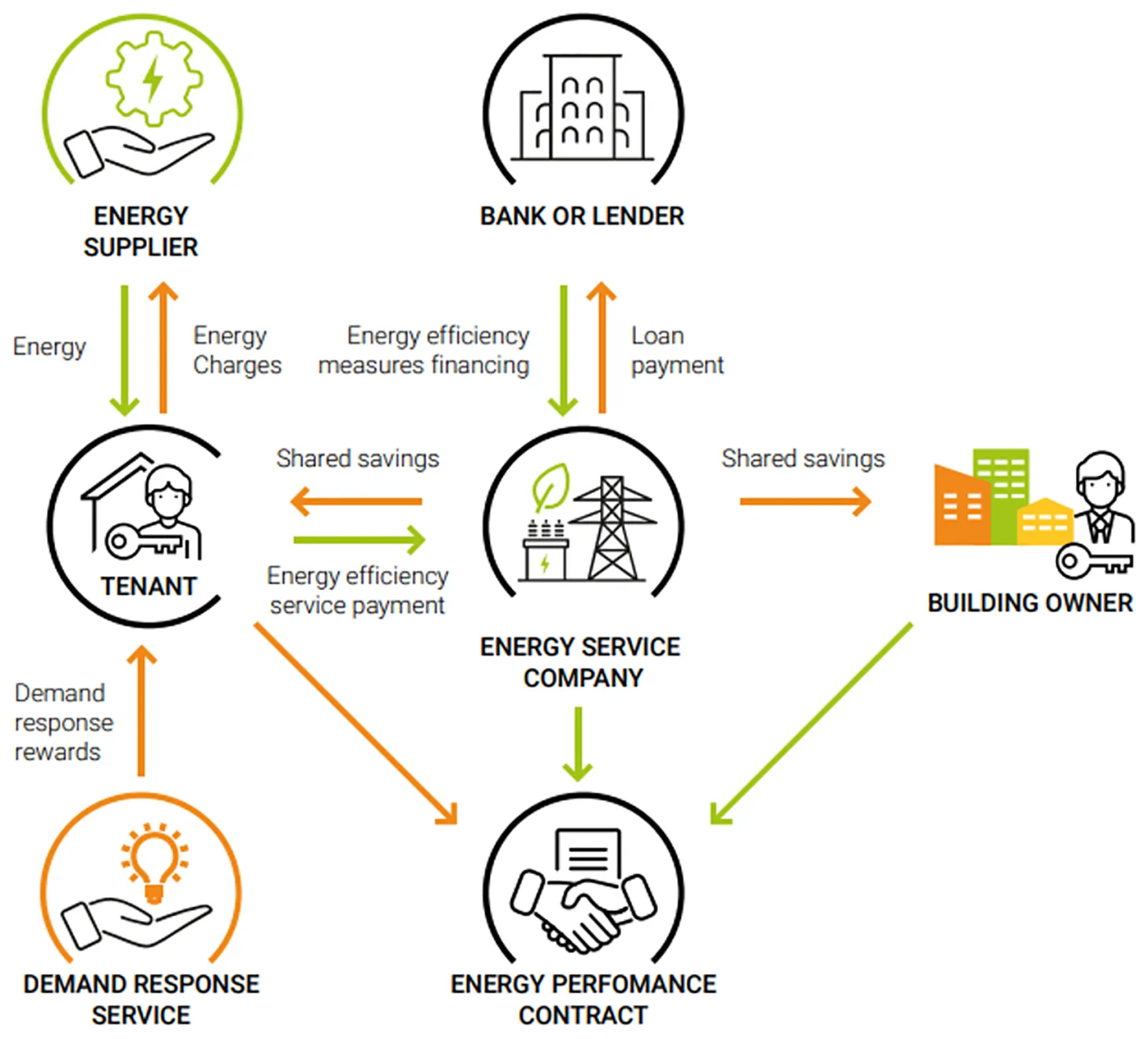
SmartSPIN Business Model – Energy efficiency measures financed by Energy Service Company through a loan arranged with Bank or Lender.

The implementation of the energy efficiency measures can be conveniently scheduled in two different stages (called implementation period/guarantee period) as depicted in Figure 3 (see also Agrawal *et al.*, 2022). In the first implementation or guarantee period, the energy efficiency service provider/ESCO engages with tenants and implements no-cost/low-cost measures. These measures include all the basic actions that can improve energy efficiency in commercial buildings in operation, which normally do not require permission from the building owner ( Escrivá-Escrivá, 2011). The first implementation period aims to demonstrate how the service works and to build the trust of the clients in the ESCO, while a decision to install medium/high-cost measures is taken by the building owner. During the second implementation/guarantee period, medium and/or high-cost measures (which require permission and a capital investment) are installed. The second implementation period aims to obtain high energy savings and reduce greenhouse gas ( Kneifel, 2010; Liang *et al.*, 2022), while reducing the performance risk for the building owner.

**Figure 3.**
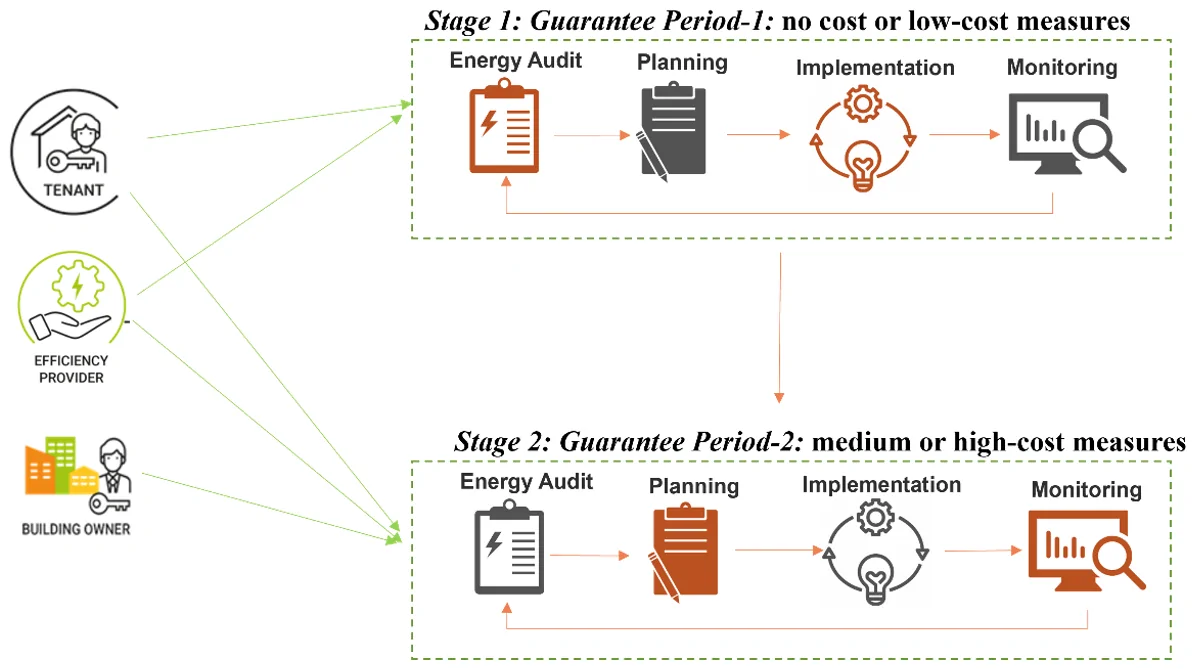
Implementation of SmartSPIN Smart Energy Service in two stages.

The steps followed for deploying the energy efficiency measures begin with an **energy audit**, to analyse energy use in detail and understand the amount of energy consumed across different systems or processes and the identification of areas of inefficiency. The audit provides a baseline and identifies potential opportunities for improvement. Once the audit is complete, the next step is **planning**. The purpose of planning is to decide which measures should be implemented based on their technical feasibility, financial viability, and expected impact. It involves setting priorities, defining targets, estimating costs and savings, and preparing an action plan with clear responsibilities and timelines. Following the planning, the **implementation** phase involves the practical execution of the selected measures. This involves installing new technologies, upgrading equipment, optimizing processes, or training staff. Finally, **monitoring** involves systematically tracking energy consumption and assessing it against the targets established during the planning phase. Any discrepancies are analysed, and corrective actions are implemented to sustain efficiency. Moreover, this activity generates valuable insights and lessons learned that inform future audits and projects, thereby contributing to establish a cycle of continuous improvement.

## 3. Methodology

### 3.1 High-level methodology to implement the proposed smart energy service

This section describes the high-level methodology to be followed by an ESCO that wants to implement the proposed smart energy service in a commercial rented property. The steps to be followed are illustrated in Figure 4 and explained in the following paragraph.

**Figure 4.**
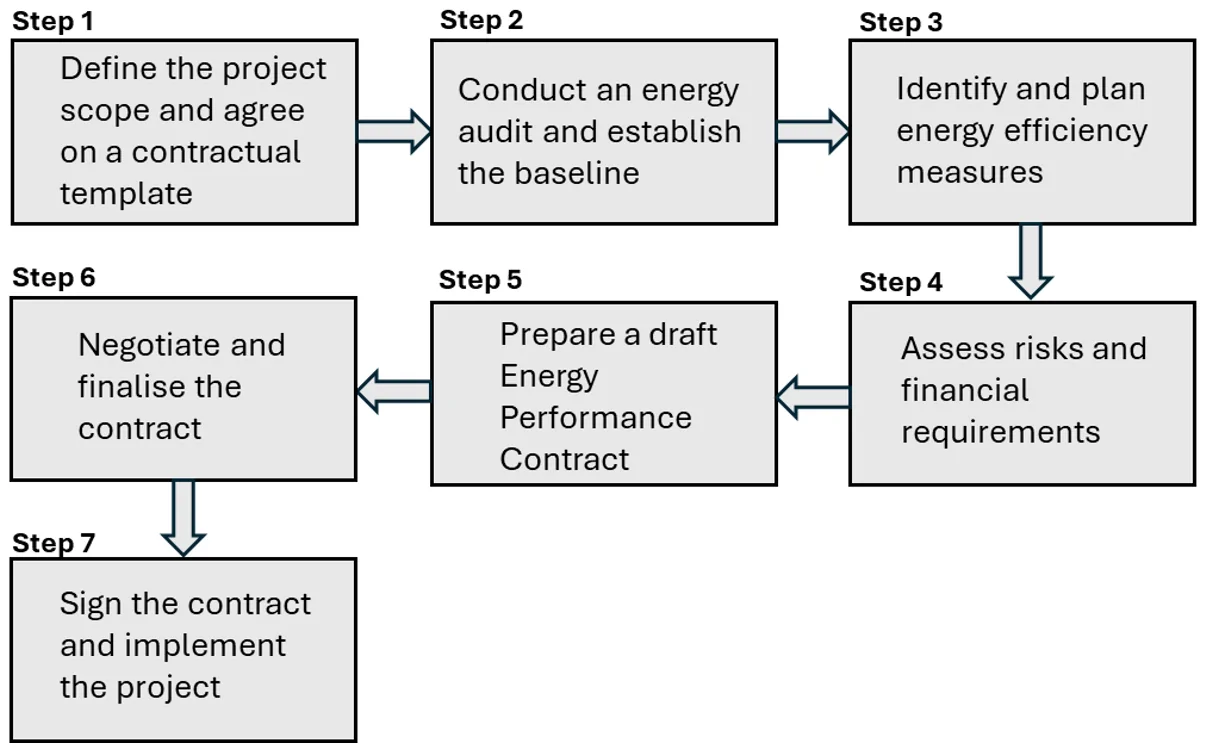
Steps for Implementing the proposed smart energy service in a commercial rented property.

The **first step** is to define the project scope and agree on a contractual template. In this step, the ESCO, building owner, and tenants identify the property and its energy profile, agree on the terms of the smart energy service, and establish a governance framework. This framework clearly specifies the responsibilities of each party and determines how energy savings will be measured and allocated. The **second step** is to conduct an energy audit, whereas the **third step** is to identify and plan energy efficiency measures (see Section 2.2 and Figure 3). The **fourth step** is to assess risks and determine financial requirements. The ESCO evaluates performance risks and sets guaranteed savings levels. This step also defines investment responsibilities for the ESCO, building owner, and tenants, and ensures that the project is financially viable for all parties involved. Following the risk assessment, a draft Energy Performance Contract (EPC) is prepared ( **fifth step**). This contract includes the scope of the project, the timeline for implementing the energy efficiency measures. the guaranteed performance targets, payment mechanisms, monitoring and verification protocols, and dispute resolution procedures. The draft serves as a starting point for negotiation among all stakeholders. The **sixth step** involves negotiating and finalising the contract. The building owner and tenants review the draft EPC, considering the implications for lease arrangements, cost-sharing, and benefit allocation. Revisions are made until all parties reach mutual agreement on the terms and conditions of the EPC. Finally, the contract is signed, and the project is implemented. This high-level methodology provides a clear and comprehensive roadmap for ESCOs to deliver smart energy services in the commercial rented sector, ensuring that energy efficiency, cost savings, and stakeholder engagement are systematically addressed throughout the project lifecycle. In the next Section 3.2, the suggested calculations supporting the financial assessment of the EPC and the decision-making process for its finalisation, will be described. Furthermore, Section 3.3 and Section 3.4 present the Business Model Canvas and the Value Proposition Canvas, respectively. These canvases provide the conceptual framework used to characterize the business model that an ESCO should adopt in order to offer the proposed smart energy service on a commercial basis.

### 3.2 Mathematical model used to analyse the factors influencing the decision-making process in relation to Energy Performance contracting for commercial rented buildings

***3.2.1 Net Present Value Calculations and decision-making processes***

The most important factors that determine the decision-making process in relation to Energy Performance Contracting for commercial rented sector are: 1. the investments required for the energy efficiency measures and the party that bears it (typically, either the building owner or the ESCO); 2. the envisaged (monthly) energy savings; 3. the cost of the service for the end users (i.e., the service fee paid by the tenants); 4. The division of the energy savings achieved between building owner, tenants and energy efficiency provider; 5. The duration of the energy performance contract; 6. Other aspects such as maintenance costs, energy prices and degradation of equipment performance due to aging.

A simulation-based decision model for designing contract period duration in energy performance contracting was proposed in ( Deng *et al.*, 2014). The simulation model accounts for Energy efficiency investment (capital and O&M) as well as energy cost savings (which depend on the energy savings amounts and on the energy prices) and assumes that the variations of energy savings and energy prices over time can be modelled with a Geometric Brownian Motion (GBM) process ( Feng *et al.*, 2022). We also do such assumption in this paper. One limitation of the study ( Deng *et al.*, 2014) that we overcome in this paper is that the rented scenario is not considered; hence, only the variations of the Net-Present-Value for the building owner and the ESCO are considered, without accounting for the possibility of including renters and their perspective on the EPC.

Lu, Zhang and Chen proposed an optimization model that determined the contract duration such that the *NPV _0_* (Net Present Value) of the investment in energy efficiency for the building owner is maximised under the constraint that the *NPV _E_* for the ESCO is greater than zero ( Lu *et al.*, 2017). They assumed that the ESCO is the party performing the investment and guaranteeing a certain level of energy savings to the building owner and tenants; the energy savings are shared between them according to the proportion which maximise the landlord’s reward considering the rebound effect for the tenants.

We argue that if the ESCO is performing the whole initial capital investment in energy efficiency measures the contract duration will be normally long, and that may hinder the actual deployment of the energy performance contract due to the risk that tenants (who pay the service fee to the energy efficiency provider, thereby enabling the EPC to run) would leave the property before end of the EPC. To mitigate such risk, the investment could be shared between the building owner and the ESCO in a proportion that is acceptable for the building owner and that allows the ESCO to achieve their targeted profit with a shorter contractual duration. Considering the revenues for the ESCO, renters and building owner respectively given in Equation (1), Equation (2) and Equation (3), the NPVs for ESCO, renters and owner are provided in ( 4), ( 5) and ( 6). *I _OM_* ( *t*) in ( 4) is the cost of the operation and maintenance services. The main difference between the model proposed in this paper and the one in ( Lu *et al.*, 2017) is the fact that the investment in energy efficiency measures performed at *t* = 0 is shared between the ESCO and the building owner according to a factor *γ*, such that the building owner invests *γ ^I^_c_* whereas the ESCO invests (1- *γ*) *I _c_* (these investments are negative cash flows at *t* = 0 for the calculation of NPVs). When *γ* = 1 the building owner is bearing the full investment in energy efficiency measures, whereas when *γ* = 1 the ESCO bears the full investment, which is the case considered in ( Lu *et al.*, 2017). The duration of the contract *n* that appears in ( 4) corresponding to a small *γ* is greater than the duration corresponding to a *γ* large because the positive cash flows must be accumulated to compensate a higher negative cash flow at *t* = 0.

$$R_{E}=\{\begin{array}{c} 0,\;t=0\\ R(t)-G-\text{max}[0,\beta (R(t)-G)],\;t=1,\ldots n\\ \;0,\;t=n+1,\ldots .N \end{array}\;(1)$$

$$R_{R}=\{\begin{array}{c} \;\;0,\;t=0\\ (1-\theta )(G+\max[0,\beta (R(t)-G)]),\;t=1,\ldots n\\ \;(1-\theta )R(t),\;t=n+1,\ldots \;.\;N \end{array}\;(2)$$

$$R_{O}=\{\begin{array}{c} \;\;0,\;t=0\\ \theta (G+\max[0,\beta (R(t)-G)]),\;t=1,\ldots n\\ \;\theta R(t),\;t=n+1,\ldots \;.\;N \end{array}\;(3)$$

$$NPV_{E}=(\gamma -1)I_{C}+\sum_{t=1}^{n}\frac{R(t)\text{−}G\text{−}max[0,\beta (R(t)\text{−}G)]\text{−}I_{OM}(t)}{{\left(1+r_{E}\right)}^{t}}\;(4)$$

$$NPV_{O}=-\gamma I_{C}+\sum_{t=1}^{n}\frac{\theta (G+max[0,\beta (R(t)\text{−}G)])}{{\left(1+r_{O}\right)}^{t}}+\sum_{t=n+1}^{N}\frac{\theta R(t)\text{−}I_{OM}(t)}{{\left(1+r_{O}\right)}^{t}}\;(5)$$

$$NPV_{R}=\sum_{t=1}^{n}\frac{\left(1\text{−}\theta )(G+\text{max}[0,\beta (R(t)\text{−}G)]\right)}{{\left(1\text{+}r_{R}\right)}^{t}}+\sum_{t=n+1}^{N}\frac{\left(1\text{−}\theta )R(t\right)}{{\left(1\text{+}r_{R}\right)}^{t}}\;(6)$$

The most appropriate contract duration *n* is the one which maximises *NPV _0_*. *NPV _0_* is maximised when both the share of the initial investment in energy efficiency measures *γ* for the building owner and the contract duration *n* are minimised. However, reducing *γ* has the effect of increasing the amount of the investment which must be performed by the ESCO and therefore increases the contract duration. The selection of the best contract duration and investment share between the landlord and ESCO can be formulated as in Equation (7) subject to the constraints ( 8) and ( 9). Compared to the approach in ( Lu *et al.*, 2017) the optimisation approach introduces an additional variable, the share of the investment *γ* that is performed by the building owner. The constraint ( 8) states that the investment performed by the building owner is lower or equal to the maximum feasible investment for the building owner *I _max,C_*. The constraints ( 9) states that the NPV for the ESCO must be greater or equal to the minimum acceptable NPV for the ESCO.

$$n_{opt},\gamma_{opt}=arg\;\max_{n,\gamma }NPV_{O}\;(7)$$

subject to:

$$\gamma I_{C}\leq I_{max,C}\;(8)$$

$$NPV_{E}\geq NPV_{E,min}\;(9)$$

It is remarked that the formulation in ( Lu *et al.*, 2017), which assumes *γ* = 0, may result in a too long contract duration, which may make the EPC infeasible in practice due to the shorter duration of the rental contracts of tenants and the very high risk of not finding timely new renters who could pay the service fee to the ESCO to enable them to continue running the EPC. The optimisation process will increase *γ* and try to reduce *n* while satisfying both constraints ( 8– 9).

***3.2.2. Modelling of energy savings***

The actual savings *Q*( *t*) achieved by the energy efficiency project can be expressed as follows:

Q(t)=f(t)⋅Re(θ)⋅K(t)(10)

where *f*( *t*) is the equipment degradation function (which decreases from 1 to 0 during the project lifetime), *K*( *t*) represents the energy savings potential, *Re*( *θ*) is the rebound effect multiplier, *θ* is the share of the energy savings kept by the building owner. It is assumed that the degradation function decreases to zero according to the following expression ( Lu *et al.*, 2017):

$$f(t)=\frac{\text{log}(N+1-t)}{\text{log}\;(N)}\;\;\;\;\;\;\text{t}=1,\;\ldots \;\text{N}\;(11)$$

where t is the elapsed time in years and N is the energy efficiency measures lifetime. The energy savings potential *K*( *t*) is modelled using a Geometric Brownian Motion (GBM) process. The average output of the GBM process can be approximated with the following expression:

$$K(t)=I_{C}K_{0}e^{-\frac{\sigma_{K}^{2}}{2}t+\sigma_{K}\varepsilon_{K}\surd t}\;(12)$$

The accuracy of the approximation ( 12) has been validated comparing it against the outputs of an actual Geometric Brownian Motion stochastic process ( QUANTPY, 2024), using parameters included in the case study in Table 1. Thirty Monte Carlo runs were performed, obtaining a mean Root Relative Square Error (RRSE) of 0.10986 with a standard deviation of 0.05672, which proves the accuracy of the approximation.

The rebound effect multiplier has the following form:

$$Re(\theta )=\alpha_{2}+\alpha_{1}e^{-\frac{k_{2}\theta +k_{1}}{\rho }}\;(13)$$

where $\alpha_{1}=\frac{\phi }{1-e^{k_{1}/\rho }}$ and *α _2_* = 1 – *α _1_*. Alternative forms for the rebound effect multiplier ( 13) are discussed and compared in the appendix. The rebound effect is maximum when the building owner does not share any savings with the renters ( *θ* = 1). The ESCO’s decision of delivering the service and the duration of the performance contract agreed with its client depend on the actual energy savings *Q*( *t*) throughout the duration of the project, which in turn depend on the rebound effect *Re*( *θ*). With the model ( 13), the behaviours of the renters are modelled through the two parameters *φ* and *ρ*, which are respectively the maximum rebound effect (corresponding to *θ* = 1) and the risk tolerance, which accounts for the individual risk attitudes of different renters. The rebound effect multiplier *Re*( *θ*) is plotted out considering different values of *φ* and *ρ* in Figure 5 (assuming and *k*_1_ = –100 and *k*_2_ = 200).

**Figure 5.**
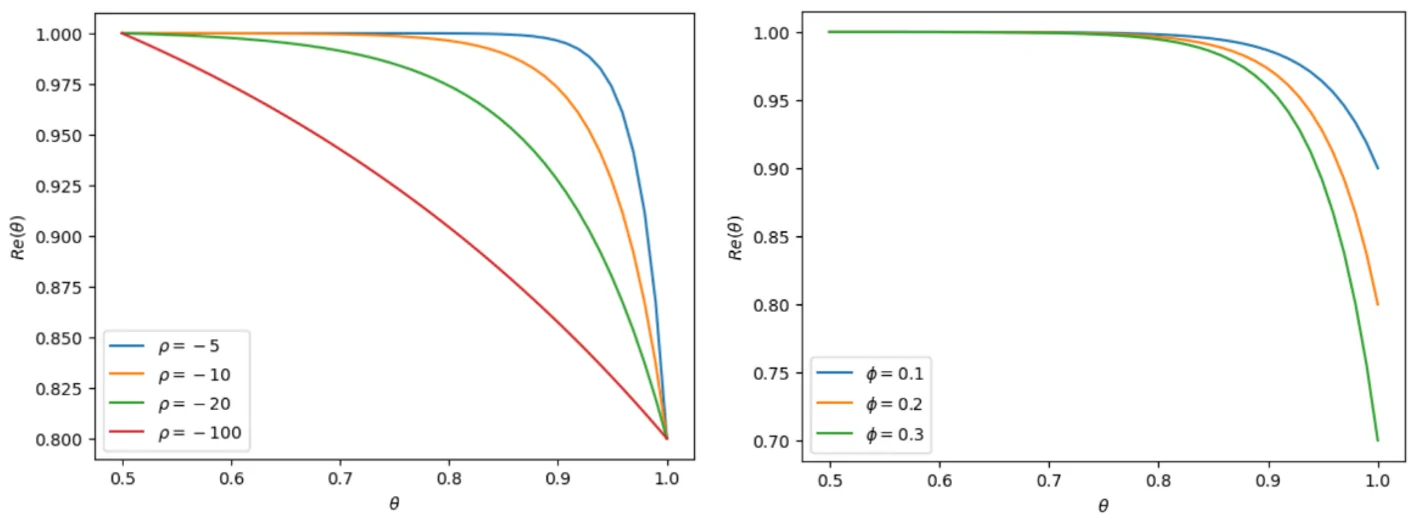
Variations of the rebound effect multiplier with *ρ* and *φ* parameters (respectively left-hand side figure and right-hand side figure).

It can be observed that when the percentage of the energy savings kept by the building owner *θ* increases, the rebound effect multiplier *Re*( *θ*) decreases lowering the *Q*( *t*); this effect may influence the decision of the ESCO to deliver the service as well as the minimum contract duration acceptable for them, which would fulfil the condition ( 9). An increment in *θ* determines a decrement in *NPV _E_* (due to the lower energy savings) for a given contract duration. Posing in *NPV _E,min_* = 0 in ( 9), we may determine the maximum percentage of energy savings *θ _max_* which the building owner can keep while ensuring that the ESCO may deliver the service (i.e., *NPV _E_* does not become negative).

***3.2.3 Modelling of energy cost savings***

An estimation $\tilde{R}(t)$ of the actual energy cost savings *R*( *t*) can be obtained as follows:

$$\tilde{R}(t)=Q(t)\cdot P_{E}(t)\;(14)$$

where *P _E_* ( *t*) is the future energy price, which is modelled through a Gaussian Brownian Motion (GBM) stochastic model. The average output of the GBM process can be approximated with the following expression ( Lu *et al.*, 2017):

$$P_{E}(t)=P_{E0}e^{\left(\alpha_{E}-\frac{\sigma_{E}^{2}}{2}\right)t+\sigma_{E}\varepsilon_{P}\surd t}\;(15)$$

The accuracy of the approximation ( 15) has been validated comparing it against the outputs of an actual Geometric Brownian Motion stochastic process ( QuantPy, 2024), using parameters included in the case study in Table 1. Thirty Monte Carlo runs were performed, obtaining a mean Root Relative Square Error (RRSE) of 0.01467 with a standard deviation of 0.00862, which prove the accuracy of the approximation.

***3.2.4. Modelling of operation and maintenance costs***

The operation and maintenance costs can be modelled as a GBM stochastic process. The average output of the GBM process can be approximated with the following expression ( Lu *et al.*, 2017):

$$I_{OM}(t)=\left(\frac{1}{\delta }\right)I_{C}H_{0}e^{\left(-\frac{\sigma_{H}^{2}t}{2}+\sigma_{H}\varepsilon_{H}\sqrt{t}\right)}\;(16)$$

The accuracy of the approximation ( 16) has been validated comparing it against the outputs of an actual Geometric Brownian Motion stochastic process ( QuantPy, 2024), using parameters included in the case study in Table 1. Thirty Monte Carlo runs were performed, obtaining a mean Root Relative Square Error (RRSE) of 0.03718 with a standard deviation of 0.01536, which prove the accuracy of the approximation.

### 3.3 Business model CANVAS

In the previous Section 3.2, the Net Present Value (NPV) calculation for building owners, tenants, and the energy service company for the smart energy service was presented, to allow assessing its financial viability in a rented commercial property. This section builds on that analysis by developing a business model using the Business Model Canvas. For energy service companies seeking to implement the smart energy service, a clear understanding of how to financially evaluate Energy Performance Contracting (EPC) is essential. At the same time, the financial calculations are valuable for landlords and tenants, as they highlight the key variables to consider in order to make well-informed and optimal decisions. This connection between financial assessment and business model design has received limited attention in the literature, and its integration represents a key contribution of this study.

The Business Model described in this paper is developed using the CANVAS template ( Table 5), which is a comprehensive visual chart proposed in ( Osterwalder & Pigneur, 2010), that covers all the aspects of the Business Model. Such template is a strategic management and entrepreneurial tool which allows to describe, design, validate a Business Model, highlighting its value proposition, infrastructure, customers and financial aspects. The Business Model can be adopted by organisations of any size, ranging from large enterprises to small-sized start-ups. It shows how an organisation can successfully run a business related to delivering energy savings to commercial companies. The CANVAS template consists of nine building blocks, namely Key Partners, Key Activities, Key Resources, Cost Structure, Value Proposition, Customer Relationships, Channels, Revenue Streams, Customer Segments. The four building blocks on the left-hand side of the CANVAS provide information about the efficiency-side of the business model (Key Partners, Key Activities, Key Resources, Cost Structure) and offer insights on how to scale up the business efficiently. The five building blocks on the right-hand side represent the value (Value Proposition, Customer Relationships, Channels, Revenue Streams, Customer Segments), which is delivered to the right customers who are willing to pay for the service. The **key partners** represent mainly strategic alliances with non-competitors or even joint ventures to develop the proposed business. The **key activities** describe the most important actions that a company must do to make the business model working. The key activities are required to create and offer a value proposition (e.g., a reduced energy consumption, while maintaining the same level of satisfaction for the energy consumer), to reach the market of smart energy services, creating and maintaining effective customer relationships, and earning revenues. The **key resources** are the most important assets required to make the business model work. The key resources allow the ESCO to create and offer a value proposition, reach out to the market, maintain relationships with customer segments and earn revenues. The **cost structure** section describes all the costs incurred to operate a business model, to create and deliver value, maintain customer relationships, and generating revenues. The **value proposition** defines the bundle of products and services that create value for one or more customer segments. The **customer relationship** section describes the types of relationships that the energy efficiency service provider/ESCO establishes with specific customer segments, including the customer acquisition, customer retention, boosting sales and upselling. The **channels** section describes how the company that implement the business model communicates with and reaches its customer segments to deliver its value proposition. The **revenue streams** represent the cash that the ESCO generates for each customer segment. The **customer segments** define the different types of commercial buildings and companies that an energy efficiency service provider/ESCO aims to reach and serve. Distinct segments are characterized by common needs, common behaviour or other attributes. The profitability of the different market segments may be substantially different as it depends on the willingness of the customers to pay for the proposed product or service.

**Table 5.** Business model for the implementation of the smart energy service in commercial rented buildings.

**Key Partnerships** Materials and components suppliers and installers (smart devices, renewable energy sources, energy storage components and infrastructure, e-mobility infrastructure, smart monitoring & management platform). Building automation and technology installers. Building services engineering companies. Project financiers. Energy Performance Contracting Facilitators. Building services engineers and designers. Energy efficiency consultants.	**Key Activities** Recommend energy efficiency measures. Elaborate an implementation plan for energy efficiency measures. Define the energy efficiency service price (service fee paid by clients) and plan of payments. Implement agreed energy efficiency measures. Collect energy consumption data (system monitoring) and optimise the installed measures. Implement the energy performance contract. Measure and verify energy savings and determine recurring payments of clients. Perform system maintenance regularly. Train clients on how to use of their equipment and appliances to reduce energy consumption.	**Value Proposition** Increased value and greener image of the property after installation of energy efficiency measures and BMS upgrades. Renovated property and non-energy benefits such as increased indoor comfort (e.g. thermal comfort and air quality). Monthly service fee paid by the renters to the ESCO for the energy efficiency service. Monthly payment by ESCO to the building owner to share the value of the energy savings achieved by renters. The service fee paid by the building owner to the ESCO (optional, applicable only if the building owner occupies part of the building). The responsibility taken by the ESCO for the installation, commissioning, monitoring and regular maintenance of equipment, devices and systems and the provision of guaranteed/shared energy savings to their clients.	**Customer Relationship** Agreement/SmartSPIN Contract between ESCO and clients about energy efficiency measures to be installed and the subject responsible for project financing (ESCO or building owner). Negotiation about the recurring service fee payment performed by each client to the ESCO. Invoice for the energy efficiency service sent by ESCO to clients. Receipt for the recurring payment received by the building owner from the ESCO.	**Customer segments** Building owners and renters of different typologies of rented commercial properties, such as: - Industrial buildings (e.g. manufacturing facilities, warehouses, etc.) - Retail (e.g. clothing stores, commercial banks, restaurants, etc.) - Offices - Healthcare facilities - Hotels and hospitality buildings - Special or mixed purpose buildings (e.g., airports, shopping centres, recreational centres etc.)
	**Key Resources** Capital from project financiers and government grants for the installation of energy efficiency measures. Contractual templates to implement the smart energy service in commercial rented properties. Energy efficient equipment and measures. Technologies for smart controls. Building Management Systems. Meters, sensors, data loggers. Gamification app, building performance diagnostic app, measurement and verification app.		**Channels** Website and blog. Printed posters displayed at strategic locations. Printed or digital flyers or brochures. Social media channels. Printed or emailed newsletters. Press releases. TV or radio advertisements. Outreach at trade shows and sectoral events. Outreach via building owners and managers associations. Business referral.	
**Cost Structure** Purchase, installation and financing cost of energy efficient equipment. Operation and Maintenance costs of installed equipment. Fees of consultants, contractors and ESCO personal. Costs associated with the measurement and verification of energy savings. . Recurrent monetary reward to the building owner. Marketing and advertisement costs.			**Revenue Streams** Main revenue stream associated with the share of energy cost savings obtained by the clients, i.e., renters and building owner (the latter only if they occupy part of the building). Revenue stream for the operation and maintenance service. Additional revenue stream for equipment rent, in case the ESCO owns the equipment and rents it to the client for a fixed monthly fee. All the revenue streams are collected through the service fee.	

### 3.4 Value proposition CANVAS

The Business Model CANVAS (BMC) described in the previous Section 3.3 provides the necessary information to the businesses (energy efficiency providers, ESCOs or similar) that want to implement the smart energy service in rented commercial buildings. The BMC identifies the value proposition within the broader business model and connects it to the channels through which the ESCO delivers the service, the customer relationships maintained, the resources and partners required to implement and monitor energy efficiency measures, and the revenue streams generated from service fees and energy savings. To refine the value proposition of the proposed service previously identified in the BMC, and ensure that the service offer is aligned with what the customer needs, the Value Proposition CANVAS template is used ( Osterwalder *et al.*, 2014). This task consists in determining how the benefits, features and experience with the considered smart energy service create value for the customers addressing their wants, needs and fears. The CANVAS template also includes specific information on the product/service, the profile of its ideal customer and the existing substitutes. The value proposition defines the bundle of products and services that create value for one or more customer segments. The value proposition is an aggregation of benefits that ESCOs offer to customers. The Value Proposition CANVAS is divided into two sections: Product/Service and Customer. The former section is divided into benefits, features, experience, product and ideal customer sections. The latter section is divided into wants, needs, fears and substitutes. The benefits section includes the benefits delivered by the proposed service to the customer, that make customer’s operations easier by increasing satisfaction or decreasing discomfort. The **benefits** of the proposed smart energy service are the core of its value proposition. The features are the part of the value proposition consisting in the functional attributes that factually describe how the proposed smart energy service works. The features are the “reasons to believe” for the customer. The **features** are the part of the value proposition consisting in the functional attributes that factually describe how the proposed service works. The features are the “reasons to believe” for the customer. The service **experience** is the way that receiving the service makes the customer feel. The customer’s experience results from the service’s features and benefits. The service experience differs from features and benefits because it relates more to the emotional reasons why customers want to purchase the service and the value it delivers to them for their own businesses. The service experience can be used to identify how the proposed service should be positioned in the market and how the brand should communicate the value proposition to the potential customers. The ‘product’ section states what product or service is described in the Value Proposition CANVAS. The ‘ideal customer' section states who the ideal customer for the proposed product or service is. **Wants** are the customer’s thoughts about how they would like to improve the activities in their business that are relevant with the considered product or service. They are the emotional drivers of decision-making that can motivate customer’s actions. The customer’s **needs** are the actions that the customer needs to get done. Customers might not be aware of all their needs. Needs that are not known yet by the customer yet are called “latent needs”. The customer’s needs can be determined following a rational reasoning. **Fears** are the hidden source of wants and needs for the customer and can be seen as a driver for purchasing the proposed service, if it addresses the wants and needs of the customer. Customers’ fears may also be related to the perceived efforts of switching service or service provider. To win such fear of change, the proposed service must deliver very significant improvements to overcome the reluctance of the customers to change their current processes. A **substitute** is a product or service that serves the same purpose as the product/service described in the Value Proposition CANVAS template.

## 4. Results

### 4.1 Case study

Understanding the factors that determine the financial feasibility and profitability of an energy performance contract, together with the decision-making processes of landlords and tenants, is fundamental for an ESCO seeking to implement a smart energy service in a rented commercial property. Analysing these factors allows the ESCO to assess which EPC contracts are economically viable, while ensuring that contract duration, pricing structures, and performance guarantees are consistent with the financial objectives and constraints of its clients. Such an understanding enables the ESCO to formulate a value proposition that is both financially attractive and operationally acceptable to landlords and tenants. In the absence of this quantitative insight, the qualitative components of the Business Model Canvas alone may be insufficient to mitigate the risks associated with implementing the smart energy service.

In the following of this section, we consider the case study whose parameters are included in the Table 1 below.

**Table 1.** Parameters used for the case study.

Parameter	Description	Equations	Value	Units
*I _C_*	Total investment in energy efficiency measures	( 4– 5)	3,840,000	€
*G*	Annual energy cost savings guarantee to the clients	( 1– 6)	250,000	€/year
*β*	Fraction of the excess savings beyond the savings guarantee taken by the building owner	( 1– 6)	0.2	-
n	Contract duration	( 1– 6)	14, 15, 16, 17, 18, 19, 20, 21	Years
N	Energy efficiency measures lifetime	( 1– 6)	25	Years
*θ*	Fraction of energy savings taken by the building owner	( 1– 6)	ranging from 0.5 to 1	-
*P*_*E*0_	Initial energy price	( 15)	0.24	€/KWh
α *_E_*	Annual price drift	( 15)	0.0523	-
σ *_E_*	Annual price volatility effect	( 15)	0.856	-
*ε _P_*	Random error for energy price	( 15)	0.01	-
*K*_0_	Potential energy savings per unit of initial investment of retrofit	( 12)	0.43	kWh/€
*σ _K_*	Volatility coefficient for energy savings potential	( 12)	0.01	-
*ε _K_*	Random error for energy savings potential	( 12)	0.01	-
δ	Coefficient describing the change of the O&M cost over project lifetime	( 16)	1.025	-
*H*_0_	Initial value of the O&M cost per unit of initial investment of retrofit	( 16)	0.0036	€/€
*σ _H_*	Volatility of annual O&M cost	( 16)	0.25	-
*ε _H_*	Random error for O&M cost	( 16)	0.01	-
*γ*	Share of the investment performed by the building owner	( 4, 5, 7, 8)	0	-
*r*_0_	Building owner’s rate of return	( 5)	3.1	%
*r _R_*	Renters’ rate of return	( 6)	3.1	%
*r _E_*	ESCO’s rate of return	( 4)	6	%

Figure 6 shows the cash flow diagrams illustrating the financial exchanges over the project time frame for contract duration n = 15, *θ* = 0.8, *γ* = 0.35, *ϕ* = 0.1 *ρ* = –20, and other parameters included in Table 1. These cash-flows result in a NPV for ESCO, building owner and renters of € 1,313,971, € 1,476,850, and € 500,397 respectively. It can be noticed that the cash flow is negative for the ESCO and building owner at t = 0 as they perform the investment to install energy efficiency measures. The ESCO achieves a positive cash flow during the EPC contract (1 ≤ *t* ≤ *n*) and zero after the end of the contract ( *n* + 1 ≤ *t* ≤ *N*). It can also be noticed that after the end of the contract, cash flows increase for the building owner and renters, as they no longer share energy savings with the ESCO. It can also be noted that at t = N the energy efficiency measures reach their end of life and no longer produce energy savings. The slightly negative cash flow for the building owner at t = N represents the O&M costs.

**Figure 6.**
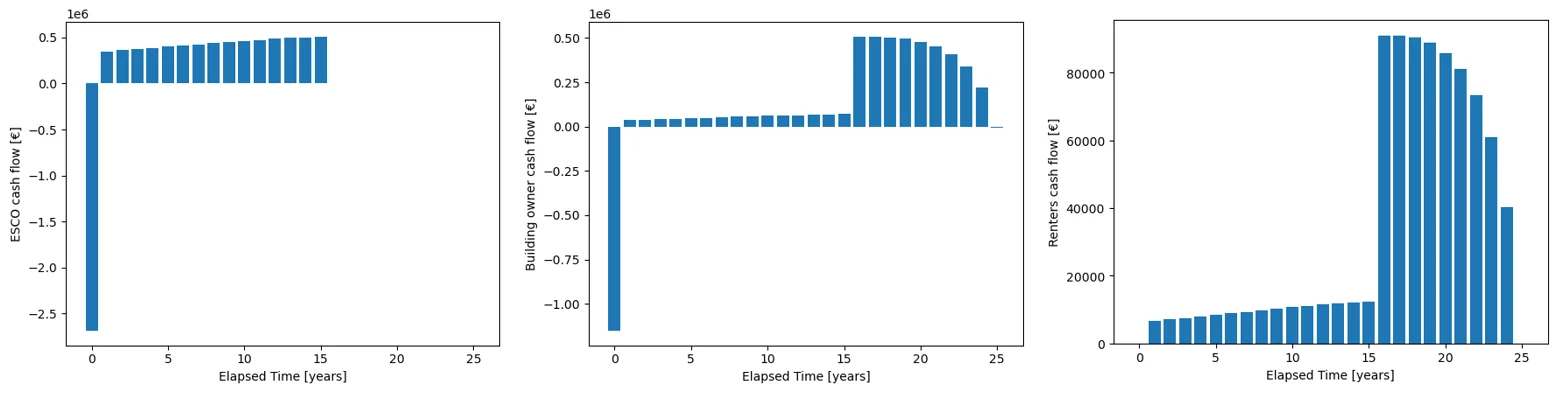
Cash flow diagrams for ESCO (left hand side plot), building owner (centre plot) and renters (right hand side plot).

### 4.2 Factors that determine the decision-making for the efficiency provider, building owner and renters

***4.2.1 Factors that determine the decision-making for the energy efficiency provider***

The factors that influence the decision of an energy efficiency provider (ESCO) to enter an energy performance contract are: 1. Whether the building owner performs in part or totally the investment required to install energy efficiency measures; 2. The agreements between building owner and renters with respect to sharing the energy savings and the agreement between renters and energy efficiency provider in relation to the service fee to be paid for the energy efficiency service. 3. The behaviours of the renters which may determine a rebound effect, i.e. an increment in their energy consumption following the installation of energy efficiency measures ( Berkhout *et al.*, 2000; Sorrell *et al.*, 2009);

We will thoroughly analyse the case where the ESCO performs the investment, i.e., *γ* = 0 in ( 4) and ( 5), as that is the case where the decision making may become more complicated for the ESCO and at the same time it is the most attractive for the potential clients.

Results in Figure 7 show that especially for shorter contract durations, the ESCO should pay attention to the maximum rebound effect and the risk tolerance of renters, as when they are high, high values of the percentage of the energy savings kept by the building owner might determine economic losses for the ESCO. If these parameters cannot be estimated with satisfactory accuracy, the ESCO should propose a contract with longer duration to mitigate the risk of economic losses due to lower energy savings caused by renters’ rebound effect. The sensitivity of the maximum percentage of energy savings kept by the building owner which is acceptable for the ESCO *θ _max_* with respect to the variations of the risk tolerance *ρ* is even higher than the sensitivity with respect to the variations of the maximum rebound effect *ϕ* because *ρ* appears in the denominator of the exponent of the exponential function in ( 11), whereas *φ* is a multiplying factor of two terms of the sum in ( 11).

**Figure 7.**
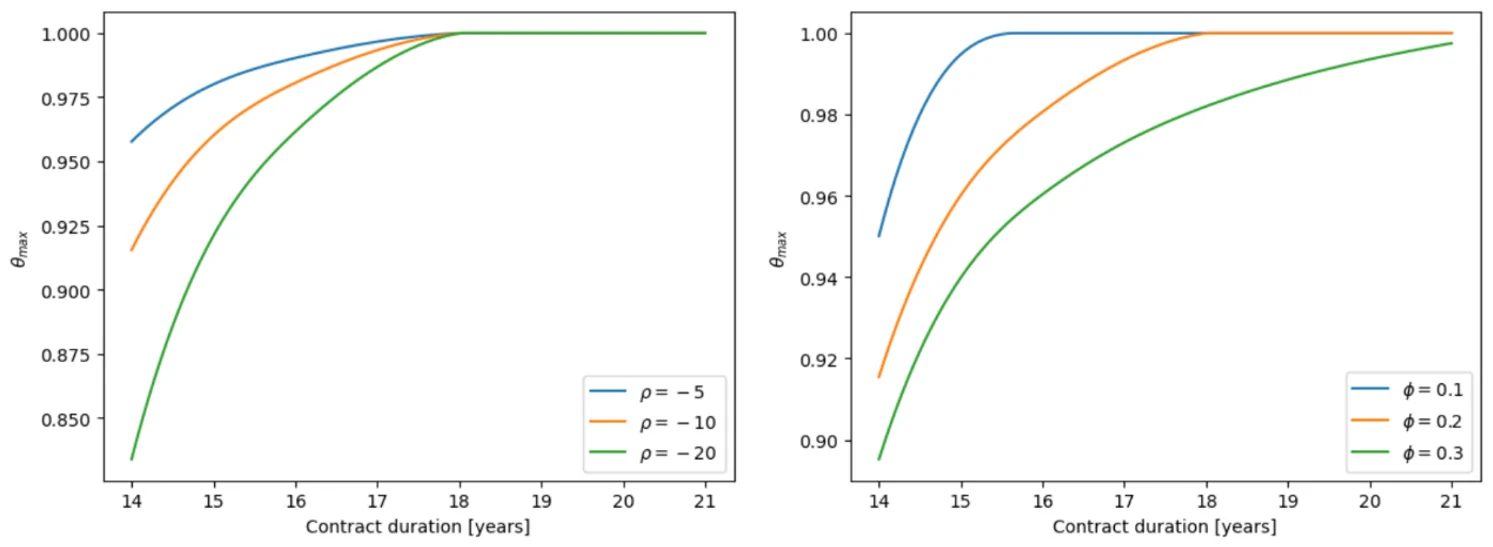
Maximum percentage of the energy savings kept by the building owner acceptable for the ESCO for different values of contract duration, *ρ* and *φ* (respectively left-hand side figure and right-hand side figure).

The sensitivity of NPV for the ESCO for various stakeholder groups characterised by different rebound effect parameters ( *φ* and *ρ*) at varying contract lengths is reported in Table 2.

**Table 2.** Sensitivity of NPV for the ESCO for various stakeholder groups at varying contract lengths.

Stakeholders group	Rebound factor	Net Present Value for ESCO [€]			
		Contract duration			
		14 years	15 years	16 years	17 years
Group 1 ( *φ* = 0.1, *ρ* = –10)	0.995	119,140	335,214	540,734	735,057
Group 2 ( *φ* = 0.2, *ρ* = –10)	0.990	100,573	315,627	520,177	713,583
Group 3 ( *φ* = 0.1, *ρ* = –20)	0.978	56,389	269,016	471,258	662,482
Group 4 ( *φ* = 0.2, *ρ* = –20)	0.956	−24,930	183,231	381,225	568,434

It is worthwhile to note that, if a tenant fails to pay the service fee to the ESCO due to financial difficulties, or if a space remains unleased, the ESCO may experience reduced cash flows. Similarly, when a lease expires and the tenant moves out, the ESCO may lose the associated revenue stream unless a new tenant immediately occupies the space. To account for these scenarios, the expected cash flow for the ESCO can be adjusted by the probability of payment, which results in a lower net present value compared to a situation with guaranteed and uninterrupted payments. Higher uncertainty or risk may also require the ESCO to increase service fees, potentially affecting project feasibility. Contracts can include provisions to mitigate these risks and ensure that the EPC remains financially sustainable and attractive for all parties involved. For example, guarantees from the building owner can shift credit risk by ensuring that the owner covers the ESCO’s fees if a tenant fails to pay, allowing the ESCO to receive expected payments even in case of default. Insurance policies can also protect the ESCO by compensating for missed payments due to tenant insolvency, reducing the impact of defaults on project NPV. Reserve funds, created by setting aside a portion of collected fees, provide a buffer to cover periods of non-payment or vacancy, enabling the ESCO to continue operations during temporary shortfalls. Minimum guaranteed payments establish a baseline revenue for the ESCO regardless of tenant occupancy or energy savings performance, ensuring that essential costs are covered. Finally, fees tied to occupancy adjust payments based on the number of tenants in the building, increasing when occupancy is high and decreasing when it is low, which aligns the ESCO’s remuneration with actual risk and performance.

***4.2.2 Factors that determine the decision-making for the building owner***

We consider in this section the case where the building owner does not occupy any significant spaces in the building. In fact, if the building owner occupies the premises, wants to modernize the building and accrues some energy savings once that the energy efficiency measures are installed, the split incentive is a less significant issue and the payment of a service fee from the tenants may not necessarily be determinant with respect to the decision of investing in energy efficiency measures. Building owners tend to bargain for a shorter contract duration (as opposed to ESCOs that bargain for a longer duration) as they are aware that the energy efficiency measures installed can still produce additional savings beyond the length of the contract, which will not have to be shared with the ESCO after the end of the EPC ( Li *et al.*, 2014). Indeed, the objective of building owners is to maximise their NPV while contracting with an ESCO. It must be pointed out that that under an EPC, the ESCO takes the full responsibility for the maintenance of the installed equipment and provides training to its client to operate the systems at their best performances. During the contract term, faulty equipment is replaced at the ESCO’s own expense. Such a comprehensive service is appreciated by the building owners. Guo and Zhang proposed an approach to optimise the guaranteed savings value G that appears in Equations ( 1– 6), which consists in finding the value *G _opt_* value that minimises absolute value of the difference between building owner’s investment and ESCO’s investment. The contract duration can be adjusted comparing the NPVs of the building owner and ESCO as it was done in ( Deng *et al.*, 2014); in addition, the perspective of the renters must be considered when the considered property is rented. This perspective will be analysed in the next Section 2.2.3. Another factor that needs to be taken into consideration when determining the EPC agreements is the share of energy savings with the renters. Under the assumption that the rebound effect in energy consumption of the renters is not negligible (which was put forward in the previous Section 2.2.1), the building owners need to take it into consideration to determine the optimal share of savings with the renters, i.e., (1 – *θ*) in Equation (2) and Equation (6). The optimal share of *θ _opt_* depends on the maximum rebound effect *φ* and the risk tolerance *ρ* (see also De Tommasi & Agrawal, 2023). It can be observed that when the rebound effect increases from *φ* = 0 to *φ* > 0 the NPV curves for the building owner (which are straight segments for *φ* = 0) start curving ( Figure 8). Increasing *φ* will lead to a decrease of *θ _opt_* and for *φ* > *φ′* > 0 the maximum NPV will be obtained with a *θ _opt_* < 1 ( Figure 9).

**Figure 8.**
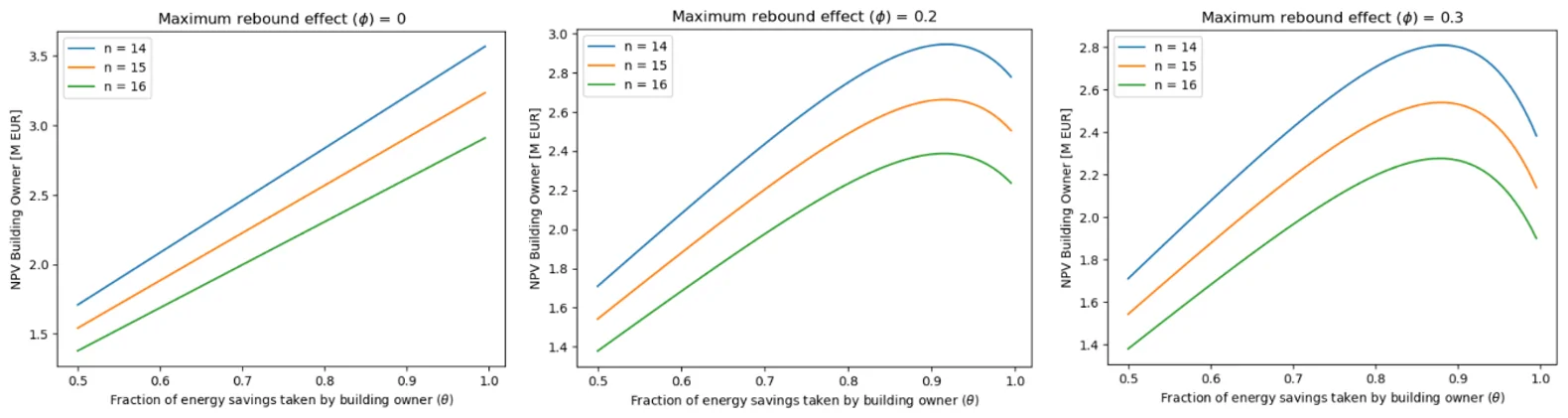
Net Present Value for building owner as a function of *θ* for different contract durations ( *n* = 14, 15, and 16 years). Left hand side panel: case *φ* = 0. Centre panel: *φ* = 0.2. Right hand side panel: *φ* = 0.3.

**Figure 9.**
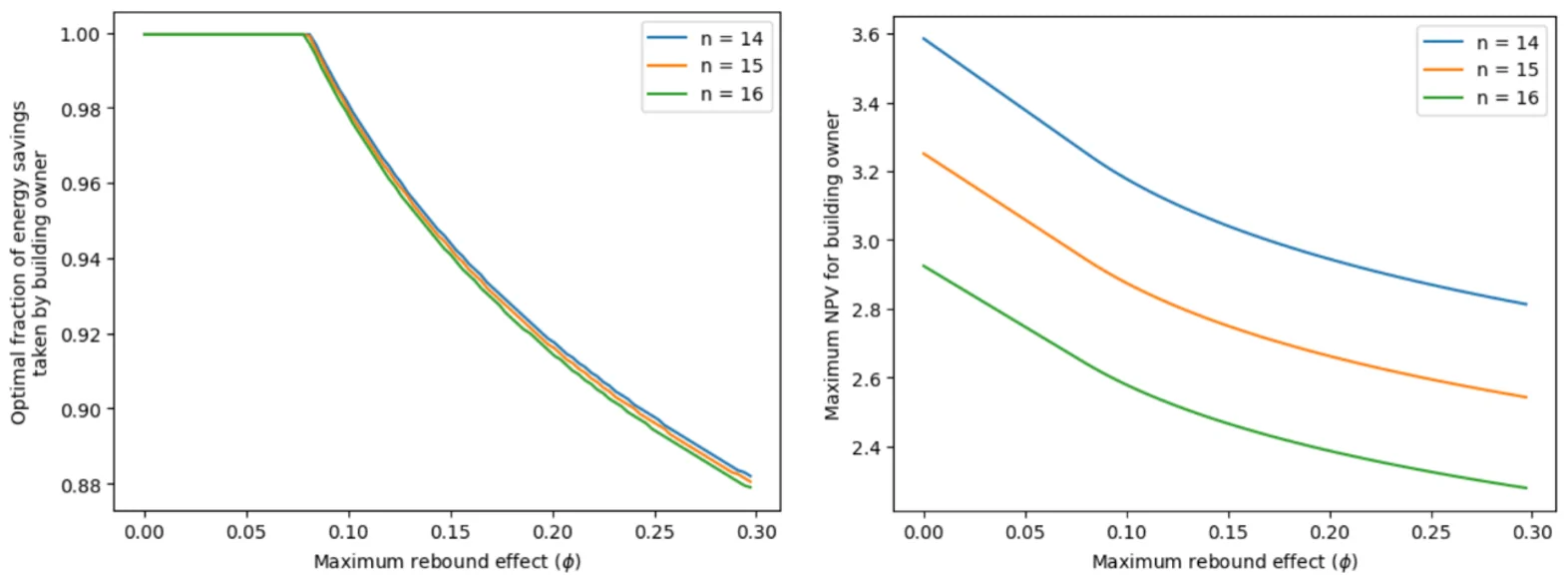
Left hand side panel: Optimal fraction of energy savings taken by building owner as a function of the maximum rebound effect *φ* for different contract durations (n = 14, 15, and 16 years). Right hand side panel: Maximum NPV for building owner as a function of the maximum rebound effect *φ* for different contract durations (n = 14, 15, and 16 years).

The sensitivity of NPV for the building owner for various stakeholder groups characterised by different rebound effect parameters ( *φ* and *ρ*) at varying contract lengths is reported in Table 3.

**Table 3.** Sensitivity of NPV for the building owner for various stakeholder groups at varying contract lengths.

Stakeholders group	Rebound factor	Net Present Value for building owner [€]			
		Contract duration			
		14 years	15 years	16 years	17 years
Group 1 ( *φ* = 0.1, *ρ* = –10)	0.995	3,006,139	2,723,119	2,446,522	2,177,775
Group 2 ( *φ* = 0.2, *ρ* = –10)	0.990	2,988,485	2,706,778	2,431,466	2,163,969
Group 3 ( *φ* = 0.1, *ρ* = –20)	0.978	2,946,473	2,667,893	2,395,639	2,131,113
Group 4 ( *φ* = 0.2, *ρ* = –20)	0.956	2,869,152	2,596,328	2,329,702	2,070,644

***4.2.3 Factors that determine the decision-making for the renters***

According to the business model illustrated in Figure 1 and Figure 2, the smart energy service delivers guaranteed energy savings to the renters; however, since the renters do not bear the investment costs of the energy retrofitting project, they are required to pay a service fee to the ESCO. The ESCO uses such payments to share the value of the energy savings with the building owner. Although there might be non-energy benefits delivered to the renters such as a renovated property or improved thermal comfort, a requirement in many rented commercial properties is that the service fee paid to the ESCO is not greater than the energy savings achieved. For simplicity, in this analysis we aggregate all the renters’ energy cost savings and service fee payments. The monthly energy costs before the retrofit (baseline) are calculated as follows:

$$E_{C,bas}=E_{el,bas}\cdot P_{el}+E_{ng,bas}\cdot P_{ng}\;(17)$$

where *E _el,bas_*, *E _ng,bas_*, *P _el_*, and *P _ng_* are respectively the baseline energy consumption of electricity, the baseline energy consumption of natural gas, the price of electricity per kWh, and the price of natural gas per kWh. The monthly energy costs after the retrofit are:

$$E_{C,ret}=E_{el,ret}\cdot P_{el}+E_{ng,ret}\cdot P_{ng}\;(18)$$

The energy cost savings are:

$$\text{Δ}E_{C}=E_{C,bas}-E_{C,ret}\;(19)$$

The renters approve the EPC if:

$$\text{Δ}E_{C}-S\geq 0\;(20)$$

where *S* is the total value of the monthly service fees paid by the renters to the ESCO. The difference *ΔE _C_ – S* represents the monthly energy cost savings shared with the renters:

$$12\cdot (\text{Δ}E_{C}-S)=R_{R}\;(21)$$

where *R _R_* is the annual revenue stream for the renters ( 2). It is worth noting that if the service fee is set equal to the monthly energy bills savings *ΔE _C_ – S*, then there are no actual energy savings shared with the renters as they would pay the same amount saved on the monthly bills to the ESCO for the smart energy service (which would share that value with the building owner), that corresponds to setting *θ* = 1 in ( 2),( 3),( 5),( 6).

The sensitivity of NPV for the renters for various stakeholder groups characterised by different rebound effect parameters ( *φ* and *ρ*) at varying contract lengths is reported in Table 4.

**Table 4.** Sensitivity of NPV for the renters for various stakeholder groups at varying contract lengths.

Stakeholders group	Rebound factor	Net Present Value for renters [€]			
		Contract duration			
		14 years	15 years	16 years	17 years
Group 1 ( *φ* = 0.1, *ρ* = –10)	0.995	560,087	510,143	461,331	413,906
Group 2 ( *φ* = 0.2, *ρ* = –10)	0.990	556,972	507,259	458,675	411,469
Group 3 ( *φ* = 0.1, *ρ* = –20)	0.978	549,558	500,397	452,352	405,671
Group 4 ( *φ* = 0.2, *ρ* = –20)	0.956	535,913	487,768	440,716	395,000

### 4.3 Factors that determine the contract duration

The long contract duration of Energy Performance Contracts is a barrier in most Member States EPCs to their penetration in the commercial rented sector because an EPC duration of 8–15 years (which is quite common) is in many cases longer than the tenancy contracts. The contract duration is determined by the initial investment performed by the ESCO, the energy savings generated by the energy efficiency measures *R(t)*, by the guaranteed savings *G* provided by the ESCO to the customer, by the fraction of savings *β* shared with the customer above the threshold *G*, the share of the savings *θ* between building owner and renters. A higher investment for the ESCO will determine generally a longer contract duration to recover the investment and make a profit. On the other hand, higher energy savings *R(t)* will facilitate a contract of shorter duration, whereas higher guaranteed/shared savings ( *G* and *β*) will reduce the value of the savings taken by the ESCO and possibly determine a longer contract duration. The study reported in ( Collins *et al.*, 2018) found that in UK building owners and their investors are keen to invest in energy efficiency technologies in their new or retrofitted buildings despite the upfront costs, because of the long-term financial benefits as well as those associated with corporate social responsibility. The same study concludes that the main barriers for building owners and tenants with respect to the implementation of energy efficiency technologies lie in the bureaucratic and technical domains. Many published papers assume that either the ESCO or the building owner would take full responsibility of the initial investment in energy efficiency measures *I _C_*. In ( Li *et al.*, 2014), the investment share between ESCO and its client was considered as one of the EPC contract terms. However, the model proposed in ( Li *et al.*, 2014) did not consider a rented building. In this paper, we introduced the sharing factor *γ* in *NPV _E_* and *NPV _0_* (such that the ESCO invests *α* = 1 – *γ* whereas the building owner invests *γ*) considering the EPC model which applies to commercial rented buildings. *NPV _E_* can be increased reducing *α*, which allows to fulfil the condition *NPV _E_* ≥ *NPV _E,min_* with a shorter contract duration ( Figure 10). In other words, to mitigate the risk of tenants leaving before the end of the EPC, a building owner will have to take the responsibility of part of the initial capital investment and negotiate the appropriate contract duration with the ESCO.

**Figure 10.**
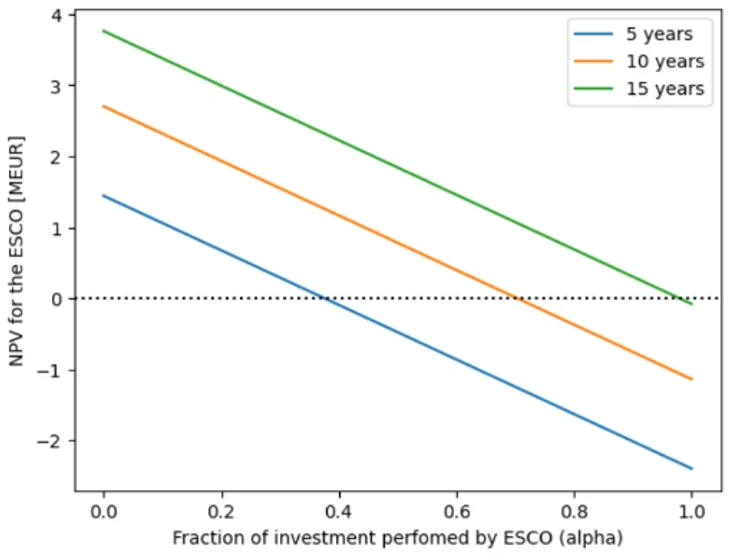
NPV for the ESCO as a function of the fraction of the investment performed by the ESCO and contract duration (5 year, 10 years and 15 years).

### 4.4 Practical implementation of a business model for a smart energy service for the commercial rented sector

***4.4.1 Business model***

This section discusses the development of the business model for the smart energy service for the commercial rented sector proposed in this paper, applying the business model CANVAS previously introduced in Section 2.3. Results of this analysis are presented in Table 5. The business model development discussed in this paper builds on the previous work in ( De Tommasi *et al.*, 2024; Ring *et al.*, October 2022; Skordoulias *et al.*, 2024).

### Key activities

Key activities include recommending energy efficiency measures, developing an implementation plan, determining the service price (i.e., the fee paid by clients to the ESCO), and establishing a payment schedule. They also cover the implementation of agreed measures, monitoring system performance by collecting energy consumption data, and optimizing the operation of installed solutions. Additional activities involve executing the energy performance contract, measuring and verifying energy savings ( Papadelis, 2023), and defining recurring client payments. Furthermore, the ESCO is responsible for ongoing system maintenance and providing training sessions to clients on optimal equipment and appliance use to reduce energy consumption.

### Customer relationship

In the proposed business model, the customer relationship is defined through contractual agreements between the ESCO and its clients, outlining the energy efficiency measures to be implemented and the party responsible for project financing (either the ESCO or the building owner). The contract may also establish a tripartite agreement between the ESCO, landlord, and tenants ( Ring *et al.*, 2023). It specifies the service duration and the service fee payable to the ESCO. Typically, tenants are responsible for paying this fee, while the ESCO shares part of the value generated with the building owner. In certain cases, the building owner may also pay a service fee, for example, if they occupy the building and directly benefit from the energy savings. The customer relationship further encompasses the negotiation of recurring service fees, the invoicing of clients by the ESCO, and the subsequent transfer of a share of collected payments to the building owner.

### Customer segment

In the commercial sector, customer segments include building owners and tenants of various rented property types, such as shopping centres, industrial facilities (e.g., manufacturing plants, warehouses), retail spaces (e.g., clothing stores, banks, restaurants), offices, healthcare facilities, hotels, hospitality buildings, and special or mixed-use properties (e.g., airports, recreational centres). Effective segmentation of this market can be based on factors such as the number and size of buildings, the number of tenants, and the share of total floor area occupied by the building owner compared to renters. Additional target customers may include facility managers, companies, and energy users operating within rented premises.

An ESCO adopting the business model described in Table 5 must strategically determine which customer segments to prioritize. Smaller ESCOs with limited resources and staff may focus on smaller clients, such as medium-sized office buildings. In such cases, the customer relationship is primarily established with the building owner, while the involvement of tenants is usually limited to providing consent for works affecting their premises and agreeing to pay the service fee.

In larger or more complex segments, the ‘split incentive’ issue becomes more prominent, requiring a balanced customer relationship involving the ESCO, building owner, and tenants. While the service offering remains fundamentally similar across segments, delivery of an energy performance contract in rented commercial buildings, the specific contractual clauses and agreements vary. These differences shape how energy savings are distributed among the parties, influencing the respective benefits for the ESCO, the building owner, and the tenants.

### Key partners

Key partners include suppliers and installers of materials, smart devices, renewable energy systems, energy storage components and infrastructure, and e-mobility infrastructure. They also comprise providers of smart monitoring and management platforms, building automation technologies, and building services engineering solutions. In addition, project financiers, Energy Performance Contracting (EPC) facilitators, building services engineers and designers, as well as energy efficiency consultants, play a central role.

### Key resources

The core idea of the proposed business model is to generate value for both building owners and tenants. Delivering the energy efficiency service requires several key resources, including project financing from investors and government grants to support the installation of efficiency measures, as well as contractual templates tailored to smart energy services in rented commercial properties. Other essential resources comprise energy-efficient equipment, smart control technologies, Building Management Systems, meters, sensors, and data loggers. Digital tools also play a critical role, such as a visualization dashboard for monitoring energy consumption ( Diez *et al.*, 2024), a gamification app ( Iria *et al.*, 2020), a building performance diagnostic app ( Eguiartea *et al.*, 2023), and a measurement and verification app ( Gallagher *et al.*, 2019). The visualization dashboard and gamification app developed by Smarkia within the H2020 SmartSPIN project are shown in Figure 11 and Figure 12, respectively. These resources may be owned or leased by the ESCO or building owner, or provided by key partners.

**Figure 11.**
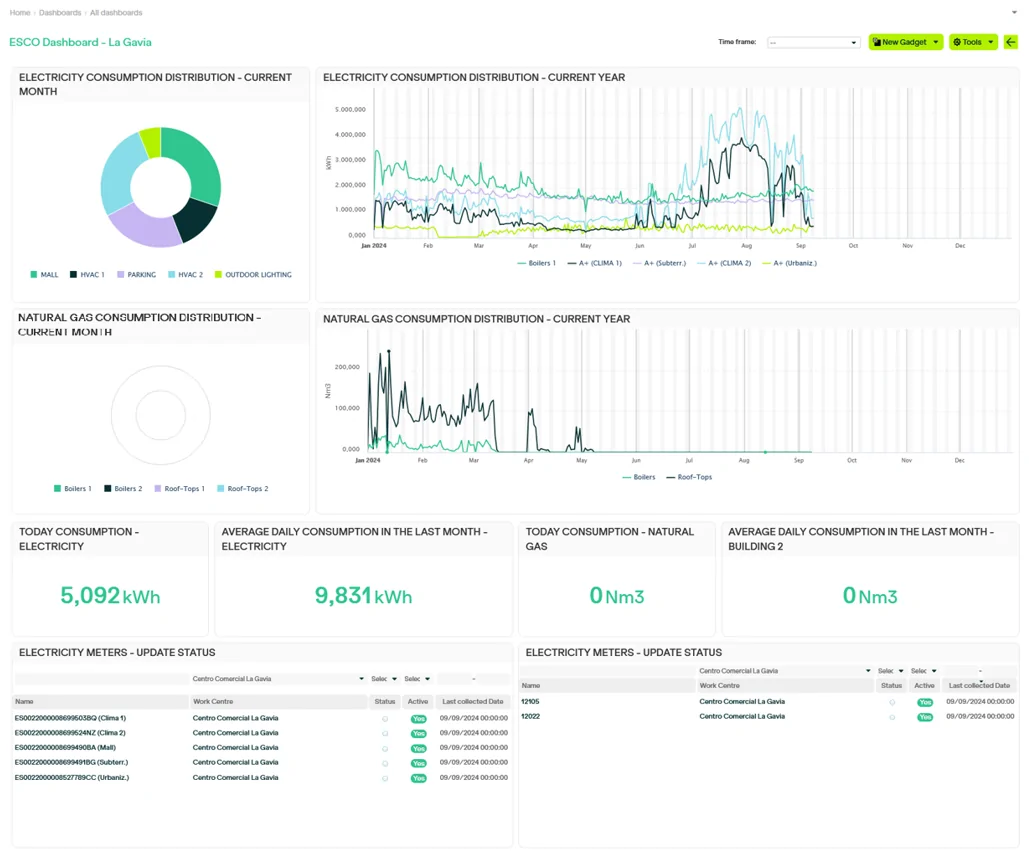
Visualisation dashboard developed by Smarkia in the H2020 SmartSPIN project.

**Figure 12.**
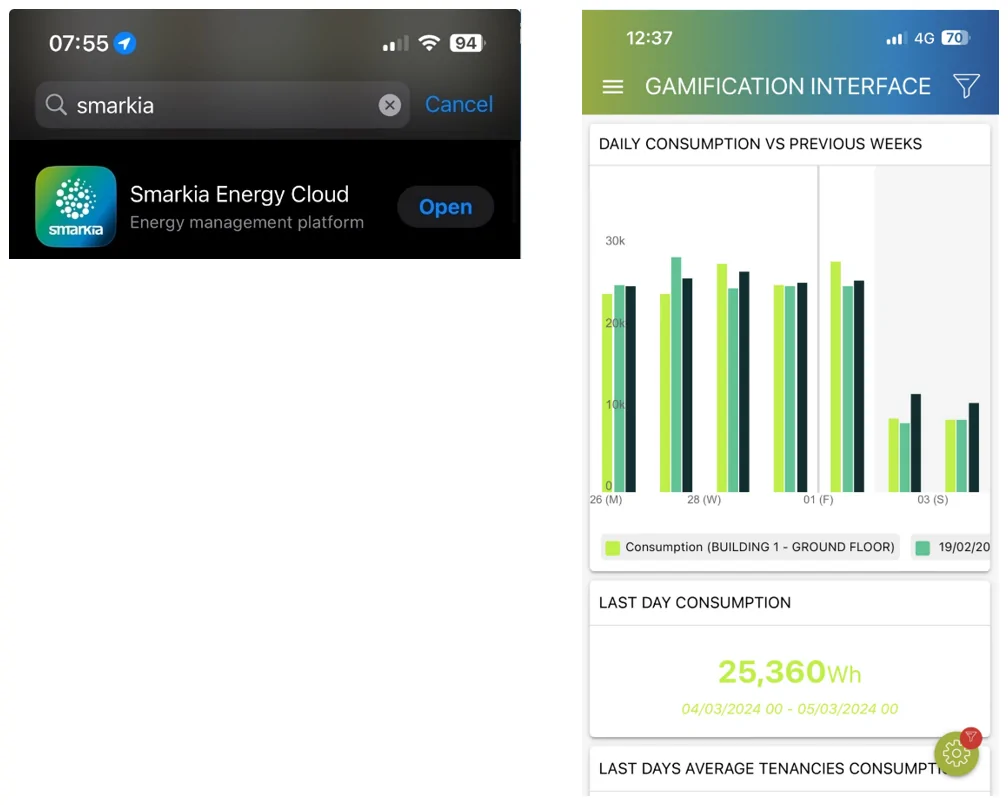
Gamification app developed by Smarkia in the H2020 SmartSPIN project.

### Value proposition

The proposed smart energy service enhances the value and green image of commercial properties by integrating energy efficiency measures and Building Management System (BMS) upgrades. Beyond energy savings, it delivers non-energy benefits such as property renovation, improved thermal comfort, and better indoor air quality.

For ESCOs, value is generated through monthly service fees paid primarily by tenants (and, in some cases, by building owners). The value proposition combines multiple benefits: reduced energy bills, enhanced comfort, improved environmental performance, and in some cases, reductions in water waste. The service’s value to customers is reinforced by the ESCO’s responsibility for installation, commissioning, monitoring, maintenance, and the delivery of guaranteed or shared energy savings.

Typical value-creating measures include heat pumps, photovoltaic panels, LED lighting, building management systems, and smart building controls. Sub-services such as equipment replacement, ongoing maintenance, and measurement and verification of savings complement the offering.

The value proposition outlined in Table 5 is innovative and disruptive, as it adapts energy performance contracting to the specific context of commercial rented buildings, enabling both owners and tenants to benefit.

### Channels

Communication, distribution, and sales channels serve as the ESCO’s primary interface with customers. Their functions include raising awareness of offered products and services, helping customers evaluate the ESCO’s value proposition, enabling the purchase or lease of specific products and services, delivering the value proposition, and providing post-purchase support.

Key channels include the company website and blog, printed posters in strategic locations, flyers or brochures (printed or digital), social media, newsletters (printed or emailed), press releases, TV or radio advertisements, participation in trade shows and sector events, engagement through building owner and manager associations, and business referrals.

### Cost structure

ESCOs can estimate costs after defining key resources, activities, and partnerships. The business model in Table 5 is cost-driven, built around a low-cost structure. Success depends on using the most economical resources that still achieve the desired energy savings. High-cost activities may threaten the commercial viability of the service.

Specifically, costs to consider include the purchase, installation, and financing of energy-efficient equipment; operation and maintenance of installed systems; fees for consultants and contractors; ESCO personnel costs; and expenses associated with measuring and verifying energy savings.

An example of a typical retrofit cost structure is provided in Table 6. This case refers to the retrofit of an office building ( SOCIETY OF CHARTERED SURVEYORS IRELAND, 2025). In addition to retrofit costs, ESCO remuneration typically includes a 4–6% service and risk fee (depending on project size), a 5–10% contingency for unforeseen conditions, 6–8% for construction management and site supervision, 4–8% for design services (including technical drawings and specifications), 0–1% for an investment-grade audit, and 0–1% for measurement and verification of energy savings ( Navitas, 2020).

**Table 6.** Example of commercial building retrofit cost structure (office building).

Cost structure	Description	Percentage	Relevance with EPC
Demolitions	Costs associated with removing existing building elements to prepare for the retrofit. This includes removal of partitions, ceilings, old finishes, non-structural walls, obsolete mechanical and electrical installations, and any hazardous material abatement.	7.5 %	Only the demolition costs required to install energy-efficient systems (e.g., removing old HVAC or lighting systems) are relevant with the EPC.
Structural works	Expenses for reinforcing or modifying the building’s structural framework. This includes load-bearing walls, columns, beams, floor slabs, foundations, and structural repairs necessary to support the retrofit.	8.8 %	Only costs associated with structural modifications required to accommodate energy-efficiency upgrades, like supporting new HVAC equipment or solar panels are EPC-eligible.
External enclosures	Costs for upgrading the building envelope. Includes façades, external walls, windows, doors, roof, insulation, cladding, and any external shading devices aimed at improving energy efficiency and weatherproofing.	18.7 %	Upgrading façades, insulation, windows, and roof works are core interventions for improving building energy performance (thermal efficiency, air tightness) and therefore highly relevant with the EPC.
Works on the internal finishes and completions	Costs for refurbishing interior spaces after major structural and services works. Includes flooring, ceilings, wall finishes, paint, joinery, cabinetry, partitions, and fit-out items required for occupancy.	8.1 %	These works are only relevant with EPC if interior changes are needed to support energy-efficient systems (e.g., ceiling modifications for lighting or HVAC). Cosmetic finishes not tied to energy savings are generally not included in the EPC.
Works associated with services	Costs for mechanical, electrical, and plumbing systems. Includes heating, ventilation, air conditioning (HVAC), lighting, power distribution, fire protection, security, building automation, and controls. This category is usually the largest single cost component.	46.6 %	These costs represent the primary EPC focus and include HVAC upgrades, efficient lighting, building automation, metering, and renewable energy systems, which all directly contribute to energy savings.
Preliminaries	Costs that are project overheads and indirect construction costs. Includes site setup, scaffolding, temporary works, project management, training, permits, insurance, health & safety compliance, and any temporary facilities required during the retrofit.	10.3 %	Project management, site setup, and safety costs are included as part of the EPC project costs only to the extent they are relevant with the installation of energy efficiency measures.

### Revenue streams

Customers are willing to pay for improved energy efficiency, and some are motivated by the desire to reduce their environmental impact. Others may pay for enhanced indoor comfort, such as better thermal conditions. The pricing mechanism for the service should be based on a transparent and reliable estimate of achievable energy savings, as well as on whether the ESCO guarantees a specific level of savings or shares the savings according to predetermined percentages.

The main revenue stream for the ESCO comes from the share of energy cost savings realized by clients, primarily tenants and building owners if they occupy part of the property, as well as from fees for operation and maintenance services. An additional revenue stream may arise from equipment rental if the ESCO owns the equipment and leases it to the client for a fixed monthly fee. All revenues are collected through the service fee.

***4.4.2 Value proposition***

This section presents the development of the value proposition for the smart energy service targeting the commercial rented sector, applying the Value Proposition Canvas introduced in Section 2.4. The results of this analysis are summarized in Table 7.

**Table 7.** Value proposition of the proposed Business Model and its matching with customer-side requirements.

PRODUCT			CUSTOMER	
**Benefits** • Delivers energy efficiency gains and energy cost reductions to clients that occupy rented commercial properties. • Minimizes the performance risks for the client by providing an adequate level of guaranteed energy savings. • Provides a comprehensive service encompassing selection of energy efficiency measures, their installation, optimised system operation and maintenance, measurement and verification of energy savings. **Features** The Smart Energy Service will: 1. Design a building retrofit and building systems upgrades in collaboration with the client. 2. Implement, monitor and optimise selected energy efficiency measures. 3. Measure and verify the energy savings periodically (e.g. monthly or quarterly). 4. Provide a compensation to the clients if the contractual guaranteed energy savings are not achieved. 5. Reward the building owner proportionally to the savings achieved by the renters.	**Experience** ESCO’s clients gain certainty about the energy savings that will be achieved because the ESCO bears the performance risk of the project. Building owners are incentivised in taking the responsibility for an investment in energy efficiency because they are rewarded by the ESCO depending on the amount of savings achieved by the renters. The ESCO acts as a one-stop shop providing a comprehensive service, that does not require building owners and renters to liaise with multiple contractors. The ESCO takes the responsibility not only to install equipment and energy efficiency measures, but also to maintain them and operate them in an optimised manner.	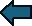	**Wants** Building owners want: 1) Renovate their buildings to increase their value on the rental market. 2) A revenue stream from an energy efficiency project. 3) Assistance with project financing, or identification of available grants that can support the purchase/installation of suitable energy efficiency measures. 4) Select and install affordable energy efficiency measures with a short payback time. 5) An energy performance contract (EPC) of not too long duration to maximise the rewards from the installed energy efficiency measures and mitigate the risk that some renters would leave, making the EPC no longer sustainable. Renters want: 1) Rent a renovated and comfortable property. 2) Increase their energy efficiency. **Needs** • A detailed *energy audit* to accurately evaluate the expected energy savings and the ability of the selected energy efficiency measures in achieving them. • To minimize the *technology risks* due to improper system design, improper equipment selection, unexpected equipment deterioration. • *Training* to operate the newly installed equipment according to the prescribed operation schedule and optimal control strategy and procedures. • A flexible *EPC contractual template* including clauses enabling 1) a system monitoring and optimisation period; 2) the building owner to obtain a reward proportional to the energy savings achieved by the renters.	**Fears** The energy efficiency measures are not affordable, or their payback time is too long. The expected energy savings will not be achieved for a variety of reasons such as their inaccurate estimation, low quality of system operation and maintenance, system performance degradation due to equipment wear and tear and operation parameter variations. Renters do not contribute to the energy efficiency investment, whereas the energy savings that they would achieve cannot be easily shared with the building owner, who takes the responsibility for the investment (split incentive issue).
**Product** Smart Energy Service for Commercial Rented Properties.	**Ideal customer** Building owners and renters of commercial rented units.		**Substitutes** The main substitute is the traditional method, also known as Design–bid–build or Design–tender, in which the building owner contracts with separate Companies and Consultants for the design and construction of an energy efficiency project.	

The proposed smart energy service enhances the value and green image of a property through the installation of energy efficiency measures and BMS upgrades. It also provides non-energy benefits, such as improved indoor comfort (e.g., thermal comfort and air quality) and property renovation. The monthly service fee paid by tenants (and in some cases by building owners) delivers value to the ESCO implementing the service.

Customer benefits include reduced energy bills, better thermal comfort, improved indoor air quality, decreased water waste, and other environmental gains. The value of the service is reinforced by the ESCO’s responsibility for the installation, commissioning, monitoring, and ongoing maintenance of equipment, devices, and systems, as well as the provision of guaranteed or shared energy savings.

Products that typically create value across customer segments include heat pumps, photovoltaic panels, LED lighting, building management systems, and smart building controls. Relevant sub-services include equipment installation or replacement, maintenance, and measurement and verification of energy savings.

Finally, the channels section describes how the ESCO communicates with and reaches its customer segments to effectively deliver this value proposition.

**4.4.2.1 Product or service**

**Benefits**

The service delivers value to clients occupying rented commercial properties by providing energy efficiency gains and reductions in energy costs. It reduces performance risks by guaranteeing a minimum level of energy savings. Another key benefit is the ‘one-stop shop’ approach, where a single contractor, the ESCO or energy efficiency provider, offers an integrated suite of services, including the selection and installation of energy efficiency measures, optimized system operation and maintenance, and measurement and verification of energy savings.

**Features**

The proposed smart energy service includes designing building retrofits and system upgrades tailored to the client’s requirements, implementing, monitoring, and optimizing selected energy efficiency measures, and periodically measuring and verifying the energy savings achieved (e.g., monthly or quarterly). Additional features include compensating clients if the guaranteed energy savings outlined in the energy performance contract are not met and providing a reward to the building owner proportional to the savings achieved by tenants. This latter feature is a key innovation of the service, effectively addressing the split-incentive issue in the commercial rented sector ( De Tommasi *et al.*, 2024).

**Experience**

Customers of the proposed smart energy service are likely to experience positive feelings due to the certainty of achieving energy savings, as the ESCO assumes the performance risk of the project ( Lee *et al.*, 2015). Building owners gain motivation and confidence to invest in energy efficiency, knowing they will be rewarded proportionally to the savings achieved by tenants. Both building owners and tenants benefit from reduced coordination efforts, as the ESCO acts as a one-stop shop, providing a comprehensive service. The ESCO is responsible for installing and maintaining energy efficiency measures and equipment, and for enabling clients to operate them optimally, ensuring that planned energy savings are realized.

**4.4.2.2 Customer**

**Wants**

Building owners aim to renovate their properties to increase their rental market value. They seek a revenue stream from energy efficiency projects, as there is little incentive to invest without financial returns. They also require support with project financing, including identifying grants to fund the purchase and installation of suitable energy efficiency measures. Additionally, building owners need specialized services to select and install cost-effective measures with short payback periods. They prefer an energy performance contract (EPC) of an appropriate duration, long enough to capture rewards from the installed measures but not so long that tenant turnover jeopardizes the contract’s sustainability.

Renters, on the other hand, desire renovated, comfortable properties and improved energy efficiency, provided that these objectives are achievable.

**Needs**

In the context of energy efficiency in commercial rented buildings, customers require detailed energy audits to accurately identify measures that can deliver significant energy savings and to estimate the potential savings. They need a service that minimizes technology risks, such as improper system design, incorrect equipment selection, or unexpected equipment deterioration ( Lee *et al.*, 2015). Appropriate training is also essential, enabling clients to operate newly installed equipment according to the optimal schedules and control strategies defined by the ESCO. Additionally, customers benefit from a flexible EPC contractual template that allows for system monitoring and optimization and provides the building owner with a reward proportional to the energy savings achieved by tenants ( De Tommasi *et al.*, 2024).

**Fears**

In the context of smart energy services for the commercial rented sector, customers may have several concerns. They may fear that energy efficiency measures are unaffordable or have excessively long payback periods. Another common concern is that expected energy savings may not be realized due to factors such as inaccurate estimations, poor system operation and maintenance, equipment wear and tear, or variations in operating parameters. An additional challenge arises from the rental nature of the property: tenants may not invest in energy efficiency because they do not own the premises, while building owners bear the investment cost but cannot easily share in the energy savings achieved by tenants. This challenge is known as the split-incentive issue, as discussed in Section 2.1 ( Castellazzi *et al.*, 2017; Ring *et al.*, August 2022).

## 5. Learnings from the SmartSPIN pilot-sites in Ireland, Spain and Greece

In this section, the main learnings obtained from the pilot-sites of the SmartSPIN project in Ireland, Spain and Greece are summarised, highlighting the factors identified as barriers or drivers for the implementation of the proposed smart energy service ( De Tommasi *et al.*, 2025).

The experience of the SmartSPIN pilot site in Ireland showed that introducing energy performance contracting (EPC) faced mostly practical and organisational challenges, rather than technical ones. The main issue was that private-sector partners were not very familiar with the EPC model. Working with intermediaries, such as the property management agent and facility management company, also required a lot of time and coordination. Because the tenants’ head offices were located elsewhere, it took longer to get approvals for participation and to share energy data. The final contract used at the site was designed to be simple and flexible, allowing short-term testing of low-cost measures before investing in larger, more expensive actions. Overall, the Irish pilot showed that stakeholder caution and divided responsibilities may delay the implementation of an EPC in an office building.

At the Greek pilot site (an office building with several tenants that used to be an incubator) feedback on the smart energy service was positive. Both the landlord and tenants saw its potential to solve the split incentive problem and to share energy savings more fairly. However, the low development of the local energy services market may influence and delay stakeholders’ decision making. According to the participants, commercial ESCOs in Greece usually focus on public-sector projects and are waiting for clearer national guidance from the Ministry of Energy before entering the private sector. The building owner carried out some independent upgrades, such as better insulation and façade improvements, before considering an EPC. In this case, market conditions emerged as a possible barrier rather than a lack of interest.

At the Spanish pilot sites (Plenilunio and La Gavia shopping centres), the SmartSPIN business model was seen as effective in addressing the split incentive issue in multi-tenant buildings. Stakeholders valued the transparency and trust created by SmartSPIN’s data analytics and its system for measuring and verifying results. However, using a performance-based contract requires careful management of the complex relationships between landlords, tenants, and the ESCO. The landlord’s internal policy of directly funding energy efficiency projects may reduce the appeal of using an EPC. The business case also needs to be clearly explained, for example using NPV calculations (as it is shown in this paper). In addition, many tenants were multinational companies, which added complexity since energy decisions were made at corporate lever, rather than at local level. The digital monitoring and reporting tools are a driver for the implementation of a smart energy service and help improve energy management.

When comparing the three pilot sites, a few key lessons emerge. The Spanish sites showed that a more advanced and digital environment is a driver for the implementation of a smart energy service, whereas internal company policies and complexity arising from engagement and coordination of a high number of tenants may slow down adoption of third-party performance contracts. The Irish site showed that even when there is interest, low familiarity with EPCs and complex management structures can cause delays. The Greek site showed that openness among landlords and tenants helps drive the EPC implementation, but perceived gaps in the maturity of the energy services market and regulatory framework delay decision-making.

## 6. Conclusions

This paper has used a quantitative model that enables the evaluation of the Net-Present-Value of an energy efficiency project for building owner, energy service company (service provider) and renters, to highlight how the energy cost savings, energy prices, investment costs, operation and maintenance costs and the rebound effect of the energy consumption of the renters, may influence the decision-making in relation to the feasibility of an energy performance contract in a rented commercial property, as well as the contract duration. The analysis showed that the higher the capital investment made by the ESCO, the longer the contract duration proposed by the ESCO. The decision making in relation to contractual agreement can be greatly facilitated if transparent calculations are shown to the potential customers, as shown in this paper. Furthermore, it has been highlighted that a suitable arrangement to overcome the barrier of the long duration of the Energy Performance Contracts (EPC) in the commercial rented sector is to foster the sharing of the initial capital investment in energy efficiency measures and equipment between ESCO and customer (building owner), such that the EPC contract duration can be reduced because of the customer’s direct contribution to the investment, while the ESCO would still contribute to it (when the client cannot fully bear the investment).

Moreover, the review presented in this paper shows that, when renters are not given sufficient economic incentive to save energy, they might use even more energy after the energy retrofit, reducing the total energy savings and the value of their part taken by the ESCO. A sensitivity analysis was performed with respect to the two parameters used to model the rebound effect (i.e., the risk tolerance and the maximum rebound effect) which showed the importance for an ESCO to estimate as accurately as possible such effect. Indeed, in presence of a high rebound effect, the energy savings will be lower than expected and the ESCO will need a longer contract duration to reduce the risk of economic losses and maximize the chances to achieve the desired profit.

The underlying business model for a smart energy service suitable for the commercial rented sector has been fully characterised and analysed using the business model CANVAS table. The following aspects of relevance for the businesses that want to implement the service: key partners, key activities, key resources, cost structure, value proposition, customer relationship, channels, customer segment, revenue streams, were highlighted. Furthermore, the paper has investigated how the considered smart energy service can create value for the customers, designing its value proposition using the Value Proposition CANVAS templates.

The study provides several important *benefits*. First, it establishes a direct connection between the technical-economic assessment of a smart energy service suitable for the commercial rented sector (based on energy performance contracting) and the Business Model Canvas and Value Proposition Canvas frameworks. This novel integration bridges the gap between the quantitative and qualitative dimensions that must be considered to successfully implement such a service. Second, the study delivers actionable insights for key stakeholders (building owners, tenants, and energy service companies) by clarifying contractual arrangements such as contract duration, the service fee to be paid by clients to the ESCO, and the appropriate sharing of energy savings between building owners and tenants. These arrangements are essential to overcoming the split-incentive barrier in rented commercial properties. Finally, the integration of quantitative modelling with business model frameworks creates a replicable methodology that can be applied in future studies and adapted to other sectors, thereby supporting both academic research and the practical implementation of sustainable energy services.

The main *limitation* of the proposed study is that it did not address the estimation of the rebound-effect parameters ρ (risk tolerance) and φ (maximum rebound) using field data, due to the very limited availability of such data. Although sensitivity analyses were conducted, an empirical practical approach for model parameters estimation needs to be developed. Future research will focus on developing a methodology to empirically estimate these parameters, drawing on approaches such as controlled field trials, statistical modelling of energy use, literature-based calibration, discrete choice surveys, and analysis of real contract uptake and energy consumption data.

## 7. Replicability of the study

To replicate the study described in this paper, it is recommended to start with a thorough review of the literature to determine the financial models which apply to energy performance contracting in the commercial rented sector. Such models should be thoroughly analysed to understand the rational behaviour of stakeholders (landlords, tenants and ESCOs) using the lens of utility maximization and find out how each of them acts in their own best interest, based on the economic value of the service for them, incentives (if any), constraints, attitude to consume energy, risk propensity. This analysis should lead to a better understanding of how the stakeholders interact with each other and of the decision-making processes that may lead to the signature of an energy performance contract. After reviewing and analysing the financial models, the research team will organise a brainstorming session, where they will fill in the templates that were selected to develop the business model (e.g. CANVAS). They will consider the perspective and the requirements of the stakeholders, as obtained from previously held interviews and/or focus groups. After completing a draft of the business model, one or more members of the research team will improve its representation, making it more compact, clear and immediate to read, e.g., removing repetitions or marginally relevant entries in the CANVAS tables, while making sure that the main aspects required by each section of the template are addressed.

## Reporting guidelines

This paper provides all the details of the materials and methods used so that the work can be repeated by others. These details include: 1. equations used for the quantitative analysis of the business model and the related decision making undertaken by the stakeholders; 2. the CANVAS Business Model & Value Proposition templates that have been used for the design of the proposed business model; 3. A comprehensive list of references.

## Ethics and consent

Ethical approval and consent were not required.

## Data Availability

No data associated with this article.

## Appendix

In this appendix, we discuss and compare qualitatively alternative forms for the rebound effect multiplier ( 13). These alternative models are the exponential model, logistic model, stepwise model (with n-steps), threshold-based model.

### Exponential model

The exponential model ( 13) describes a gradual, continuous increase in the rebound effect multiplier as the landlord captures a larger share of energy savings. This model is realistic for describing the behaviour of tenants whose energy use responds progressively to the increase of the energy savings share taken by the landlord.

### Logistic model

Re⁡log⁡(θ)=1+β1+exp⁡(αθ0)−β1+exp⁡[−α(θ−θ0)](22)

The logistic model ( 22) produces an S-shaped curve, where initially Re decreases from 1 to a value lower than 1, as *θ* increases from 0 to 1. Variations are initially slow, then faster, and finally slow again (saturation). The logistic form models the behaviour of tenants who initially respond little but eventually increase energy use more significantly as the landlord energy savings share increases, and after that do not respond significantly to further increases of landlord’s share.

### Stepwise model with n steps

Re⁡step(θ)={1,θ<θ11+α−1n−1,θ1≤θ<θ21+2α−1n−1,θ2≤θ<θ3⋮α,θn−1≤θ≤1(23)

With the stepwise model ( 23), Re decreases in discrete jumps at thresholds *θ _i_* from 1 to *α* < 1. It is particularly suitable to model the behaviour of tenants who adjust their energy use at specific share thresholds rather than continuously.

### Threshold-based model

Re⁡th(θ)={1,θ<θt1+(α−1)θ−θt1−θt,θ≥θt(24)

With the threshold-based model ( 24), Re remains at *α _1_* until the threshold is reached *θ _t_*, then varies linearly to *α* < 1. It is suitable to model the behaviour of tenants that ignore small landlord shares and only respond once the share of energy savings taken by the landlord becomes significant.
