# Supplementary material for: A smart energy service for the commercial rented sector: decision making points and business model considering the learnings from the pilot sites of the SmartSPIN project in Ireland, Spain and Greece
Source: Open Res Eur. 2025 Dec 16;5:39. Originally published 2025 Feb 4. [Version 2] doi: 10.12688/openreseurope.18851.2 (PMC13632805; doi:10.12688/openreseurope.18851.2)
Supplement: Peer review history

# Reviewer report: Reviewer response for version 2

**DOI:** 10.21956/openreseurope.23899.r66192

**Published:** 2026-01-07

**Authors:** Robert C. Moehler

**Affiliations:** 1 The University of Melbourne, Melbourne, Victoria, Australia

My comments have been addressed. Minor fine tuning of some of the claims "no prior studies..." could be epistemologically soundly rephrased into providing new insights by applying the business model in the context.... Otherwise well done the study reads well.

Is the study design appropriate and does the work have academic merit?

Yes

Is the work clearly and accurately presented and does it cite the current literature?

Yes

If applicable, is the statistical analysis and its interpretation appropriate?

Partly

Are all the source data underlying the results available to ensure full reproducibility?

Partly

Are the conclusions drawn adequately supported by the results?

Yes

Are sufficient details of methods and analysis provided to allow replication by others?

Yes

Reviewer Expertise:

Business Model Innovation

I confirm that I have read this submission and believe that I have an appropriate level of expertise to confirm that it is of an acceptable scientific standard.

# Reviewer report: Reviewer response for version 2

**DOI:** 10.21956/openreseurope.23899.r66193

**Published:** 2026-01-06

**Authors:** Marcele Elisa Fontana

**Affiliations:** 1 Federal University of Pernambuco, Recife, Brazil

I still find the number of subsections excessive, particularly in the introduction, where **continuous text** would be more appropriate. Furthermore, explaining each individual subsection is unnecessary; the authors should briefly present only the main sections.

Is the study design appropriate and does the work have academic merit?

Yes

Is the work clearly and accurately presented and does it cite the current literature?

Yes

If applicable, is the statistical analysis and its interpretation appropriate?

Not applicable

Are all the source data underlying the results available to ensure full reproducibility?

Yes

Are the conclusions drawn adequately supported by the results?

Yes

Are sufficient details of methods and analysis provided to allow replication by others?

Partly

Reviewer Expertise:

The article is interesting and relevant.

I confirm that I have read this submission and believe that I have an appropriate level of expertise to confirm that it is of an acceptable scientific standard.

# Reviewer report: Reviewer response for version 2

**DOI:** 10.21956/openreseurope.23899.r66194

**Published:** 2026-01-02

**Authors:** Charalampos Alexopoulos

**Affiliations:** 1 University of the Aegean, Samos, Greece

I confirm that the authors have adequately addressed all of my comments. The new version is ready to be indexed.

Is the study design appropriate and does the work have academic merit?

Yes

Is the work clearly and accurately presented and does it cite the current literature?

Yes

If applicable, is the statistical analysis and its interpretation appropriate?

Partly

Are all the source data underlying the results available to ensure full reproducibility?

Partly

Are the conclusions drawn adequately supported by the results?

Yes

Are sufficient details of methods and analysis provided to allow replication by others?

Partly

Reviewer Expertise:

Digital Governance, Open Data Governance, Semantic Interoperability, Services desing and development

I confirm that I have read this submission and believe that I have an appropriate level of expertise to confirm that it is of an acceptable scientific standard.

# Reviewer report: Reviewer response for version 1

**DOI:** 10.21956/openreseurope.20401.r54206

**Published:** 2025-06-03

**Authors:** Robert C. Moehler

**Affiliations:** 1 The University of Melbourne, Melbourne, Victoria, Australia

1. Although the authors have presented rebound effect parameters (φ and ρ), no sensitivity analysis has been provided in detail to evaluate how NPV outcomes change for various stakeholder groups at varying contract lengths. Sensitivity tables or tornado diagrams need to be presented.

 2. The degradation function f(t) decreases linearly from 1 to 0, yet there is no technical or empirical justification provided for such a choice. Include references or real degradation profiles from HVAC, LED, or BMS research for validation.

 3. Stochastic modelling based on GBM (Equations 13–15) is said to be "validated" using QuantPy, but there is no numerical or graphical comparison (e.g., standard deviation bands, simulation replicates) shown. Include at least 30 Monte Carlo paths and compare against analytical results.

 4. The payment mechanism of the service, a key driver of cash flow, is not properly defined in terms of indexation on energy prices or inflation. Clarify if the ESCO fees are fixed, performance-based, or variable, and illustrate this explicitly in the NPV formula.

 5. The financial equations provided do not mention units, which is confusing to readers working with capital expenditure, cost savings, and service charges. Provide a table of symbols, units, and typical values used.

 6. The assumption that tenants make service fee payments on a continuous basis overlooks potential for default, vacancy, or lease rollover risk. Initiate a discussion on credit risk or dropout risk and its implications for NPV and contract feasibility.

 7. The model is very sensitive to rebound effect parameters ρ (risk tolerance) and φ (max rebound), yet no empirical practical approach is provided for their estimation. Recommend conducting survey-based or behavioural experiments to ground these values in.

 8. Although the Business Model Canvas (Table 1) mentions cost factors, it lacks quantitative detail (e.g., % of capital, O&M, training). Incorporate an example of a typical retrofit case cost structure to provide greater transparency.

 9. The report mentions pilot sites located in Ireland, Greece, and Spain; yet it fails to make comparative quantitative analysis or observations from the differences in regulation, building features, or tenant habits in these countries.

 10. Equation (11) introduces an exponential term without discussion of its behavioural realism. Alternative models (e.g., logistic, stepwise, or threshold-based) might better capture empirical rebound behaviour. Include a comparison. 11. NPV calculations are presented based on multiple stakeholder flows, yet no graphical cash flow timeline (by stakeholder) is provided. Incorporate cash flow diagrams or Sankey plots to illustrate financial exchanges over the project time frame.

Is the study design appropriate and does the work have academic merit?

Yes

Is the work clearly and accurately presented and does it cite the current literature?

Yes

If applicable, is the statistical analysis and its interpretation appropriate?

Partly

Are all the source data underlying the results available to ensure full reproducibility?

Partly

Are the conclusions drawn adequately supported by the results?

Yes

Are sufficient details of methods and analysis provided to allow replication by others?

Yes

Reviewer Expertise:

Business Model Innovation

I confirm that I have read this submission and believe that I have an appropriate level of expertise to confirm that it is of an acceptable scientific standard, however I have significant reservations, as outlined above.

# Response

**Published:** 2025-12-18

**Authors:** Luciano De Tommasi

**Affiliations:** International Energy Research Center, Ireland

**Dear Reviewer,** **Thank you for your detailed and constructive comments. The version 1 of the manuscript was carefully revised to address all of your comments. Point-by-point responses to each comment are provided below.**

1. Although the authors have presented rebound effect parameters (φ and ρ), no sensitivity analysis has been provided in detail to evaluate how NPV outcomes change for various stakeholder groups at varying contract lengths. Sensitivity tables or tornado diagrams need to be presented.

Thank you for your comment. Sensitivity analysis to evaluate how NPV change for various stakeholders’ groups characterised by different rebound effect parameters ( *φ*  and *ρ* ) at varying contract lengths (14, 15, 16, 17 years) has been added to the revised version of the paper. The results of the sensitivity analysis are reported in Table 2, 3 and 4, respectively for the ESCO, building owner and renters. 2. The degradation function f(t) decreases linearly from 1 to 0, yet there is no technical or empirical justification provided for such a choice. Include references or real degradation profiles from HVAC, LED, or BMS research for validation. Thank you for your comment. A detailed analysis of real degradation profiles for energy efficiency equipment is a very interesting subject that is out of the scope of our paper. The degradation function used has been added in equation (11) in the revised version of the paper. We used the same function used in the following reference: LU, Yujie; ZHANG, Nan; CHEN, Jiayu. A behavior-based decision-making model for energy performance contracting in building retrofit. *Energy and Buildings*, 2017, 156: 315-326. The function decreases to zero following to a logarithmic expression.

 3. Stochastic modelling based on GBM (Equations 13–15) is said to be "validated" using QuantPy, but there is no numerical or graphical comparison (e.g., standard deviation bands, simulation replicates) shown. Include at least 30 Monte Carlo paths and compare against analytical results. Thank you for your comment. A numerical comparison between the outcomes of stochastic GBM processes and their analytical approximations has been included in the revised version of the paper considering 30 Monte Carlo run. Mean and standard deviation of the Root Relative Square Error (RRSE) have been included in sections 3.2.2, 3.2.3, 3.2.4  (respectively for the energy savings potential *K* *t* , the future energy price *P* *E* *(t)* , and the operation and maintenance costs *I* *OM* *t* ) highlighting its excellent approximation.   In section 3.2.2 the following text was added: “The accuracy of the approximation ( 12) has been validated comparing it against the outputs of an actual Geometric Brownian Motion stochastic process ( QUANTPY, 2024), using parameters included in the case study in Table 1. Thirty Monte Carlo runs were performed, obtaining a mean Root Relative Square Error (RRSE) of 0.10986 with a standard deviation of 0.05672, which proves the accuracy of the approximation.” In section 3.2.3 the following text was added: “The accuracy of the approximation ( 15) has been validated comparing it against the outputs of an actual Geometric Brownian Motion stochastic process ( QuantPy, 2024), using parameters included in the case study in Table 1. Thirty Monte Carlo runs were performed, obtaining a mean Root Relative Square Error (RRSE) of 0.01467 with a standard deviation of 0.00862, which prove the accuracy of the approximation.” In section 3.2.4 the following text was added: “The accuracy of the approximation ( 16) has been validated comparing it against the outputs of an actual Geometric Brownian Motion stochastic process ( QuantPy, 2024), using parameters included in the case study in Table 1. Thirty Monte Carlo runs were performed, obtaining a mean Root Relative Square Error (RRSE) of 0.03718 with a standard deviation of 0.01536, which prove the accuracy of the approximation.”

 4. The payment mechanism of the service, a key driver of cash flow, is not properly defined in terms of indexation on energy prices or inflation. Clarify if the ESCO fees are fixed, performance-based, or variable, and illustrate this explicitly in the NPV formula.

 Thank you for your comment. The ESCO fees are performance-based, as shown in the NPV formulations (Equations 4–6). The term *G+*max *⁡* *[0,* *β* *(* *R* *(* *t* *)-* *G* *)]*  indicates that the ESCO guarantees energy savings of *G*  for its clients (shared between the building owner and renters) and shares a fraction *β*  of any additional savings beyond this guaranteed threshold with its clients. This structure results in a variable total fee *S*  paid by the renters to the ESCO (section 4.2.3). When the actual energy savings exceed the guaranteed value *G* , the ESCO shares the surplus savings with its clients, thereby reducing the net total fee *S* . The NPV calculations already incorporate projected variations in energy prices (Section 3.2.3) and operation and maintenance costs (Section 3.2.4). Consequently, the service fee agreed between the ESCO, and the renters ensures that the desired NPVs are achieved by the Parties while accounting for future fluctuations in both energy prices and O&M costs.

 5. The financial equations provided do not mention units, which is confusing to readers working with capital expenditure, cost savings, and service charges. Provide a table of symbols, units, and typical values used. Thank you for your comment. Table 1 including symbols, units and typical values used has been added.

 6. The assumption that tenants make service fee payments on a continuous basis overlooks potential for default, vacancy, or lease rollover risk. Initiate a discussion on credit risk or dropout risk and its implications for NPV and contract feasibility.

 Thank you for your comment. To address your comment, the following text has been added in section 4.2.1: “It is worthwhile to note that, if a tenant fails to pay the service fee to the ESCO due to financial difficulties, or if a space remains unleased, the ESCO may experience reduced cash flows. Similarly, when a lease expires and the tenant moves out, the ESCO may lose the associated revenue stream unless a new tenant immediately occupies the space. To account for these scenarios, the expected cash flow for the ESCO can be adjusted by the probability of payment, which results in a lower net present value compared to a situation with guaranteed and uninterrupted payments. Higher uncertainty or risk may also require the ESCO to increase service fees, potentially affecting project feasibility. Contracts can include provisions to mitigate these risks and ensure that the EPC remains financially sustainable and attractive for all parties involved. For example, guarantees from the building owner can shift credit risk by ensuring that the owner covers the ESCO’s fees if a tenant fails to pay, allowing the ESCO to receive expected payments even in case of default. Insurance policies can also protect the ESCO by compensating for missed payments due to tenant insolvency, reducing the impact of defaults on project NPV. Reserve funds, created by setting aside a portion of collected fees, provide a buffer to cover periods of non-payment or vacancy, enabling the ESCO to continue operations during temporary shortfalls. Minimum guaranteed payments establish a baseline revenue for the ESCO regardless of tenant occupancy or energy savings performance, ensuring that essential costs are covered. Finally, fees tied to occupancy adjust payments based on the number of tenants in the building, increasing when occupancy is high and decreasing when it is low, which aligns the ESCO’s remuneration with actual risk and performance.”

 7. The model is very sensitive to rebound effect parameters ρ (risk tolerance) and φ (max rebound), yet no empirical practical approach is provided for their estimation. Recommend conducting survey-based or behavioural experiments to ground these values in. Thank you for your comment. Empirical estimation of rebound effect parameters ρ (risk tolerance) and φ (max rebound) through survey-based or behavioural experiments was out of the scope of our project. This is a limitation of the presented study, which aimed to perform a sensitivity analysis to investigate the effect of variations of such parameters. The following text has been added to the conclusions to clarify the point raised by the reviewer: “The main *limitation* of the proposed study is that it did not address the estimation of the rebound-effect parameters ρ (risk tolerance) and φ (maximum rebound) using field data, due to the very limited availability of such data. Although sensitivity analyses were conducted, an empirical practical approach for model parameters estimation needs to be developed. Future research will focus on developing a methodology to empirically estimate these parameters, drawing on approaches such as controlled field trials, statistical modelling of energy use, literature-based calibration, discrete choice surveys, and analysis of real contract uptake and energy consumption data.”

 8. Although the Business Model Canvas (Table 1) mentions cost factors, it lacks quantitative detail (e.g., % of capital, O&M, training). Incorporate an example of a typical retrofit case cost structure to provide greater transparency. Thanks for your comment. The following text has been added in the section “Cost structure” of section 4.4.1.

 “An example of a typical retrofit cost structure is provided in Table 6. This case refers to the retrofit of an office building (SOCIETY OF CHARTERED SURVEYORS IRELAND, 2025). In addition to retrofit costs, ESCO remuneration typically includes a 4–6% service and risk fee (depending on project size), a 5–10% contingency for unforeseen conditions, 6–8% for construction management and site supervision, 4–8% for design services (including technical drawings and specifications), 0–1% for an investment-grade audit, and 0–1% for measurement and verification of energy savings (Navitas, 2020).”  Table 6: Example of commercial building retrofit cost structure (office building) – was added. The following references have been added to the reference list: NAVITAS. (2020, September). *Figure 1: The process to implement a guaranteed energy savings performance contract* [PDF]. Available at: https://media.navitas.us.com/wp-content/uploads/2020/09/18145329/04-ESCO-Figure-1.pdf [Accessed on December 3 ^rd^ 2025]. SOCIETY OF CHARTERED SURVEYORS IRELAND (2025). The Real Cost of Retrofitting Offices Report 2025: Analysis of office block retrofit costs and viability. Dublin: SCSI. Available at: https://www.scsi.ie/wp-content/uploads/2025/03/SCSI_RealCostRenovatingOfficeBlocks_2025.pdf [Accessed on October 26 ^th^ 2025]. 9. The report mentions pilot sites located in Ireland, Greece, and Spain; yet it fails to make comparative quantitative analysis or observations from the differences in regulation, building features, or tenant habits in these countries.

 Thank you for your comment. Section 5, “Learnings from the SmartSPIN Pilot Sites in Ireland, Spain, and Greece,” has been added in response to your comment. It presents observations, including stakeholder behaviour, while taking into account contextual factors such as building type (office or shopping centre) and the maturity of the local ESCO market. 

 10. Equation (11) introduces an exponential term without discussion of its behavioural realism. Alternative models (e.g., logistic, stepwise, or threshold-based) might better capture empirical rebound behaviour. Include a comparison. Thank you for your comment. The following comparison between rebound effect models has been added in the appendix of the paper. Exponential Model The exponential model (12) describes a gradual, continuous increase in the rebound effect multiplier as the landlord captures a larger share of energy savings. This model is realistic for describing the behaviour of tenants whose energy use responds progressively to the increase of the energy savings share taken by the landlord. Logistic Model - The logistic model (21) produces an S-shaped curve, where initially Re decreases from 1 to a value lower than 1, as *θ*  increases from 0 to 1. Variations are initially slow, then faster, and finally slow again (saturation). The logistic form models the behaviour of tenants who initially respond little but eventually increase energy use more significantly as the landlord energy savings share increases, and after that do not respond significantly to further increases of landlord’s share. Stepwise Model with n steps – With the stepwise model (22), Re decreases in discrete jumps at thresholds *θ*i  from 1 to *α* *<1* . It is particularly suitable to model the behaviour of tenants who adjust their energy use at specific share thresholds rather than continuously. Threshold-Based Model – With the threshold-based model (23), Re remains at *α*1  until the threshold *θ* *t*  is reached, then varies linearly to *α* *<1* . It is suitable to model the behaviour of tenants that ignore small landlord shares and only respond once the share of energy savings taken by the landlord becomes significant. 11. NPV calculations are presented based on multiple stakeholder flows, yet no graphical cash flow timeline (by stakeholder) is provided. Incorporate cash flow diagrams or Sankey plots to illustrate financial exchanges over the project time frame. Thank you for your comment. In the revised version of the paper, cash flow diagrams illustrating the financial exchanges have been added in Figure 5. The following text has been added to explain Figure 5: “Figure 5 shows the cash flow diagrams illustrating the financial exchanges over the project time frame for contract duration n = 15, *θ*=0.8 , *γ*=0.35 , *φ*=0.1 , *ρ*=-20  and other parameters included in Table 1. These cash-flows result in a NPV for ESCO, building owner and renters of € 1,313,971, € 1,476,850, and € 500,397 respectively. It can be noticed that the cash flow is negative for the ESCO and building owner at t = 0 as they perform the investment to install energy efficiency measures. The ESCO achieves a positive cash flow during the EPC contract (1≤ *t*≤ *n* ) and zero after the end of the contract ( *n*+1≤ *t*≤ *N* ). It can also be noticed that after the end of the contract, cash flows increase for the building owner and renters, as they no longer share energy savings with the ESCO. It can also be noted that at t = N the energy efficiency measures reach their end of life and no longer produce energy savings. The slightly negative cash flow for the building owner at t = N represents the O&M costs.”

# Reviewer report: Reviewer response for version 1

**DOI:** 10.21956/openreseurope.20401.r52503

**Published:** 2025-05-27

**Authors:** Charalampos Alexopoulos

**Affiliations:** 1 University of the Aegean, Samos, Greece

This paper presents an evaluation of the smart energy service for the commercial rented sector developed during the European Commission’s Horizon 2020 funded research project SmartSPIN – Smart energy services to solve the SPlit INcentive problem in the commercial rented sector – highlighting the key decision-making points related to energy performance contracting in rented commercial properties. The study utilizes quantitative models to evaluate the Net Present Value (NPV) of the energy efficiency project for each party (building owner, energy service provider, and renters), highlighting the variables most important for decision-making leading to contractual agreements for the smart energy service. The author presents the business model and value proposition canvas supporting the quantitative models. The methods have been implemented, and pilot cases have been evaluated through the involvement of project partners.

 Despite a clear study theme and problem formulation, there are some shortcomings that need to be addressed:

- There are unnecessarily very long sentences in the document; please see the abstract.
- The introduction is a bit prolonged. It could be better to mention the problem statement and the paper's contribution, and reduce the inclusion of non-essential content in the introduction. I did not understand why the introduction of blockchain is required in the introduction section.
- It would be good to break down the introduction into two sections (research background or literature review), whichever fits your needs.
- There are only 1–2 recent references in the introduction. Please try to bridge the research gap supported by recent literature and reduce the gaps among the studies.
- Some portions from the Research Context and Methodology section can easily be moved to the new section that you will create after trimming the introduction.
- Why is the business model being presented in the methodology section, when it was your main outcome?
- Figure 1 and Figure 2 seem quite similar; please try to explain or visualize their actual differences.
- In Figure 2, why are there two similar stages in the visualization with the same steps? Please try to mention the differences among the sub-steps as well.
- The methodology is overly complicated with mathematical models. It would be good to define it in an algorithmic way if possible.
- The results include different kinds of mathematical equations which were never introduced in the methodology. It would be good to dedicate a section to introduce these complicated elements.
- Overall, the study needs to be organized in a way that allows mathematical models and other results to be perceived easily by the readers.

Is the study design appropriate and does the work have academic merit?

Yes

Is the work clearly and accurately presented and does it cite the current literature?

Yes

If applicable, is the statistical analysis and its interpretation appropriate?

Partly

Are all the source data underlying the results available to ensure full reproducibility?

Partly

Are the conclusions drawn adequately supported by the results?

Yes

Are sufficient details of methods and analysis provided to allow replication by others?

Partly

Reviewer Expertise:

Digital Governance, Open Data Governance, Semantic Interoperability, Services desing and development

I confirm that I have read this submission and believe that I have an appropriate level of expertise to confirm that it is of an acceptable scientific standard, however I have significant reservations, as outlined above.

# Response

**Published:** 2025-12-18

**Authors:** Luciano De Tommasi

**Affiliations:** International Energy Research Center, Ireland

**Dear Reviewer,** **Thank you very much for your thoughtful and detailed comments, which have greatly helped to improve the quality of our manuscript. The paper has been carefully revised to address all your suggestions. Below, point-by-point responses to each of your comments are provided.**

 This paper presents an evaluation of the smart energy service for the commercial rented sector developed during the European Commission’s Horizon 2020 funded research project SmartSPIN – Smart energy services to solve the SPlit INcentive problem in the commercial rented sector – highlighting the key decision-making points related to energy performance contracting in rented commercial properties. The study utilizes quantitative models to evaluate the Net Present Value (NPV) of the energy efficiency project for each party (building owner, energy service provider, and renters), highlighting the variables most important for decision-making leading to contractual agreements for the smart energy service. The author presents the business model and value proposition canvas supporting the quantitative models. The methods have been implemented, and pilot cases have been evaluated through the involvement of project partners.

 Despite a clear study theme and problem formulation, there are some shortcomings that need to be addressed:

- There are unnecessarily very long sentences in the document; please see the abstract.

Thanks for your comment. The abstract and the introduction have been revised to remove long sentences.

- The introduction is a bit prolonged. It could be better to mention the problem statement and the paper's contribution and reduce the inclusion of non-essential content in the introduction. I did not understand why the introduction of blockchain is required in the introduction section.

Thank you for your comments. A focused section outlining the problem statement and the paper’s contributions has been added to the Introduction (section: 1.1 Problem statement and paper’s contribution). References to blockchain have been removed from this section to make it more concise and synthetic, as they were not essential.

- It would be good to break down the introduction into two sections (research background or literature review), whichever fits your needs.

Thanks for your comment. The introduction has now been broken down into three short sections: 1.1 Problem statement and paper’s contribution, 1.2 Research background, and 1.3 Objective, contributions and structure of the paper.

- There are only 1–2 recent references in the introduction. Please try to bridge the research gap supported by recent literature and reduce the gaps among the studies.

The introduction has been trimmed to make it more compact. Six recent references are included (paper published less than 5 years ago) in section 1.2. Overall, the article includes more than 50 references and does try to bridge research gaps among published studies. An objective of the paper is to bridge the gap between studies that develop an analytical model used to assess decision-making points of a smart energy service based on energy performance contracting, and the qualitative aspects of its business model.

- Some portions from the Research Context and Methodology section can easily be moved to the new section that you will create after trimming the introduction.

Thanks for your comment. Indeed, introduction was trimmed as you suggested. The Research Context and Methodology section was shortened separating into two sections (as suggested by another reviewer).

- Why is the business model being presented in the methodology section, when it was your main outcome?

Thank you for your comment. Indeed, the purpose of Section 3, titled “Methodology”, is not to present the business model (which is the main outcome of the paper, as correctly noted by the reviewer), but to introduce three aspects of the methodology, which are used to develop the business model:

- The high-level methodology to implement the proposed smart energy service (section 3.1), which consists in the steps that an ESCO will need to take to implement the proposed smart energy service in a commercial rented property.
- The *mathematical model* that is used to study the factors influencing the decision-making process in relation to Energy Performance contracting for commercial rented buildings (section 3.2). Title of section 3.2 has been changed into “ *Mathematical model used to investigate the factors influencing the decision-making process in relation to Energy Performance contracting for commercial rented buildings*” to clarify that the purpose of this section is to review the mathematical model used to assess the financial viability of the business model, and not to describe a part of the business model itself. The mathematical model  is used in section 4.2 to obtain insights about the proposed business model analysing a case study.
- The business model canvas (section 3.3), which is the main template used to develop the business model for the proposed smart energy service. The template is used in section 4.4.1 to develop the business model for the proposed smart energy service, which is the main outcome of the paper (alongside the other results included in section 4).
- The value proposition canvas (section 3.4), which is the template used to determine details of the value proposition of the proposed smart energy service ensuring that it is aligned with the needs of the customer. This template is used to develop the value proposition for the proposed smart energy service in section 4.4.2.

- Figure 1 and Figure 2 seem quite similar; please try to explain or visualize their actual differences.

Thank you for your comment. Figure 2 was updated in version 2 of the paper. Figure 1 and Figure 2 show two variants of the business model for the smart energy service developed in the SmartSPIN project. Figure 1 illustrates the case where the energy efficiency measures are financed by the building owner using their own sinking fund. Figure 2 illustrates the case where the energy efficiency measures are financed by the Energy Service Company through a loan arranged with a bank or lender. Captions of the two figures have been updated to highlight such differences between the two figures.

- In Figure 3, why are there two similar stages in the visualization with the same steps? Please try to mention the differences among the sub-steps as well.

Thank you for your comment. Indeed, the two implementation stages do include the same steps for the deployment of energy efficiency measures, which are: energy audit, planning, implementation, monitoring. However, the first stage refers to the implementation of no-cost or low-cost measures, whereas the second stage refers to the implementation of medium-cost or high-cost measures. In each of the two stages (periods), the ESCO will guarantee a different level of performances in terms of energy cost savings; lower performance guarantees in the first guarantee period and higher performance guarantees in the second guarantee period. The following text has been added to describe the differences among the sub-steps: “The steps followed for deploying the energy efficiency measures begin with an **energy audit**, to analyse energy use in detail and understand the amount of energy consumed across different systems or processes and the identification of areas of inefficiency. The audit provides a baseline and identifies potential opportunities for improvement. Once the audit is complete, the next step is **planning**. The purpose of planning is to decide which measures should be implemented based on their technical feasibility, financial viability, and expected impact. It involves setting priorities, defining targets, estimating costs and savings, and preparing an action plan with clear responsibilities and timelines.  Following the planning, the **implementation** phase involves the practical execution of the selected measures. This involves installing new technologies, upgrading equipment, optimizing processes, or training staff. Finally, **monitoring** involves systematically tracking energy consumption and assessing it against the targets established during the planning phase. Any discrepancies are analysed, and corrective actions are implemented to sustain energy efficiency. Moreover, this activity generates valuable insights and lessons learned that inform future audits and projects, thereby contributing toward establishing a cycle of continuous improvement.”

- The methodology is overly complicated with mathematical models. It would be good to define it in an algorithmic way if possible.

Thank you for your comment. The methodology section has been divided into subsections to enhance readability. Furthermore, an algorithmic presentation has been introduced in section 3.1. This consists in explaining the steps that an ESCO will take to implement the proposed smart energy service in a commercial rented property. High-level methodology to implement the proposed smart energy service This section describes the high-level methodology to be followed by an ESCO that wants to implement the proposed smart energy service in a commercial rented property. The steps to be followed are illustrated in Figure 4 and explained in the following paragraph. The **first step** is to define the project scope and agree on a contractual template. In this step, the ESCO, building owner, and tenants identify the property and its energy profile, agree on the terms of the smart energy service, and establish a governance framework. This framework clearly specifies the responsibilities of each party and determines how energy savings will be measured and allocated. The **second step** is to conduct an energy audit, whereas the **third step** is to identify and plan energy efficiency measures (see section 2.2 and Figure 3). The **fourth step** is to assess risks and determine financial requirements. The ESCO evaluates performance risks and sets guaranteed savings levels. This step also defines investment responsibilities for the ESCO, building owner, and tenants, and ensures that the project is financially viable for all parties involved. Following the risk assessment, a draft Energy Performance Contract (EPC) is prepared ( **fifth step**). This contract includes the scope of the project, the timeline for implementing the energy efficiency measures. the guaranteed performance targets, payment mechanisms, monitoring and verification protocols, and dispute resolution procedures. The draft serves as a starting point for negotiation among all stakeholders. The **sixth step** involves negotiating and finalising the contract. The building owner and tenants review the draft EPC, considering the implications for lease arrangements, cost-sharing, and benefit allocation. Revisions are made until all parties reach mutual agreement on the terms and conditions of the EPC. Finally, the contract is signed, and the project is implemented. This high-level methodology provides a clear and comprehensive roadmap for ESCOs to deliver smart energy services in the commercial rented sector, ensuring that energy efficiency, cost savings, and stakeholder engagement are systematically addressed throughout the project lifecycle. In the next section 3.2, the suggested calculations supporting the financial assessment of the EPC and the decision-making process for its finalisation, will be described. Furthermore, Sections 3.3 and 3.4 present the Business Model Canvas and the Value Proposition Canvas, respectively. Such canvases provide the conceptual framework used to characterize the business model that an ESCO should adopt in order to offer the proposed smart energy service on a commercial basis. Figure 4: Steps for Implementing the proposed smart energy service in a commercial rented property – was added.

- The results include different kinds of mathematical equations which were never introduced in the methodology. It would be good to dedicate a section to introduce these complicated elements.

Thank you for your comment. The mathematical equations in the results section that were not previously introduced in the methodology section, have been moved to the methodology section in the revised version of the paper to improve its organisation. Section 3.2 (divided in subsections) has been dedicated to introducing the different elements of the mathematical model.

- Overall, the study needs to be organized in a way that allows mathematical models and other results to be perceived easily by the readers.

Thanks for your comment. The study has been reorganised in the following manner:

- Section 3.1 *High-level methodology to implement the proposed smart energy service* was added.
- Section 3.2 *Mathematical model used to analyse the factors influencing the decision-making process in relation to Energy Performance contracting for commercial rented buildings* was reorganised placing the different elements of the mathematical model therein included in separate subsections:
  - 3.2.1 Net Present Value Calculations and decision-making processes
  - 3.2.2 Modelling of energy savings
  - 3.2.3 Modelling of energy cost savings
  - 3.2.4. Modelling of operation and maintenance costs
- Section 4.1 *Case study* was added to provide the reader with the details of a reference case study considered throughout section 4.

# Reviewer report: Reviewer response for version 1

**DOI:** 10.21956/openreseurope.20401.r52500

**Published:** 2025-05-19

**Authors:** Paulina Golinska-Dawson

**Affiliations:** 1 Poznan University of Technology, Poznan, Poland

The purpose of this paper is to present an evaluation of the smart energy service for the commercial rented sector, developed as part of the SmartSPIN project. The topic is interesting, however, the paper requires several revisions to improve its clarity and coherence:

- Abstract: The abstract would benefit from more clearly highlighting the research gap and the originality of the proposed approach. Try to explain how the paper contributes to the existing body of literature.
- Sections 2.2 and 2.3: Please strengthen the connection between these two sections. Currently, the Net Present Value (NPV) calculation and the business model canvas description appear somewhat disconnected.
- Structure and flow: The structure of the paper is somewhat confusing, particularly in Section 3. Consider reorganising this section to improve the logical flow. The links between Sections 3.1, 3.2, and 3.3 need to be stronger.
- Figures 1–3: Provide a more detailed discussion of the purpose of Figures 1 to 3.
- Please improve clarity regarding the timing and methodology of the data collection process.
- Tables 1 and 2:
- I recommend converting these tables into more user-friendly graphics, as they are currently difficult to interpret. Additionally, improve the narrative surrounding Tables 1 and 2 to better explain their content and relationship.
- Case Study/Example: It would be beneficial to include a case study or numerical example to demonstrate the application of your quantitative model.
- Conclusions: Please elaborate on the key benefits and limitations of your research. Please discuss the potential future work.

Is the study design appropriate and does the work have academic merit?

Yes

Is the work clearly and accurately presented and does it cite the current literature?

Partly

If applicable, is the statistical analysis and its interpretation appropriate?

Not applicable

Are all the source data underlying the results available to ensure full reproducibility?

Partly

Are the conclusions drawn adequately supported by the results?

Partly

Are sufficient details of methods and analysis provided to allow replication by others?

Partly

Reviewer Expertise:

NA

I confirm that I have read this submission and believe that I have an appropriate level of expertise to confirm that it is of an acceptable scientific standard, however I have significant reservations, as outlined above.

# Response

**Published:** 2025-12-18

**Authors:** Luciano De Tommasi

**Affiliations:** International Energy Research Center, Ireland

The purpose of this paper is to present an evaluation of the smart energy service for the commercial rented sector, developed as part of the SmartSPIN project. The topic is interesting, however, the paper requires several revisions to improve its clarity and coherence:

- Abstract: The abstract would benefit from more clearly highlighting the research gap and the originality of the proposed approach. Try to explain how the paper contributes to the existing body of literature.

Thanks for the comment. The following text has been added to address the comment: **“Results, research gap and originality of the proposed approach** The results of the paper contribute to the existing body of literature in two main ways. First, a quantitative model highlighting the key variables that determine the contractual arrangements and profitability of a performance contract for the parties is thoroughly analysed. Second, a business model for the proposed smart energy service is developed by applying the Business Model Canvas and the Value Proposition Canvas frameworks. This addresses a notable gap in the literature, as no prior studies have established a similar business model in the context of commercial rental properties using the proposed methodology.”

- Sections 2.2 and 2.3: Please strengthen the connection between these two sections. Currently, the Net Present Value (NPV) calculation and the business model canvas description appear somewhat disconnected.

Thanks for your comment. Please note that sections have been renumbered to address the comment by another reviewer, therefore sections 2.2 and 2.3 in the version 1 (the paper that you reviewed) were titled: **2.2 Factors influencing the decision-making process in relation to Energy Performance contracting for commercial rented buildings** **2.3 Business model CANVAS** Section 2 in the version 1 paper was titled **2. Research context and methodology** In version 2 of the paper, Research context and methodology have been separated into sections **2. Research context** and **3. Methodology. **Section 2.2 has become section 3.2.1 (titled: “Net Present Value Calculations and decision-making processes”) in the version 2 of the paper, whereas section 2.3 has become section 3.3 in version 2 of the paper. The following text has been added at the beginning of section 3.3 to strengthen the connection between the two sections: “In the previous section 3.2, the Net Present Value (NPV) calculation for building owners, tenants, and the energy service company for the smart energy service was presented, to allow assessing its financial viability in a rented commercial property. This section builds on that analysis by developing a business model using the Business Model Canvas. For energy service companies seeking to implement the smart energy service, a clear understanding of how to financially evaluate Energy Performance Contracting (EPC) is essential. At the same time, the financial calculations are valuable for landlords and tenants, as they highlight the key variables to consider in order to make well-informed and optimal decisions. This connection between financial assessment and business model design has received limited attention in the literature, and its integration represents a key contribution of this study.”

- Structure and flow: The structure of the paper is somewhat confusing, particularly in Section 3. Consider reorganising this section to improve the logical flow. The links between Sections 3.1, 3.2, and 3.3 need to be stronger.

Thanks for your valuable suggestion. Sections have been reorganised in the revised version of the paper. Sections 3.1, 3.2 and 3.3 of the paper that you kindly reviewed (version 1) have been renumbered 4.2, 4.3 and 4.4 respectively in version 2. In section 4.1 the following text has been added to strengthen the link between section 4.2, 4.3 and 4.4:   “Understanding the factors that determine the financial feasibility and profitability of an energy performance contract, together with the decision-making processes of landlords and tenants, is fundamental for an ESCO seeking to implement a smart energy service in a rented commercial property using energy performance contracting. Analysing these factors allows the ESCO to assess which EPC contracts are economically viable, while ensuring that contract duration, pricing structures, and performance guarantees are consistent with the financial objectives and constraints of its clients. Such an understanding enables the ESCO to formulate a value proposition that is both financially attractive and operationally acceptable to landlords and tenants. In the absence of this quantitative insight, the qualitative components of the Business Model Canvas alone may be insufficient to mitigate the risks associated with implementing the smart energy service.” The text added provides the rationale for including 4.2, 4.3 and 4.4 under the same section 4.

- Figures 1–3: Provide a more detailed discussion of the purpose of Figures 1 to 3.

Thank you for your comment. The purpose of Figures 1 and 2 is to illustrate two possible variants of the business model developed within the SmartSPIN project. The purpose of Figure 1 is to present the case where the building owner finances the implementation of proposed energy efficiency measures using their own sinking fund, while the ESCO guarantees savings and invoices the tenants for the service costs. The purpose of Figure 2 is to show the alternative case where the ESCO finances the upgrades by taking on a bank loan. The purpose of Figure 3 is to illustrate the implementation of the smart energy service proposed by the SmartSPIN project in two stages: in the first stage, no-cost or low-cost measures are applied, followed by medium- and high-cost measures in the second stage. Detailed descriptions of all figures are provided in the text. Captions of Figures 1-3 have been improved to clarify the purpose of the figures: Figure 1: SmartSPIN Business Model – Energy efficiency measures financed by building owner Figure 2: SmartSPIN Business Model – Energy efficiency measures financed by Energy Service Company through a loan arranged with Bank or Lender Figure 3: Implementation of SmartSPIN Smart Energy Service in two stages. Please improve clarity regarding the timing and methodology of the data collection process. Thank you for your comment. Section 2.1 Data collection has been added to clarifiy the timing and methodology of the data collection process. 2.1 Data collection This paper builds on the analysis conducted by the authors in (De Tommasi et al. 2024), which compared eight business models for Energy Performance Contracting applicable to the European commercial rented sector. The research team engaged stakeholders of the commercial rented sector through ten interviews and a focus group to determine, respectively, the high-level requirements for a smart energy service suitable for commercial rented properties and the main features of the business model required to deliver the smart energy service (in addition to provide comments about the progress of the SmartSPIN project). The interviews were held between September 2021 and August 2022. The stakeholders involved are listed in (Agrawal and De Tommasi, 2022). Two focus group meetings were held on 7 ^th^ September 2022 and 29 ^th^ August 2023. The stakeholders who participated in the interviews were invited to participate in the focus group.

- Tables 1 and 2:
- I recommend converting these tables into more user-friendly graphics, as they are currently difficult to interpret. Additionally, improve the narrative surrounding Tables 1 and 2 to better explain their content and relationship.

Thank you for your comment. Indeed, the tables may be hard to read in the online version of the paper. However, they are perfectly readable in the PDF version. It is worth noting that Tables 1 and 2 were created by filling in the Business Model Canvas and Value Proposition Canvas templates with information obtained throughout the research process. The use of these canvases was one of the objectives of this study. They were used because they are standardised and widely accepted tools for designing and presenting business models, providing a clear, visual representation of complex relationships and aligning customer needs with the value offered, while capturing key elements such as revenue streams, costs, and partners. Considered state of the art, these canvases are widely used across industries and innovation projects, ensuring methodological rigor and comparability. Using alternative or unfamiliar infographics could reduce clarity and stakeholder confidence, and developing such alternatives is beyond the scope of this paper. Therefore, we recommend downloading the PDF version of the paper for optimal readability. To better explain the relationship between the business model canvas (Table 1) and the value proposition canvas (Table 2) the following text has been added in section 3.4: “The BMC identifies the value proposition within the broader business model and connects it to the channels through which the ESCO delivers the service, the customer relationships maintained, the resources and partners required to implement and monitor energy efficiency measures, and the revenue streams generated from service fees and energy savings. To refine the value proposition of the proposed service previously identified in the BMC, and ensure that the service offer is aligned with what the customer needs, the Value Proposition CANVAS template is used (Osterwalder et al., 2014).” The narrative surrounding Tables 1 and 2, included respectively in sections 4.4.1 and 4.4.2, has been revised to improve its clarity and better explain the contents included in the two tables. Case Study/Example: It would be beneficial to include a case study or numerical example to demonstrate the application of your quantitative model. Thanks for your valuable suggestion. The case study demonstrating the application of the proposed quantitative model is now introduced in section 4.1 and developed throughout section 4.

- Conclusions: Please elaborate on the key benefits and limitations of your research. Please discuss the potential future work.

Thanks for your valuable suggestions. The following text has been added to the conclusions to clarify the key benefits of the proposed methodology: “The study provides several important *benefits*. First, it establishes a direct connection between the technical-economic assessment of a smart energy service suitable for the commercial rented sector (based on energy performance contracting) and the Business Model Canvas and Value Proposition Canvas frameworks. This novel integration bridges the gap between the quantitative and qualitative dimensions that must be considered to successfully implement such a service. Second, the study delivers actionable insights for key stakeholders (building owners, tenants, and energy service companies) by clarifying contractual arrangements such as contract duration, the service fee to be paid by clients to the ESCO, and the appropriate sharing of energy savings between building owners and tenants. These arrangements are essential to overcoming the split-incentive barrier in rented commercial properties. Finally, the integration of quantitative modelling with business model frameworks creates a replicable methodology that can be applied in future studies and adapted to other sectors, thereby supporting both academic research and the practical implementation of sustainable energy services.” The following text has been added to the conclusions to clarify the limitations of the study and the potential future work: “The main *limitation* of the proposed study is that it did not address the estimation of the rebound-effect parameters ρ (risk tolerance) and φ (maximum rebound) using field data, due to the very limited availability of such data. Although sensitivity analyses were conducted, an empirical practical approach for model parameters estimation needs to be developed. Future research will focus on developing a methodology to empirically estimate these parameters, drawing on approaches such as controlled field trials, statistical modelling of energy use, literature-based calibration, discrete choice surveys, and analysis of real contract uptake and energy consumption data.”

# Reviewer report: Reviewer response for version 1

**DOI:** 10.21956/openreseurope.20401.r52502

**Published:** 2025-04-11

**Authors:** Marcele Elisa Fontana

**Affiliations:** 1 Federal University of Pernambuco, Recife, Brazil

The article is interesting, but I have some specific suggestions for the authors to improve the presentation.

 1- In the introduction, I would like to easily identify the objective and contributions of the work, but I didn't.

 2- I don't see the need for such a long "1.3 Structure of the paper" section, especially describing section 1, which has already happened.

 3- It is difficult to identify: how and when the data was collected; type of data collected; who was involved at each stage. Perhaps separating "Research context" from "methodology" would help the authors explain these details better.

 4- Help the reader by providing a step-by-step methodological guide to be followed to replicate your research.

Is the study design appropriate and does the work have academic merit?

Yes

Is the work clearly and accurately presented and does it cite the current literature?

Yes

If applicable, is the statistical analysis and its interpretation appropriate?

Not applicable

Are all the source data underlying the results available to ensure full reproducibility?

Yes

Are the conclusions drawn adequately supported by the results?

Yes

Are sufficient details of methods and analysis provided to allow replication by others?

Partly

Reviewer Expertise:

The article is interesting and relevant, but I left some recommendations to improve the explanation of the data.

I confirm that I have read this submission and believe that I have an appropriate level of expertise to confirm that it is of an acceptable scientific standard, however I have significant reservations, as outlined above.

# Response

**Published:** 2025-12-18

**Authors:** Luciano De Tommasi

**Affiliations:** International Energy Research Center, Ireland

Dear Reviewer, thanks for your overall positive evaluation of the paper. The manuscript has been accurately revised to address your comments. Detailed answers and changes applied to the text are provided after each reviewer’s comment.

1. In the introduction, I would like to easily identify the objective and contributions of the work, but I didn't.

Thanks for your comment. The objective and contributions of the paper have been added in section 1.3, whose title has been changed into Objective, contributions and structure of the paper. The following text has been added: “The objective of this paper is to analyse the factors influencing the decision-making process which determine the contractual arrangements in an Energy Performance Contract for commercial rented buildings and to establish a business model for a performance-based Smart Energy Service (SES) to be implemented by an Energy Service Company (ESCO) that wishes to make the proposed SES commercially available. The contributions of the paper consist in: 1. Using a financial model based on the calculation of the Net-Present-Value for the building owner, tenants and Energy Service Company (ESCO) to analyse the perspective of each Party when negotiating the terms of an EPC; 2. Using the Business Model Canvas and the Value Proposition Canvas templates to work out all the practical implementation aspects for the proposed business model.”

1. I don't see the need for such a long "1.3 Structure of the paper" section, especially describing section 1, which has already happened.

Thanks for your comment. The description of the structure of the paper has been made more compact. Description of section 1 has been removed as suggested by the reviewer.

1.  It is difficult to identify: how and when the data was collected; type of data collected; who was involved at each stage. Perhaps separating "Research context" from "methodology" would help the authors explain these details better. Thanks for your comment.

Research context and methodology have been separated into separate sections. In the revised version of the paper, ‘Research context’ is section 2, whereas ‘Methodology’ has become section 3. Under section 2, subsection ‘2.1 Data collection’ has been added. The following text has been added in section 2.1 to clarify the aspects of data collection requested by the reviewer: “This paper builds on the analysis conducted by the authors in (De Tommasi et al. 2024), which compared eight business models for Energy Performance Contracting applicable to the European commercial rented sector. The research team engaged stakeholders of the commercial rented sector through ten interviews and a focus group to determine, respectively, the high-level requirements for a smart energy service suitable for commercial rented properties and the main features of the business model required to deliver the smart energy service (in addition to provide comments about the progress of the SmartSPIN project). The interviews were held between September 2021 and August 2022. The stakeholders involved are listed in (Agrawal and De Tommasi, 2022). Two focus group meetings were held on 7th September 2022 and 29th August 2023. The stakeholders who participated in the interviews were invited to participate in the focus group.” The following reference has been added to the references list: AGRAWAL, Ruchi; DE TOMMASI, Luciano. SmartSPIN D2.4. Recommendations for the SmartSPIN service. 2022. Reference Source

1. Help the reader by providing a step-by-step methodological guide to be followed to replicate your research.

Thanks for your comment. Section 7 titled: “Replicability of the study” has been added to provide the readers with detailed information on how to replicate the research. The following text has been added: “To replicate the study described in this paper, it is recommended to start with a thorough review of the literature to determine the financial models which apply to energy performance contracting in the commercial rented sector. Such models should be thoroughly analysed to understand the rational behaviour of stakeholders (landlords, tenants and ESCOs) using the lens of utility maximization and find out how each of them acts in their own best interest, based on the economic value of the service for them, incentives (if any), constraints, attitude to consume energy, risk propensity. This analysis should lead to a better understanding of how the stakeholders interact with each other and of the decision-making processes that may lead to the signature of an energy performance contract. After reviewing and analysing the financial models, the research team will organise a brainstorming session, where they will fill in the templates that were selected to develop the business model (e.g. CANVAS). They will consider the perspective and the requirements of the stakeholders, as obtained from previously held interviews and/or focus groups. After completing a draft of the business model, one or more members of the research team will improve its representation making it more compact, clear and immediate to read, e.g., removing repetitions or marginally relevant entries in the CANVAS tables, while making sure that the main aspects required by each section of the template are addressed.”
